# Carbapenem resistance mediated by *bla*_NDM-13_ in a highly drug-resistant *Salmonella* Stanley ST29 strain in China

**DOI:** 10.1128/spectrum.03207-24

**Published:** 2026-06-09

**Authors:** Juanjuan Zhou, Junmei Yang, Ke Li, Hongna Shi, Kaijie Gao, Peipei Zhao, Lu Xu, Dongyu Zhang, Minghui Zhen

**Affiliations:** 1 Department of Clinical Laboratory, Children’s Hospital Affiliated to Zhengzhou University, Henan Children’s Hospital, Zhengzhou Children’s Hospital, Zhengzhou Key Laboratory of Children’s Infection and Immunity, Zhengzhou, People's Republic of China; Guizhou Medical University, Guiyang, China; Chinese Academy of Sciences, Wuhan Institute of Virology, Wuhan, Hubei, China; Zhejiang Chinese Medical University School of Basic Medical Sciences, Hangzhou, Zhejiang, China

**Keywords:** *Salmonella *Stanley, *bla*_NDM-13_, carbapenem resistance, plasmid, horizontal gene transfer

## Abstract

The rise of carbapenem-resistant *Enterobacterales* presents a substantial global public health challenge. While carbapenem-resistant *Salmonella* is rarely reported in clinical settings, this study characterizes a carbapenem resistance mediated by *bla*_NDM-13_ *Salmonella* Stanley strain SAL22057, isolated from the fecal sample of a pediatric patient with diarrhea and fever. We determined antimicrobial susceptibility, conducted genomic characterization, and assessed plasmid conjugation. Antimicrobial susceptibility testing showed that SAL22057 was resistant to meropenem (minimum inhibitory concentration [MIC] 32 μg/mL) and imipenem (MIC 16 μg/mL). Genotyping analysis identified SAL22057 as belonging to ST29, and it was found to be multidrug-resistant and to carry numerous virulence genes. Whole-genome sequencing and plasmid analysis identified that *bla*_NDM-13_ was located on pSAL22057-NDM (IncI1*α*), while a distinct plasmid, pSAL22057-OXA (IncHI2), harbored multiple antimicrobial resistance genes, including *bla*_OXA-10_. Conjugation experiments revealed that *bla*_NDM-13_ was transferable to *Escherichia coli* C600. We show that IS*1294* likely mediates *bla*_NDM-13_ mobilization, with its insertion influencing gene transfer. Furthermore, the consistent flanking of several resistance genes by IS*26* elements indicates that IS*26*-mediated horizontal gene transfer is a key mechanism driving the dissemination of these determinants and genomic rearrangements within *Enterobacteriaceae*.

**IMPORTANCE:**

The emergence of carbapenem-resistant *Enterobacterales* poses a global health threat. This study identified a carbapenem-resistant *Salmonella* Stanley strain, SAL22057, from a pediatric patient, which carried the carbapenemase gene *bla*_NDM-13_. The genotyping revealed that SAL22057 is ST29, displaying a concerning multidrug-resistant phenotype along with several virulence determinants. Alarmingly, this strain exhibits high-level resistance to meropenem (minimum inhibitory concentration [MIC] 32 μg/mL) and imipenem (MIC 16 μg/mL). Conjugation experiments confirm that *bla*_NDM-13_ is transferable to *Escherichia coli* C600, signaling a clear pathway for resistance dissemination among *Enterobacteriaceae*. We demonstrate that IS*1294* likely mobilizes *bla*_NDM-13_, while IS*26* consistently flanks multiple resistance genes, providing mechanistic evidence that IS*26*-mediated transposition is accelerating the spread of resistance genes in clinical pathogens.

## INTRODUCTION

*Salmonella* spp. are common pathogens that infect humans when contaminated food or water is consumed. *Salmonella* is a significant contributor to bacterial food poisoning on a global scale, frequently ranking as the most prevalent bacterial foodborne illness in many countries (1). In recent years, there have been reports of sporadic cases of carbapenem-resistant *Salmonella* spp*.* carrying the *bla*_KPC_, *bla*_IMP_, *bla*_NDM_, *bla*_VIM_, and/or *bla*_OXA-48_-like genes (2). The emergence of carbapenem-resistant *Salmonella* spp*.* presents a significant threat to public health.

The prevalence of metallo-*β*-lactamases (MBLs) remains a major clinical challenge: the ability of these enzymes to hydrolyze *β*-lactams is unaffected by available *β*-lactamase inhibitors (e.g., avibactam, relebactam, and vaborbactam) (3). This often leads to a range of infections associated with high mortality rates (4). NDM has emerged as the predominant type of MBL among clinical isolates of *Enterobacterales*. The NDM-13 variant was initially discovered in a multidrug-resistant *Escherichia coli* (5). In addition, class D *β*-lactamases, such as OXA-10, while primarily conveying resistance to penicillin (PIP) and early cephalosporins, have also been reported to exhibit weak carbapenem-hydrolyzing ability under certain conditions (6, 7). The co-production of NDM-13 with other *β*-lactamases in *Salmonella* spp. is uncommon. In this study, we report a community-acquired *Salmonella* Stanley strain, SAL22057, isolated from a child with acute diarrhea and fever, which coharbored *bla*_NDM-13_ and *bla*_OXA-10_.

## RESULTS

### Isolate characteristics

*S*. Stanley SAL22057 was recovered from a fecal sample from an 18-month-old boy who was admitted to a children’s hospital in Zhengzhou, China, for diarrhea and fever in June 2022. Ten days prior to admission, the child developed diarrhea and fever (peak temperature: 39.2°C) without an identifiable cause. Despite intravenous administration of cefoperazone-sulbactam (0.5 g q12h) for 4 days at another hospital, high fever persisted. The child was admitted to our hospital with chief complaints of diarrhea and fever lasting 10 days. On the second hospital day, *Salmonella* was isolated from stool culture. Antimicrobial therapy was switched to meropenem (MEM) (0.24 g q8h). After 8 days of MEM treatment, body temperature normalized, and the infection was controlled. Given that the isolate SAL22057 was resistant to MEM *in vitro*, the favorable clinical outcome may have been achieved by a prolonged treatment course.

This isolate was resistant to PIP, piperacillin-tazobactam (TZP), amoxicillin-clavulanic acid (AMC), ceftazidime (CAZ), cefotaxime (CTX), cefepime (FEP), imipenem (IPM), MEM, chloramphenicol (CHL), and trimethoprim-sulfamethoxazole (SXT); intermediate to ciprofloxacin (CIP) and levofloxacin (LVX); while still susceptible to aztreonam (ATM), polymyxin B (POL), and tigecycline (TGC) (Table 1). Imipenem-3-aminobenzeneboronic acid/EDTA double disk synergy test demonstrated the presence of metallo-carbapenemase in SAL22057, explaining the carbapenem-resistant phenotype. The slide agglutination technique showed that SAL22057 was Stanley serotype (8). PCR screening for carbapenemase genes, followed by sequencing, led to the detection of *bla*_NDM-13_ in SAL22057 (Table 1). Conjugation assay results demonstrated that the *bla*_NDM-13_ was successfully transferred to the recipient *E. coli* C600. The conjugation frequencies were approximately 6.7 × 10^−5^ at 37°C, 1.3 × 10^−4^ at 30°C, and 3.1 × 10^−4^ at 25°C. Compared to 37°C, the conjugation frequencies at 30°C and 25°C were approximately 1.9-fold and 4.6-fold higher, respectively. All *bla*_NDM-13_-positive transconjugants obtained at temperatures exhibited resistance to both MEM and IPM, with consistent minimum inhibitory concentrations (MICs) of 16 μg/mL for both MEM and IPM. The MEM and IPM MICs of transconjugants were more than 32-fold higher than those of the recipient *E. coli* C600 (Table 1). These MICs were at least 64-fold higher than those of the plasmid-free recipient *E. coli* C600. While the *bla*_OXA-10_ proved non-transferable to recipient *E. coli* C600 at 25°C, 30°C, or 37°C.

**TABLE 1:** Minimal inhibitory concentrations (MICs) of *S.* Stanley SAL22057 clinical isolate, transconjugants, and recipient strain (μg/mL)

	*S.* Stanley SAL22057 (donor strain)	SAL22057-*E. coli* C600 (transconjugants, 37°C)	SAL22057-*E. coli* C600 (transconjugants, 30°C)	SAL22057-*E. coli* C600 (transconjugants, 25°C)	*E. coli* C600
Antimicrobial resistance gene(s)	*bla*_NDM-13_, *bla*_TEM-1_, *bla*_OXA-10_, *dfrA14, qnrS1, sul3, aac(6')-Iaa, aadA1, aadA2, aadA22, aph(3'')-Ib, aph(6)-Id, aph(3')-Ia, aph(4)-Ia, aac(3)-IV, cmlA1,* tet*(A)*	*bla*_NDM-13_	*bla*_NDM-13_	*bla*_NDM-13_	–^*a*^
Antimicrobials					
PIP	>128	>128	>128	>128	≤4
TZP	>128/4	>128/4	>128/4	>128/4	≤4/4
AMC	>32/16	>32/16	>32/16	>32/16	4/2
CAZ	>64	>64	>64	>64	0.5
CTX	>64	>64	>64	>64	≤1
FEP	>32	16	32	32	≤0.12
ATM	≤1	≤1	≤1	1	≤1
IPM	16	16	16	16	≤0.25
MEM	32	16	16	16	≤0.25
LEV	1	1	1	1	0.5
CIP	0.5	≤0.25	≤0.5	≤0.5	≤0.25
SXT	>16/304	≤1/19	≤0.25	≤0.25	≤1/19
CHL	32	4	4	4	4
POLB	0.25	0.25	0.25	0.25	0.25
TGC	0.125	0.125	0.25	0.25	0.125
Conjugation					
Replicate counts (*n*)	NA^*b*^	3	3	3	NA
Conjugation frequency (mean)	NA	~5.2 × 10^−5^	~1.4 × 10^−4^	~2.3 × 10^−4^	NA
Conjugation frequency fold change (vs 37°C)	NA	NA	~2.7	~4.4	NA

^*a*^ –, no data.

^*b*^ NA, not available.

### Genetic analysis of SAL22057 strain

The genome of strain SAL22057 comprised a single chromosome (4,847,759 bp, 52.2% G+C) and six plasmids. Multilocus sequence typing showed that *S*. Stanley SAL22057 belonged to ST29. Eleven *Salmonella* pathogenicity islands (SPIs) were identified, encompassing SPI-1 to SPI-5, SPI-9, SPI-12 to SPI-14, C63PI, and CS54 island. Based on virulence factors of pathogenic bacteria (VFDB) annotation, 266 virulence genes were predicted in total, including 87 secretion system genes, 78 fimbria adherence determinants, 52 peritrichous flagella, 25 metabolic factors, and 6 immune modulation genes (Table S1). Functional analysis revealed SPI-1 encoded 30 type III secretion system (T3SS) effectors; SPI-2 carried 28 T3SS-associated virulence factors; SPI-3 contained *mgtBC* and *misL*; SPI-4 harbored *STM4261*; SPI-5 possessed *sopB/sigD*; SPI-12 included *sspH2*; CS54 island carried *shdA*, *ratB*, and *sinH*; C63PI contained *sitC*. No virulence factors were annotated in SPI-9, SPI-13, or SPI-14 under current database criteria. A total of 18 antimicrobial resistance genes (ARG) were identified: *aac(6*′*)-Iaa* gene (intrinsic in *Salmonella*) on the chromosome, *bla*_NDM-13_ and *ble*_MBL_ on pSAL22057-NDM (IncI1α 87,928 bp) and 15 other ARG on pSAL22057-OXA (IncHI2, 237,376 bp). The remaining plasmids—pSAL22057-3 (IncY, 93,293 bp), pSAL22057-4 (IncQ1, 6,477 bp), pSAL22057-5 (Col440I, 5,423 bp), and pSAL22057-6 (ColRNAI, 4,583 bp)—carry no additional resistance genes.

### Characteristics of pSAL22057-NDM

**Fig 1:**
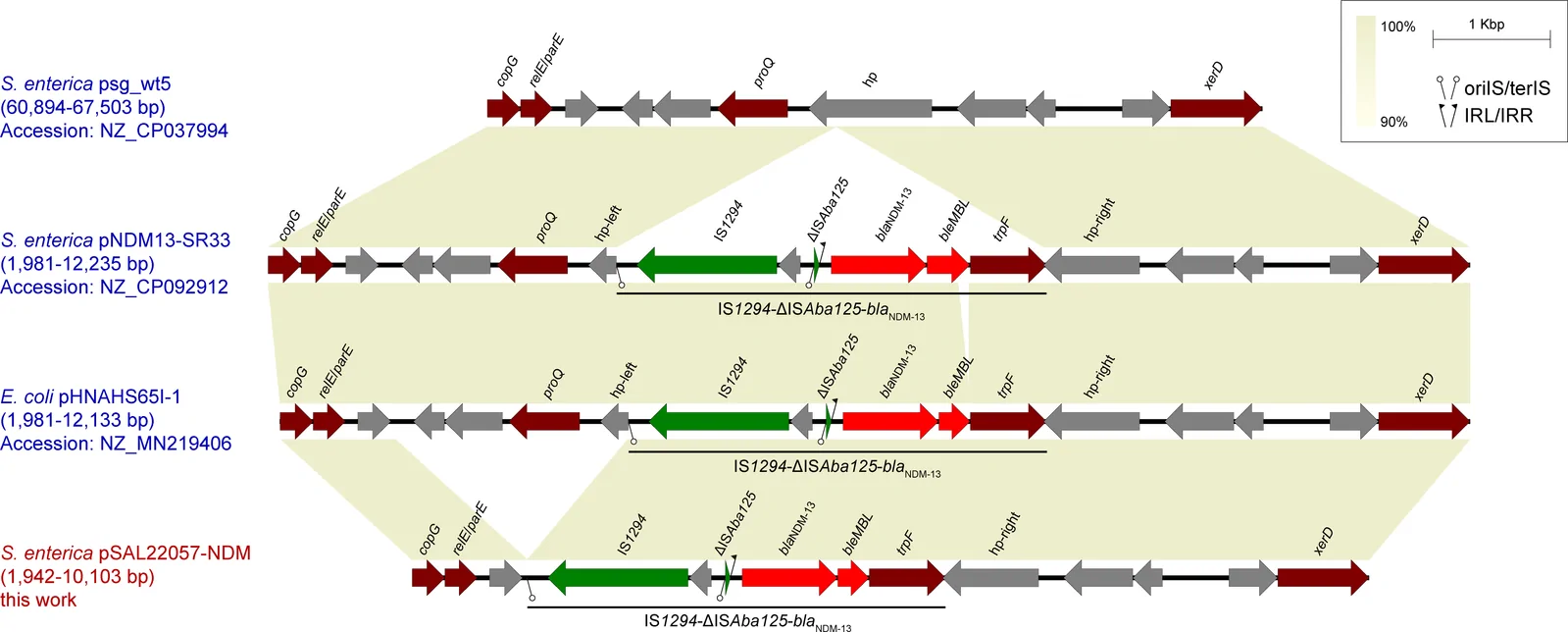
Schematic illustration comparing part of the structural features of pSAL22057-NDM with corresponding regions of pHNAHS65I-1 (MN219406), pNDM13-SR33 (CP092912), and psg_wt5 (CP037994). IS*1294*–ΔIS*Aba125–bla*_NDM-13_ comprises IS*1294*, ΔIS*Aba125*, *bla*_NDM-13_, *ble*_MBL_, and *trpF*. Positions of the 51-bp IRR fragment of ΔIS*Aba125* and the *terIS* region of IS*1294* are 4,612–4,662 and 4,586–4,611, respectively, in reference plasmid NZ_CP195667. The 241-bp hp-left is located at positions 4,713–4,953 bp in both pNDM13-SR33 and pHNAHS65I-1, directly adjacent to the *oriIS* region (4,954–4,974 bp). In pSAL22057-NDM, insertion of this module caused deletion of hp-left, *proQ*, and two additional hypothetical proteins, representing the loss of four ORFs. In addition, pSAL22057-NDM and pHNAHS65I-1 carry a 102-bp deletion within *ble*_MBL_ relative to Tn*125* (HQ857107) and pNDM13-SR33. The linear map was generated using EasyFig. ORFs are portrayed by arrows and colored according to their putative functions: red, resistance genes; fuchsia, replication and initiation protein; green, IS; blue, integrase; purple, metal resistance related genes; orange, conjugation transfer related protein; maroon, other functional protein; gray, hypothetical protein.

The complete sequence of plasmids pSAL22057-NDM (NZ_CP195667) and pSAL22057-OXA (NZ_CP195667) was analyzed. The *bla*_NDM-13_ was located on a pSAL22057-NDM, with a GC content of 50.59%. The conjugation-associated modules were detected in pSAL22057-NDM, like the *ori*T, relaxase *nik*B, T4CP gene *trb*C, and T4SS *tra* family genes. The BLAST analysis revealed that pSAL22057-NDM exhibited high similarity to *E. coli* pHNAHS65I-1 (MN219406; 98.00% coverage, 100.00% identity), *Salmonella enterica* pNDM13-SR33 (CP092912; 98.00% coverage, 99.99% identity), and psg_wt5 (CP037994; 93.00% coverage, 99.75% identity) (Fig. S1). The primary distinction was that psg_wt5 lacked a conserved IS*1294*–ΔIS*Aba125-bla*_NDM-13_ present in the other related plasmids (Fig. 1). In pSAL22057-NDM, insertion of IS*1294* into IS*Aba125* generated a 51-bp right-end inverted repeat (IRR) of IS*Aba125* that contained the promoter region, with the *terIS* region of IS*1294* located immediately adjacent to this ΔIS*Aba125*. IS*1294*–ΔIS*Aba125-bla*_NDM-13_ module was inserted into an encoding region of a hypothetical protein gene upstream of *proQ* in pNDM13-SR33 and pHNAHS65I-1, disrupting the coding region. The insertion resulted in the hypothetical protein being split into two segments, designated hp-left and hp-right (details in Fig. 1 legend). Notably, in pSAL22057-NDM, insertion of IS*1294*–ΔIS*Aba125–bla*_NDM-13_ module led to a large deletion compared with pNDM13-SR33 and pHNAHS65I-1.

### Characteristics of pSAL22057-OXA

**Fig 2:**
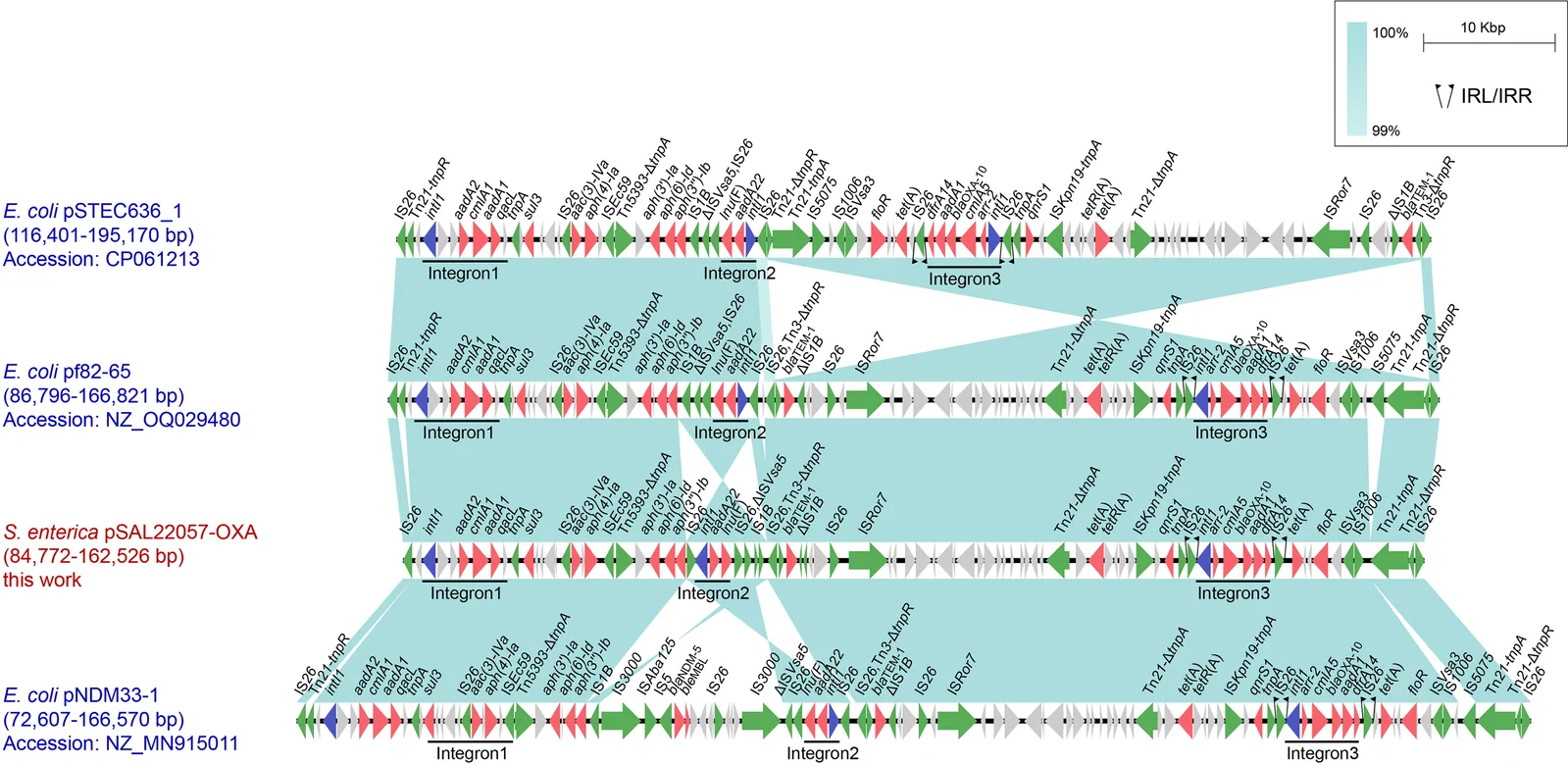
Schematic illustration comparing the structural features of pSAL22057-OXA with pSTEC636_1 (CP061213) and pf82-65 (NZ_OQ029480).

BLAST analysis revealed that pSAL22057-OXA exhibited high similarity to the plasmids, including *E. coli* pSJ_255 (CP011062, 90.00% coverage and 99.87% identity), pLH30-mcr1 (CM008265, 85.00% coverage and 99.98% identity), and pNDM33-1 (MN915011, 99.00% coverage and 99.97% identity), which share a highly conserved backbone (Fig. S2). Linear comparison identified a 77,804-bp region (84,772–162,526 bp) in pSAL22057-OXA that is shared with pf82-65 (OQ029480), pNDM33-1, and pSTEC636_1 (CP061213) (Fig. 2). This region is bounded by IS*26* on both sides and contains three integron structures (integron 1, 2, 3) densely populated with resistance genes, ISs, and transposons. Notable mobile genetic elements are present in this region. Resistance genes are distributed across the three integron structures. Integron 1 carries *aadA2*, *cmlA1,* and *aadA1*. Integron 2 carries *lnu*F and *aadA22*, with *bla*_TEM-1_ nearby. A distinct resistance cluster comprising *sul3*, *aac(3)-Iva*, *aph(4)-Ia*, *aph(3*′*)-Ia*, *aph(6)-Id*, and *aph(3*″*)-Ib* is situated in the region between integrons 1 and 2. Integron 3 features a cassette array of *arr-2*, *cmlA5*, *bla*_OXA-10_, *aadA1,* and *dfrA14*, flanked by *tetR(A*), *tnpA* of Tn*2*, *tet*(A), and *floR*. Additionally, integron 3, which contains *bla*_OXA-10,_ is flanked by directly oriented IS*26* sequences. Notably, pf82-65 and pNDM33-1 exhibit an inversion of the integron 2 region relative to pSAL22057-OXA. Furthermore, pNDM33-1 possesses an additional IS*3000*-associated transposable region inserted between IS*1B* and ΔIS*Vsa5* near the inverted integron 2, which harbors the *bla*_NDM-5_ gene (Fig. 2). A variety of conjugation-associated modules were identified in pSAL22057-OXA, including *oriT*, *nikB*, T4CP, and T4SS family genes.

### Comparative genomic and plasmid analysis of *bla*_NDM-13_ and *bla*_OXA-10_ in *Salmonella strains*

**Fig 3:**
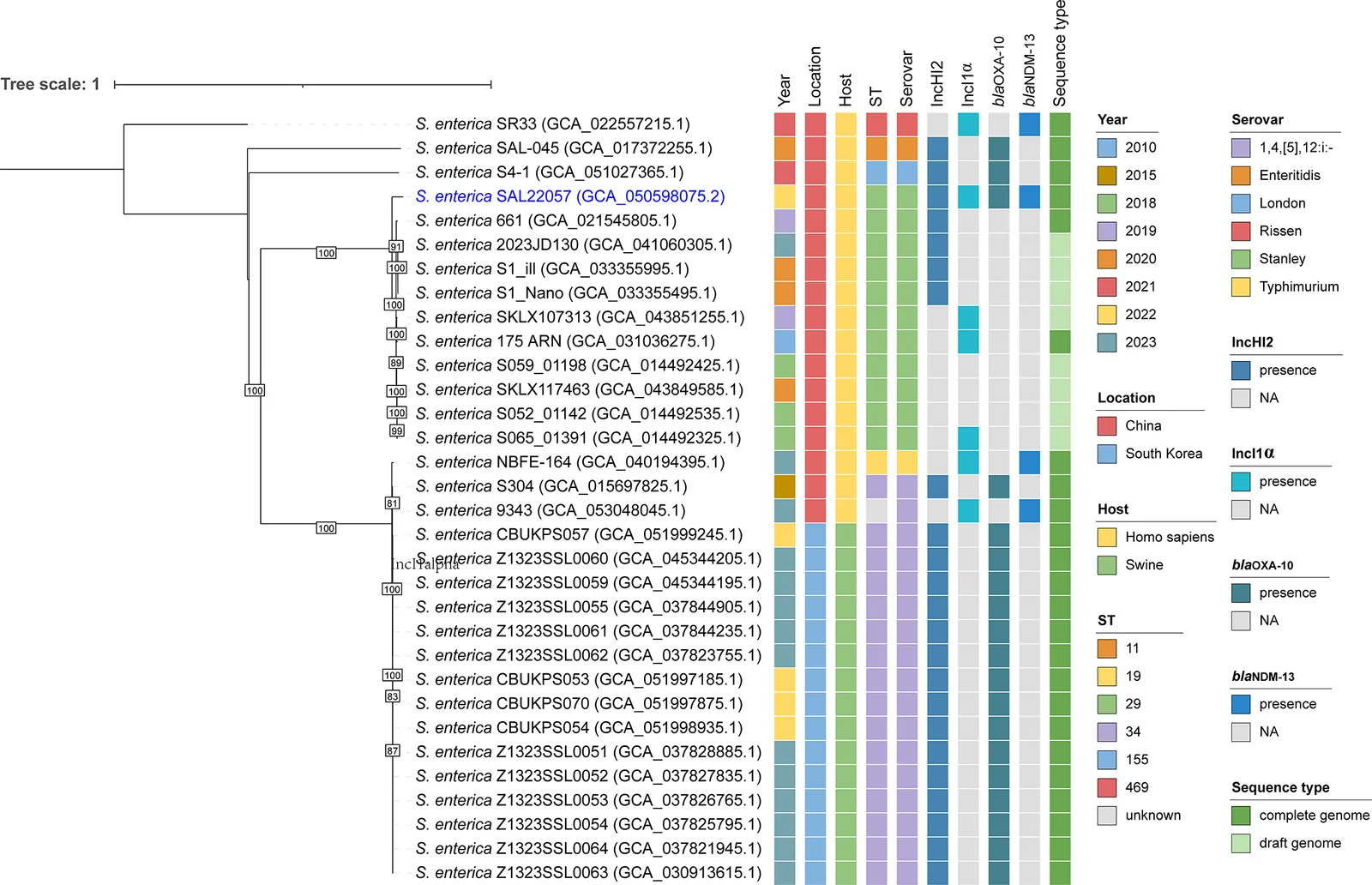
Comparative genome analysis of SAL22057 and other *Salmonella* strains based on SNPs. The sequence type, serovar, geographic origin, host, year of isolation, and plasmid replicon type are shown.

To elucidate the phylogeny of *Salmonella* harboring IncI1α-NDM-13 and IncHI2-OXA-10 plasmids, we built a core-genome SNP phylogenetic tree. The tree included strains with related plasmids retrieved from NCBI, augmented by relevant ST29 strains for contextual analysis. The ST29 strain SAL22057 isolated in this study exhibits two distinctive features. Among the four *bla*_NDM-13_-carrying *Salmonella* strains documented worldwide, SAL22057 represents the sole isolate belonging to ST29. Phylogenetic analysis revealed that the included strains, primarily originating from human samples in China and swine samples in South Korea, segregated into two major clusters (Fig. 3 and Table S2). Cluster I consisted exclusively of ST29 serovar Stanley strains. Cluster II was predominantly composed of ST34 serovar 1,4,[5],12: i: - strains. Analysis of plasmid distribution showed that among the 20 strains carrying IncHI2-type related plasmids, 16 belonged to ST34 serovar 1,4,[5],12: i: -, two were ST29 Stanley, and the remaining two were identified as ST11 serovar Enteritidis and ST155 serovar London, respectively. Among the five strains harboring IncI1α-related plasmids, two were the ST29 serovar Stanley. The remaining three comprised single strains of ST469 Rissen, an unknown ST serovar 1,4,[5],12: i: -, and ST19 serovar Typhimurium. Notably, IncHI2 or IncI1α plasmids were detected in the genomic sequences of multiple reference ST29 strains. However, apart from SAL22057 from this study, none of the other ST29 strains—including strain 661 carrying the IncHI2 pSal661_2 and strain 175 ARN carrying the IncI1α p175-CMY-102k—were found to carry the *bla*_NDM-13_ or *bla*_OXA-10_.

## DISCUSSION

The *bla*_NDM-13_ gene was first identified in 2015 on the chromosome of an *Escherichia coli* isolate from Nepal (5). Among NDM-type carbapenemases, *bla*_NDM-1_ and *bla*_NDM-5_ have been most commonly detected in *Salmonella*. The first example of *bla*_NDM-1_ in *Salmonella* was likely acquired in India but was identified in the United States in 2011 (9). The *bla*_NDM-1_ and *bla*_NDM-5_ genes were detected in two *S*. Stanley isolates, each located on a distinct plasmid: *bla*_NDM-1_ was found on an IncC plasmid in a human-derived isolate in Zhejiang, China (10), while *bla*_NDM-5_ was carried on an IncHI2 plasmid in an isolate from a slaughterhouse in Guangzhou, China (11). This report describes the identification of *bla*_NDM-13_ in *S*. Stanley strain SAL22057. The isolation of this multidrug-resistant *S*. Stanley ST29 strain, a known foodborne pathogen with confirmed virulence factors, underscores its considerable potential for causing disease. Although IncHI2 and IncI1α plasmids can colonize the ST29 lineage, the acquisition of the *bla*_NDM-13_ is not a widespread phenomenon and most likely represents a recent, independent event mediated by horizontal gene transfer.

*S*. Stanley causes salmonellosis outbreaks in humans by contaminating food-producing animals, eggs, dairy products, fruits, and vegetables (12). The emergence of multidrug-resistant *S*. Stanley, coupled with its significance as a foodborne pathogen, will compound public health threats. According to annotations from the VFDB database, SAL22057 possesses virulence genes primarily associated with fimbriae, T3SS, and adhesion systems, all of which play critical roles in *Salmonella* pathogenesis (13). Overall, the multidrug-resistant *S*. Stanley SAL22057 contains significant pathogenicity islands and a variety of virulence-associated genes, highlighting its pathogenic potential.

NDM-type MBLs (e.g., NDM-13) cannot hydrolyze ATM (5), which supports the consistency between our isolate’s ATM-susceptible phenotype and its NDM-harboring genotype. Subsequent surveillance has revealed the dissemination of *bla*_NDM-13_ among clinically important *Enterobacteriaceae*, with *bla*_NDM-13_ being carried by diverse plasmid types (including IncX3, IncFIB, and IncI1) in both *E. coli* and *Salmonella enterica* isolates (14). PSAL22057-NDM exhibits high similarity with pHNAHS65I-1, pNDM13-SR33, and psg_wt5. PHNAHS65I-1 and pNDM13-SR33 are classified as IncI1-type plasmids originating from China. PHNAHS65I-1 was carried by an *E. coli* isolate, and it was identified in animal foodstuff. The transfer of the *bla*_NDM-13_-harboring pSAL22057-NDM to *E. coli* C600 was experimentally verified through conjugation assays in this study. The conjugation frequencies were approximately 1.9-fold higher at 30°C and 4.6-fold higher at 25°C than at 37°C, demonstrating that pSAL22057-NDM transfers more efficiently at around 25°C–30°C. Therefore, combined with the evidence of *bla*_NDM_ transmission from animal-derived food to humans through epidemic plasmids (15), the *bla*_NDM-13_-carrying plasmid identified in this study may represent a similar dissemination risk within the food chain.

Analysis of the pSAL22057-NDM revealed a novel IS*1294*–ΔIS*Aba125-bla*_NDM-13_ mobile module, which was absent in the psg_wt5. IS*1294* might play a role in the mobilization and spread of *bla*_NDM-13_ (8). Unlike previously described Tn*125*-like elements, this module exhibits preferential insertion into a conserved hypothetical protein gene upstream of *proQ*, a feature that may reflect sequence-specific integration preferences. The truncation of IS*Aba125* to a 51-bp IRR fragment (containing the promoter region) highlights a key mechanism by which IS*1294* reshapes resistance gene expression, a finding that extends the understanding of IS-mediated promoter capture in carbapenem resistance. Notably, the unique deletion event in pSAL22057-NDM (loss of four ORFs, including hp-left, *proQ,* and two additional hypothetical proteins) underscores the genomic plasticity of these plasmids during mobile element integration. While a 25-bp sequence was previously proposed to facilitate homologous recombination, reanalysis revealed no significant homology supporting this model; instead, the insertion site specificity likely stems from IS*1294*’s intrinsic target site preferences, a characteristic consistent with known IS element behavior. These observations collectively reinforce the role of IS*1294* as a critical driver of *bla*_NDM-13_ mobilization in clinical *Enterobacterales*.

### Conclusion

We’ve identified a carbapenem-resistant *S*. Stanley isolate, SAL22057(ST29), harboring *bla*_NDM-13_, a key carbapenemase gene in this strain. We characterized pSAL22057-NDM, the plasmid harboring *bla*_NDM-13_, and found that IS*1294* likely plays a key role in mobilizing this gene, with its insertion influencing how the gene transfers. The presence of *bla*_NDM-13_, along with other multidrug resistance determinants*,* in a virulent, multidrug-resistant *S*. Stanley ST29 strain underscores its pathogenic potential and the urgent need for enhanced surveillance in human and animal populations.

## MATERIALS AND METHODS

### Bacterial strain

An isolate designated SAL22057 was obtained from an 18-month-old boy’s fecal sample at a children’s hospital in June 2022. Species identification of SAL22057 was performed using the Bruker Biotyper MALDI-TOF MS (Bruker Daltonik GmbH, Bremen, Germany) and confirmed by 16S rRNA gene sequencing. Clinical features of the patient were obtained from the electronic medical records. Its serotype was confirmed using the slide agglutination technique (16).

### Antimicrobial susceptibility testing

The MICs for IPM, MEM, CAZ, CTX, FEP, TZP, AMC, ATM, PIP, SXT, LVX, CHL, and CIP were determined using the broth microdilution method in accordance with the guidelines of the Clinical and Laboratory Standards Institute (17). The assay was performed in triplicate using cation-adjusted Mueller-Hinton broth and incubated at 35°C for 18–20 hours in ambient air before reading. *E. coli* ATCC 25922 was utilized as a control, with three independent replicates. The production of carbapenemase was phenotypically screened using a combined disk test. An IPM (10 µg) disk was placed in the center of a Mueller-Hinton agar plate, seeded with a 0.5 McFarland suspension of the test isolate. Disks containing EDTA (750 µg) and 3-amino benzene boronic acid (400 µg) were placed 20 mm apart, center to center, from the imipenem disk. After incubation at 35°C for 16–20 hours, a positive result for carbapenemase production was defined as a ≥5 mm increase in the zone diameter of inhibition between the imipenem disk and either of the synergistic agent disks compared to the imipenem disk alone (18).

### Identification of resistance genes

The carbapenemase genes (*bla*_NDM_*, bla*_KPC_*, bla*_VIM-1_-like*, bla*_IMP-4_-like, and *bla*_OXA-48_-like genes) were detected by PCR-based sequencing, as previously described (19). All PCR-positive samples were sequenced, and DNA sequences were compared with those available in the NCBI GenBank database (https://www.ncbi.nlm.nih.gov/genbank/) using BLAST.

### Whole-genome sequencing and bioinformatics analysis

Genomic DNA was extracted using the SDS method to determine the complete nucleotide sequence of SAL22057 (20). The whole genome of SAL22057 was sequenced on the PacBio Sequel platform (Pacific Biosciences, California, United States) and Illumina NovaSeq PE150 (Illumina, San Diego, CA, United States). High-quality DNA samples were utilized to create both large (10–20 kb) and small (~400 bp) fragment libraries, which were sequenced on multiple platforms to acquire raw data. Long reads were independently assembled using Canu 2.0 (21) (min Read Length = 1,000; corrected Error Rate = 0.01; min Overlap Length = 500; cor Out Coverage = 50) and Flye (v2.8.3) (default parameters). QC-passed short reads were assembled with SOAPdenovo (v2.04) (22) (k-mer: 69–127; optimally assembled) and gaps closed using GapCloser (v1.12). Assemblies were evaluated by self-comparison (removing redundant contigs) and cross-comparison of Canu/Flye results (confirming replicon structure and selecting the most contiguous assembly). Putative circular replicons were identified via terminal fragment BLAST analysis. Short reads were mapped to long-read assemblies to detect potential small-plasmid loss and assess accuracy/completeness. Final replicons were polished with Pilon (v1.24) (23) using Illumina data. Uniform read coverage across all replicons validated assembly completeness and accuracy at the sequencing level.

The genome was annotated using RAST version 2.0 (https://rast.nmpdr.org) and BLAST (https://blast.ncbi.nlm.nih.gov/Blast.cgi). Antimicrobial resistance genes and plasmid replicons were identified using online tools (https://www.genomicepidemiology.org/). Virulence genes were analyzed using the VFDB (https://cge.food.dtu.dk/services/VirulenceFinder/) with BLASTn, applying a threshold of >70% identity and >70% coverage. Resistance genes were identified using AMRFinderPlus (https://www.ncbi.nlm.nih.gov/pathogens/antimicrobial-resistance/AMRFinder/). The sequence type (ST) was determined using MLST (https://genepi.dk/mlstfinder). The presence of Salmonella pathogenicity islands (SPIs) was detected by SPIFinder (https://cge.food.dtu.dk/services/SPIFinder/). A search for transposons and IS elements was performed using the IS Finder database (https://www-is.biotoul.fr/), which contains only a small number of selected transposons. Plasmid comparisons were performed using BLAST Ring Image Generator (http://brig.sourceforge.net) (24) and Easyfig tools (http://mjsull.github.io/Easyfig) (25).

### Conjugation assay

Overnight cultures of the plasmid-free recipient *E. coli* C600 and the donor *S*. Stanley SAL22057 were prepared by incubating each in 5 mL Lysogeny Broth (LB) at 37°C for 18 hours. Conjugation assays were performed by mixing the recipient and donor cultures at a 1:1 ratio in 5 mL of fresh LB without shaking for 18 hours. The mixtures were then incubated in parallel at three different temperatures: 25°C, 30°C, and 37°C, for 18 hours each, with three independent replicates per condition. Following incubation, transconjugants were selected on Mueller-Hinton agar (OXOID, Hampshire, United Kingdom) supplemented with 200 mg/L rifampicin (Meilunbio, Dalian, China) and 32 mg/L CHL (Meilunbio, Dalian, China). To investigate the potential horizontal transfer of *bla*_NDM-13_ and *bla*_OXA-10_, three transconjugants from each condition were subjected to PCR and antibiotic susceptibility testing. Replicon typing was performed to assess the presence of the IncI1α and IncHI2 plasmids in the transconjugants (26). The presence and functionality of transfer origins (oriT) for pSAL22057-NDM and pSAL22057-OXA were identified by the oriTfinder (https://bioinfo-mml.sjtu.edu.cn/oriTfinder/).

### Comparing gene cluster analysis

Reference sequences for each fragment were chosen based on high similarity (≥95% identity, >90% coverage), matching strain or plasmid background, and completeness, with final validation through Easyfig alignment. The resultant images were generated and refined utilizing AI technology.

### Comparative phylogenomic and epidemiologic analysis

Based on the complete sequences of pSAL22057-NDM and pSAL22057-OXA, homologous sequences were identified via BLASTn searches against the NCBI non-redundant nucleotide database. Whole-genome sequences of source strains carrying these related plasmids were subsequently downloaded from PCBI-Pathogen, along with genome sequences of *Salmonella* ST29 obtained in the PATRICBI database. Together with SAL22057, these sequences were analyzed using kSNP3 (27). A phylogenetic tree was constructed using PhyML based on concatenated, qualified core genome SNPs, with the whole genome SAL22057 as the reference. The tree was visualized and annotated with epidemiological metadata (sequence type, serovar, geographic origin, host, year of isolation, and plasmid replicon type) using the iTOL (https://itol.embl.de).

## Supplementary Material

Reviewer comments

## Data Availability

The complete sequences of SAL22057 were submitted to the NCBI GenBank database with the following accession numbers: chromosome, NZ_CP162096; pSAL22057-OXA, NZ_CP162097; pSAL22057-4, NZ_CP162098; pSAL22057-3, NZ_CP162099; pSAL22057-NDM, NZ_CP195667; pSAL22057-5, NZ_CP195668; and pSAL22057-6, NZ_CP195669.

## SUPPLEMENTAL MATERIAL

Fig. S1 spectrum.03207-24-s0001.tif Circular comparison between pSAL22057-NDM and other reported similar plasmids: pHNAHS65I-1, pNDM13-SR33, and psg_wt5. The circular map was generated using the BLAST Ring Image Generator (BRIG).

Fig. S2 spectrum.03207-24-s0002.tif Circular comparison between pSAL22057-OXA and other reported similar plasmids: pNDM33-1 (MN915011), pSJ_255 (CP011062), and pLH30-mcr1 (CM008265).

Table S1 spectrum.03207-24-s0003.doc Virulence-associated genes in SAL22057.

Table S2 spectrum.03207-24-s0004.doc Characteristics of all Salmonella included in comparative genomic analysis.

OPEN PEER REVIEW reviewer-comments.pdf An accounting of the reviewer comments and feedback.

ASM does not own the copyrights to Supplemental Material that may be linked to, or accessed through, an article. The authors have granted ASM a non-exclusive, world-wide license to publish the Supplemental Material files. Please contact the corresponding author directly for reuse.
