# Supplementary figures and images for: Carbapenem resistance mediated by blaNDM-13 in a highly drug-resistant Salmonella Stanley ST29 strain in China
Source: Microbiol Spectr. 2026 Jun 9;14(7):e03207-24. doi: 10.1128/spectrum.03207-24 (PMC13340070; doi:10.1128/spectrum.03207-24)

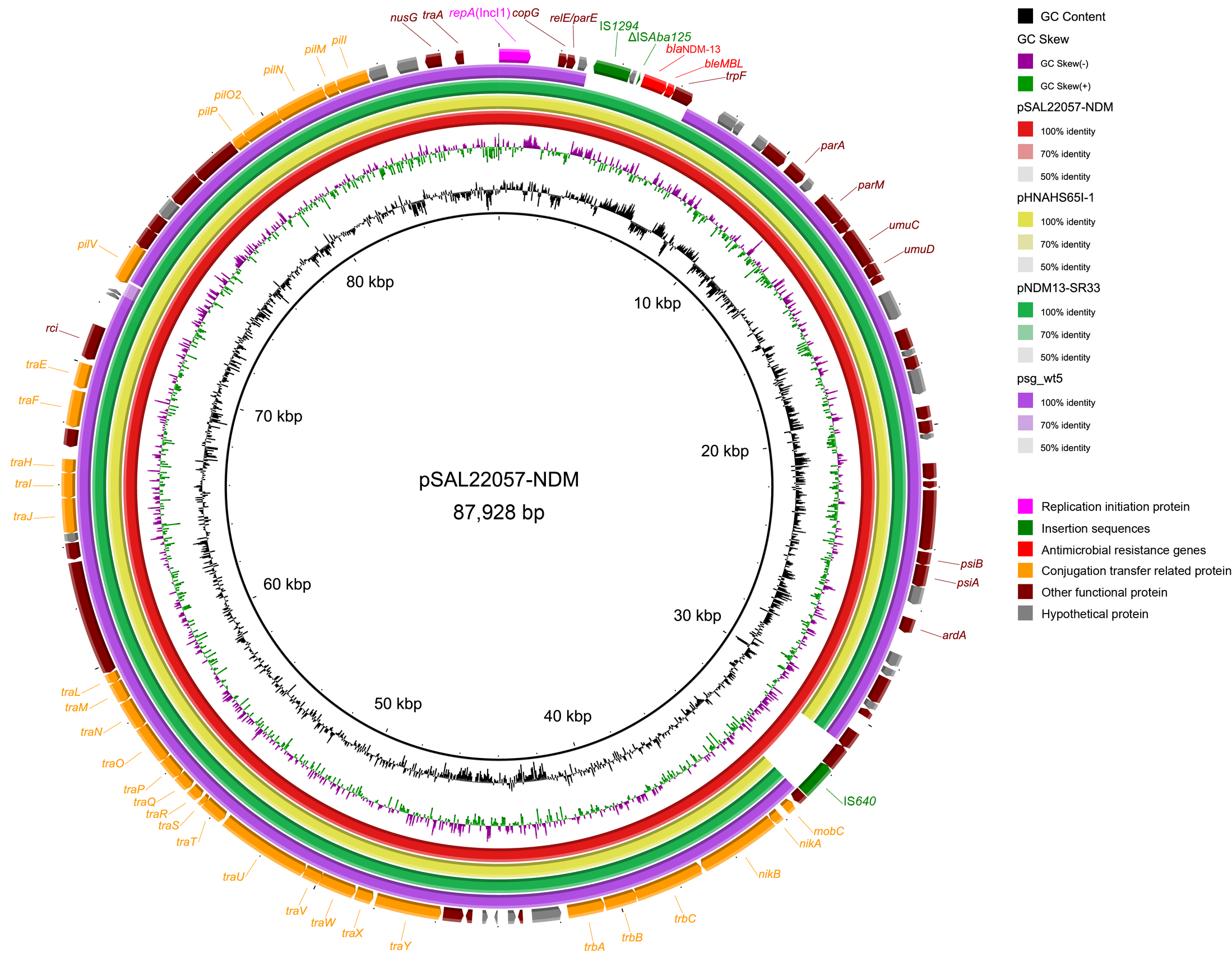

Supplement: Fig. S1 — Circular comparison between pSAL22057-NDM and other reported similar plasmids: pHNAHS65I-1, pNDM13-SR33, and psg_wt5. The circular map was generated using the BLAST Ring Image Generator (BRIG). [file spectrum.03207-24-s0001.tif]

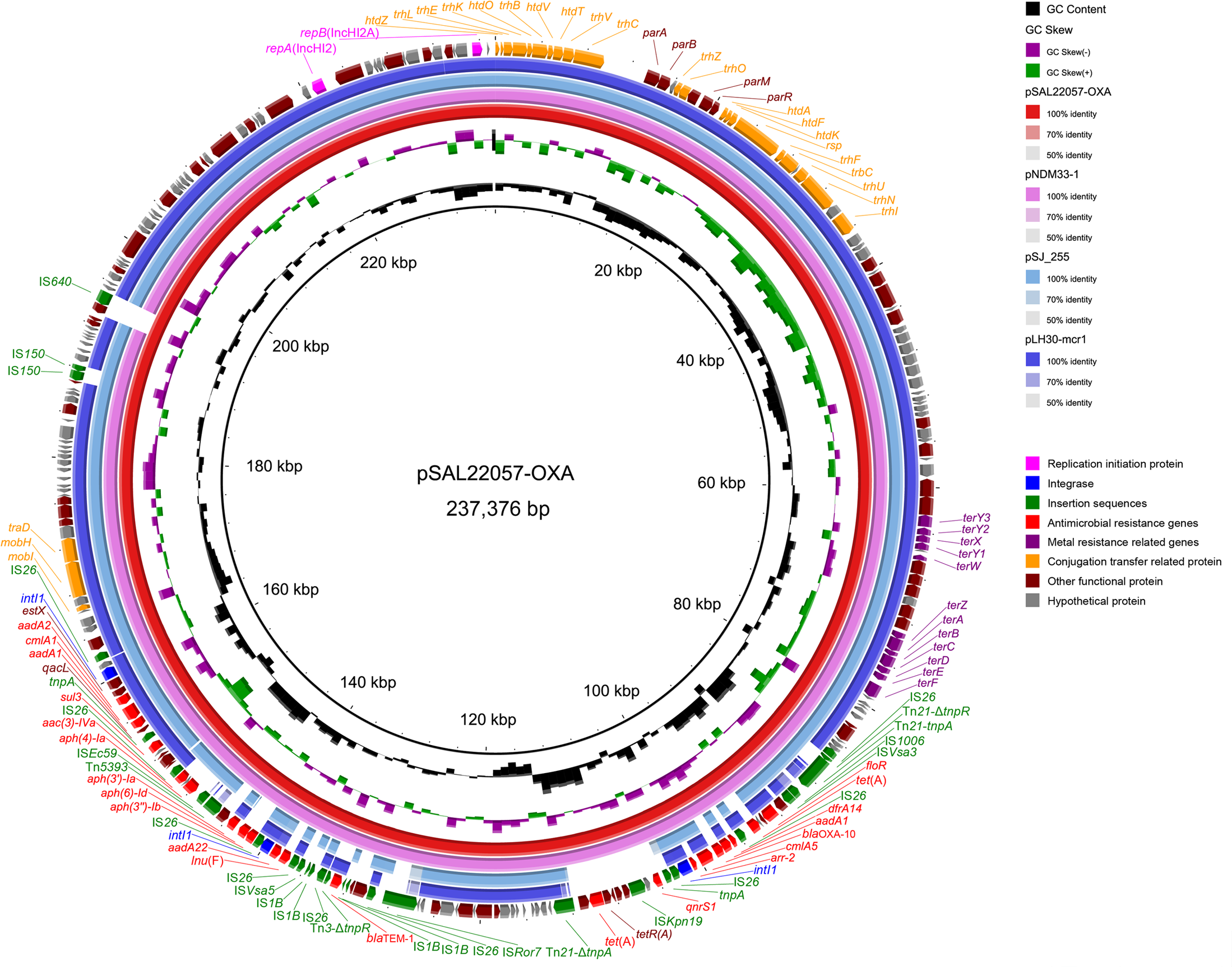

Supplement: Fig. S2 — Circular comparison between pSAL22057-OXA and other reported similar plasmids: pNDM33-1 (MN915011), pSJ_255 (CP011062), and pLH30-mcr1 (CM008265). [file spectrum.03207-24-s0002.tif]
