# Supplementary material for: Carbapenem resistance mediated by blaNDM-13 in a highly drug-resistant Salmonella Stanley ST29 strain in China
Source: Microbiol Spectr. 2026 Jun 9;14(7):e03207-24. doi: 10.1128/spectrum.03207-24 (PMC13340070; doi:10.1128/spectrum.03207-24)
Supplement: Table S1 — Virulence-associated genes in SAL22057. [file spectrum.03207-24-s0003.doc]

Supplementary Table 1 Virulence-associated genes in SAL22057

| **VF classes** | **Virulence factors** | **Genes** |
| --- | --- | --- |
| Fimbrial adherence determinants | Csg | *csgABCDEFG* |
|  | Lpf | *lpfABCDE* |
| Fimbrial | Fimbrial | *bcfABCDEFGH, fimACDFHIWYZ, safABCD, stbABCDE, stcABCD, stdABC, steABCDEF, stfACDEFG, sthABCDE, stiABCH* |
| Regulation | *phoPQ* | *phoPQ* |
|  | PmrAB | *PmrAB* |
| Secretion system | *Fur* | *Fur* |
|  | *RcsAB* | *rcsB* |
|  | *RpoS* | *rpoS* |
|  | TTSS (SPI-1 encode) | *AvrA, clpV, hilACD, iacP, iagB, invABCEFGHIJ, orgABC, pinB2, prgHIJK, sicAP, sifB, sipABCD, sopADE, spaOPXRS, sprB, sptP, sseJL, sspH2, steAC* |
| Regulation | TTSS (SPI-2 encode) | *ssaCDEFHIJKLMNOPQRSTUV, sscABC, sseABCDE, ssrAB* |
|  | TTSS effectors translocated via both systems | *slrP* |
|  | TTSS-1 translocated effectors | *avrA, sipABC, sopABD, sopE2, sptP* |
|  | TTSS-2 translocated effectors | *pipB2, sifA, sifB, spiC, sseFGIJL, sspH2* |
| Iron uptake | Aerobactin (Klebsiella) | *iucAD, iutA* |
| Motility  Other genes | Peritrichous flagella | *fljAB, flk, fliABDEFGHIJKLMNOPQRSTZY, flhCD, motAB, cheABMRWYZ, flhABE, flgABCDEFHIGJKLMN,* |
|  | - | *mig-14, mgtBC, misL, ratB, shdA, sinH* |
