# Supplementary material for: Carbapenem resistance mediated by blaNDM-13 in a highly drug-resistant Salmonella Stanley ST29 strain in China
Source: Microbiol Spectr. 2026 Jun 9;14(7):e03207-24. doi: 10.1128/spectrum.03207-24 (PMC13340070; doi:10.1128/spectrum.03207-24)
Supplement: Table S2 — Characteristics of all Salmonella included in comparative genomic analysis. [file spectrum.03207-24-s0004.doc]

Table S2 Characteristics of all *Salmonella* included in comparative genomic analysis.

| **Assembly** | **OXA-10/NDM-13** | **IncHI2** | **Genbank1** | **IncI1** | **Genbank2** | **ST** | **Serovar** | **Year** | **Location** | **Host** | **Replicon** |
| --- | --- | --- | --- | --- | --- | --- | --- | --- | --- | --- | --- |
| GCA_015697825.1 | OXA-10 | pS304_1 | CP061127.1 | - | - | 34 | 1,4,[5],12:i:- | 2015 | China:Hangzhou | Homo sapiens | IncHI2 |
| GCA_051997875.1 | OXA-10 | pCBUKPS070-1 | CP197164.1 | - | - | 34 | 1,4,[5],12:i:- | 2022 | South Korea | swine | IncHI2 |
| GCA_017372255.1 | OXA-10 | unnamed1 | CP071694.1 | - | - | 11 | Enteritidis | 2020 | China:WuHan | Homo sapiens | IncHI2, IncFIB(S), IncFII(S) |
| GCA_051027365.1 | OXA-10 | p.A | CP172411.1 | - | - | 155 | London | 2021 | China:Chongqing | Homo sapiens | IncHI2 |
| GCA_051999245.1 | OXA-10 | pCBUKPS057-1 | CP197156.1 | - | - | 34 | 1,4,[5],12:i:- | 2022 | South Korea | swine | IncHI2 |
| GCA_045344195.1 | OXA-10 | pZ1323SSL0059-2 | CP149151.1 | - | - | 34 | 1,4,[5],12:i:- | 2023 | South Korea | Pig | IncHI2 |
| GCA_051998935.1 | OXA-10 | pCBUKPS054-1 | CP197153.1 | - | - | 34 | 1,4,[5],12:i:- | 2022 | South Korea | swine | IncHI2 |
| GCA_037821945.1 | OXA-10 | pZ1323SSL0064-2 | CP149137.1 | - | - | 34 | 1,4,[5],12:i:- | 2023 | South Korea | Pig | IncHI2 |
| GCA_037828885.1 | OXA-10 | pZ1323SSL0051-2 | CP149176.1 | - | - | 34 | 1,4,[5],12:i:- | 2023 | South Korea | Pig | IncHI2 |
| GCA_045344205.1 | OXA-10 | pZ1323SSL0060-2 | CP149147.1 | - | - | 34 | 1,4,[5],12:i:- | 2023 | South Korea | Pig | IncHI2 |
| GCA_037823755.1 | OXA-10 | pZ1323SSL0062-2 | CP149143.1 | - | - | 34 | 1,4,[5],12:i:- | 2023 | South Korea | Pig | IncHI2 |
| GCA_037844905.1 | OXA-10 | pZ1323SSL0055-2 | CP149160.1 | - | - | 34 | 1,4,[5],12:i:- | 2023 | South Korea | Pig | IncHI2 |
| GCA_037825795.1 | OXA-10 | pZ1323SSL0054-2 | CP149164.1 | - | - | 34 | 1,4,[5],12:i:- | 2023 | South Korea | Pig | IncHI2 |
| GCA_030913615.1 | OXA-10 | pZ1323SSL0063-1 | CP133184.1 | - | - | 34 | 1,4,[5],12:i:- | 2023 | South Korea: Jeonbuk province | Pig | IncHI2 |
| GCA_037827835.1 | OXA-10 | pZ1323SSL0052-2 | CP149172.1 | - | - | 34 | 1,4,[5],12:i:- | 2023 | South Korea | Pig | IncHI2 |
| GCA_037844235.1 | OXA-10 | pZ1323SSL0061-2 | CP149288.1 | - | - | 34 | 1,4,[5],12:i:- | 2023 | South Korea | Pig | IncHI2 |
| GCA_037826765.1 | OXA-10 | pZ1323SSL0053-2 | CP149168.1 | - | - | 34 | 1,4,[5],12:i:- | 2023 | South Korea | Pig | IncHI2 |
| GCA_051997185.1 | OXA-10 | pCBUKPS053-1 | CP197147.1 | - | - | 34 | 1,4,[5],12:i:- | 2022 | South Korea | swine | IncHI2 |
| GCA_022557215.1 | NDM-13 | - | - | pNDM13-SR33 | CP092912.1 | 469 | Rissen | 2021 | China: Xiamen, Fujian | Homo sapiens | IncI1α |
| GCA_053048045.1 | NDM-13 | - | - | pST9343-1 | CP162126.1 | unknown | 1,4,[5],12:i:- | 2023 | China: zhuhai | Homo sapiens | IncI1α |
| GCA_040194395.1 | NDM-13 | - | - | pNBFE-164 | JBEFNU010000002.1 | 19 | Typhimurium | 2023 | China: Zhejiang | Homo sapiens | IncI1α |
| GCA_031036275.1 | - | - | - | p175-CMY-102k | CP065129.1 | 29 | Stanley | 2010 | China: Taiwan | Homo sapiens | IncI1α |
| GCA_014492535.1 | - | - | - | - | - | 29 | Stanley | 2018 | China: Hangzhou | Homo sapiens | - |
| GCA_014492425.1 | - | - | - | - | - | 29 | Stanley | 2018 | China: Hangzhou | Homo sapiens | - |
| GCA_014492325.1 | - | - | - | - | WPLN01000021.1 | 29 | Stanley | 2018 | China: Hangzhou | Homo sapiens | IncI1α |
| GCA_043851255.1 | - | - | - | - | JBGSEM010000008.1 | 29 | Stanley | 2019 | China: Hubei | Homo sapiens | IncI1α |
| GCA_021545805.1 | - | pSal661_2 | CP067079.1 | - | - | 29 | Stanley | 2019 | China: Guangdong | Homo sapiens | IncHI2 |
| GCA_033355495.1 | - | - | JAOUXO010000002.1 | - | - | 29 | Stanley | 2020 | China: Shenzhen | Homo sapiens | IncHI2 |
| GCA_033355995.1 | - | - | JAOUYD010000016.1/20.1 | - | - | 29 | Stanley | 2020 | China: Shenzhen | Homo sapiens | IncHI2 |
| GCA_043849585.1 | - | - | - | - | - | 29 | Stanley | 2020 | China: Fujian | Homo sapiens | - |
| GCA_041060305.1 | - | - | JBFUXW010000016.1 | - | - | 29 | Stanley | 2023 | China: Shanghai | Homo sapiens | IncHI2 |
| GCA_050598075.2 | OXA-10+NDM-13 | pSAL22057-OXA | CP162097.2 | pSAL22057-NDM | CP195667.1 | 29 | Stanley | 2022 | China:Zhengzhou | Homo sapiens | IncI1α, IncHI2 |
