# Supplementary material for: Carbapenem resistance mediated by blaNDM-13 in a highly drug-resistant Salmonella Stanley ST29 strain in China
Source: Microbiol Spectr. 2026 Jun 9;14(7):e03207-24. doi: 10.1128/spectrum.03207-24 (PMC13340070; doi:10.1128/spectrum.03207-24)
Supplement: Reviewer comments [file reviewer-comments.pdf]

AMERICAN SOCIETY FOR MICROBIOLOGY | Microbiology Spectrum

Carbapenem Resistance Mediated by *bla*$_{NDM-13}$ in a Highly Drug-Resistant *Salmonella* Stanley ST29 strain in China

Juanjuan Zhou, Junmei Yang, Ke Li, Hongna Shi, Kaijie Gao, Peipei Zhao, Lu Xu, Dongyu Zhang, and Minghui Zhen

Corresponding Author(s): Juanjuan Zhou, Children's Hospital Affiliated to Zhengzhou University

Review Timeline:

Submission Date:	December 10, 2024
Editorial Decision:	April 22, 2025
Revision Received:	July 22, 2025
Editorial Decision:	September 29, 2025
Revision Received:	December 12, 2025
Editorial Decision:	February 1, 2026
Revision Received:	March 23, 2026
Accepted:	April 22, 2026

Editor: Jian Peng

Reviewer(s): Disclosure of reviewer identity is with reference to reviewer comments included in decision letter(s). The following individuals involved in review of your submission have agreed to reveal their identity: Jiaoyu Deng (Reviewer #2); Beiwen Zheng (Reviewer #4)

Transaction Report:

(Note: With the exception of the correction of typographical or spelling errors that could be a source of ambiguity, letters and reports are not edited. The original formatting of letters and referee reports may not be reflected in this compilation.)

DOI: https://doi.org/10.1128/spectrum.03207-24

Re: Spectrum03207-24 (Emergence of NDM-13 and OXA-10 Co-producing Salmonella Stanley ST29 clinical strain in China)

Dear Mrs. Juanjuan Zhou:

Thank you for the privilege of reviewing your work. Below you will find my comments, instructions from the Spectrum editorial office, and the reviewer comments.

Please return the manuscript within 60 days; if you cannot complete the modification within this time period, please contact me. If you do not wish to modify the manuscript and prefer to submit it to another journal, notify me immediately so that the manuscript may be formally withdrawn from consideration by Spectrum.

Revision Guidelines
To submit your modified manuscript, log into the submission site at https://spectrum.msubmit.net/cgi-bin/main.plex. Go to Author Tasks and click the appropriate manuscript title to begin. The information you entered when you first submitted the paper will be displayed; update this as necessary. Note the following requirements:

• Upload point-by-point responses to the issues raised by the reviewers in a file named "Response to Reviewers," NOT in your cover letter.
• Upload a compare copy of the manuscript (without figures) as a "Marked-Up Manuscript" file.
• Upload a clean .DOC/.DOCX version of the revised manuscript and remove the previous version.
• Each figure must be uploaded as a separate, editable, high-resolution file (TIFF or EPS preferred), and any multipanel figures must be assembled into one file.
• Any supplemental material intended for posting by ASM should be uploaded with their legends separate from the main manuscript. You can combine all supplemental material into one file (preferred) or split it into a maximum of 10 files with all associated legends included.

For complete guidelines on revision requirements, see our Submission and Review Process webpage. Submission of a paper that does not conform to guidelines may delay acceptance of your manuscript.

Data availability: ASM policy requires that data be available to the public upon online posting of the article, so please verify all links to sequence records, if present, and make sure that each number retrieves the full record of the data. If a new accession number is not linked or a link is broken, provide Spectrum production staff with the correct URL for the record. If the accession numbers for new data are not publicly accessible before the expected online posting of the article, publication may be delayed; please contact production staff (Spectrum@asmusa.org) immediately with the expected release date.

Publication Fees: For information on publication fees and which article types are subject to charges, visit our website. If your manuscript is accepted for publication and any fees apply, you will be contacted separately about payment during the production process; please follow the instructions in that e-mail. Arrangements for payment must be made before your article is published.

ASM Membership: Corresponding authors may join or renew ASM membership to obtain discounts on publication fees. Need to upgrade your membership level? Please contact Customer Service at Service@asmusa.org.

The ASM Journals program strives for constant improvement in our submission and publication process. Please tell us how we can improve your experience by taking this quick Author Survey.

Thank you for submitting your paper to Spectrum.

Sincerely,
Jian Peng
Editor
Microbiology Spectrum

Reviewer #1 (Comments for the Author):

This manuscript describes analysis of a Salmonella isolate that carries the blaNDM-13 gene on a large plasmid with both HI and I1alpha replicons. blaOXA-10 is also mentioned in several places but as this gene is not considered to encode a carbapenemase the reason for focusing on it is unclear (many other AMR genes are also present). The descriptions of pSAL22057-NDM-13 are not easy to follow and do not really explain how it might have arisen. Experimental proof that

pSAL22057-NDM-13 exists a conintegrate or hybrid plasmid and that this is not an assembly artefact, is really needed, and determining whether this structure stably persists on conjugation and beyond would add to the manuscript.

1) pSAL22057-NDM-13
The descriptions of various aspects of pSAL22057-NDM-13, which make up a substantiation portion of the manuscript, are hard to follow.
Lines 112-3 - is it possible that the two copies of the HI2 replicon could be an assembly issue? Also, IncI plasmids have a shufflon that may not be correctly assembled, even from long read data (see PMID: 28424528). This needs to be looked at and may explain some differences from other IncI1 plasmids.
Lines 115-6 - it is usually not possible to be sure exactly which plasmids another plasmid could have been derived from, so this needs to be written differently. i.e. that it appears to be a cointegrate (two complete plasmids joined) or hybrid (parts of two different plasmids) of HI2-like and I1alpha-like plasmids. And why "resembles? If a plasmid has a specific replicon then it is normally classed as that plasmid type. Also "the IncI1alpha plasmid" does not make sense here, as many different IncI1alpha plasmids have been identified.
Lines 118-20 - this is unclear. What is meant by "MGE areas" and how does this differ from "as well as MGE" later in the sentence. The blaOXA-10 gene cassette is presumably in an integron?
Line 124 - position 1 presumably refers to CP162097, but this should be stated. Why were these positions decided as being the ends of "Region 1"? Position 1 is slightly downstream of ardA.
Line 127-8 - meaning unclear.
Lines 128-9 - the information about IS640 isn't relevant to this manuscript. It would be more useful to look for flanking direct repeats, as these would give evidence of a simple insertion, and state if these are present (also Lines 135-6).
Lines 130-2 - again, how were the ends of region 2 decided on?
Line 138 - this region needs to be described better. "variable" in comparison with what?
Line 137 - how is it known that this plasmid is "slime-mold resistant"? A citation is needed for this.
Line 141-2 - what is meant by "integrated substructures"? What is meant by "MCR-1" here? This is not clear.
Lines 144-6 - the meaning here is unclear.
Lines 147-8 - this could just say "Transconjugants of E. coli C600 carrying blaNDM-13 were obtained".

2) Lines 151-6, 227-238, 346-54
Doing phylogenetic analysis of plasmids in this way does not make sense - they are too recombinational and gain and lose large segments, which cannot be accommodated in a tree. This would only be useful for e.g. comparing the backbone SNPs of closely related plasmids of similar length that belong to the same Inc type. It is entirely expected that these sets of related plasmids would group by Inc type.
Line 234 - what are "type I" and "type 2" plasmids.

3)Methods
Lines 303-4 - more details are needed. Liquid or solid mating, what media, what donor:recipient ration, time or conjugation? Or cite a suitable reference.
Lines 309-11 - as blaNDM-13 is in the IncI1aplpha part of pSAL22057-NDM-13 and blaOXA-10 is in the IncHI2 part, finding these genes does not indicate whether or not the two plasmids have stayed together in the transconjugant. This really needs to be looked at further and if the transconjugant does contain the combined plasmid, how stable is it? This could be looked at by passaging with/without antibiotics.
Line 314 - this needs a reference

4) Discussion
This is too long should not simply repeat Results.
Line 165 - transfer to a single E. coli only was examined here.
Lines 165-6 - blaOXA-1 is not a "significant carbapenemase gene". How do you know that the plasmid is "highly well-adapted" and this study only mentions a single plasmid.
Line 171 - which slaughterhouse?
Line 171-2 - are all ST29 S. Stanley?
Lines 172-3 - can this really be said based on one isolate from each location?
Lines 180-84 - this is not relevant, as blaOXA-10 is not a considered a carbapenemase gene.
Lines 184-6 - it seems unlikely that this was the first isolate with both of these genes.
Lines 186-7 - OXA-1 has been described as having "weak carbapenemase activity". Ref 18 appears to be about OXA-10 variants that give increased ceftolozane/tazobactam and CAZ/avibactam resistance in Pseudonomas, but in fact have reduced activity against carbapenems.
Line 187-8 - I may be misunderstanding this statement, but I do not believe ut. A BLAST Search shows that there are many sequences in GenBank with blaOXA-10 from Enterobacterales and many of these would likely have associated publications.
Lines 190-1 - why does OXA-10 "warrant specific attention"?
Lines 192-226 - most of this information repeats Results or should be in Results, not Discussion, and is hard to follow.
Line 197-8 - the contents of IS640 are not relevant here.
Lines 198-9 do not make sense. What is "exogeneous insertion"?
Line 201 - what is meant by "externally inserted"?

Lines 208-10 - again, IS640 is not really relevant.
Line 210 - does pSAL22057-NDM-13 have full conjugation regions of both HI2 and I1 plasmids?
Line 214 - what suggest this? Are there references for the related plasmids being conjugative that could be cited?
Lines 217-9 - a recent paper has suggested a different function for "ter" genes (PMID: 33930099), so this needs checking
Lines 220-21 - this wording is unclear - meaning that regions 1 and 2 didn't have any segments in common?
Lines 225-6 - this is not a possible explanation - both copies of IS640 appear to be simply insertions, with flanking DR of characteristic 4 bp in length and they are not at the junctions of regions 1 and 2 claimed to be derived from the different plasmid types.
Line 239 - trfF is found downstream of blaNDM in many sequences - the region around this gene is highly conserved.
Lines 242,247-52 - this should refer to IS1294, not "the IS1294 transposase" that it contains. IS1294 is member of the IS91 family of rolling circle elements that are known to be able to capture and move adjacent sequences. This should be looked at properly to see if it could help to explain generation of pSAL22057-NDM-13 but see comments above about possible assembly issues.
Lines 243, 246 - referring to these genes by such complicated names is not helpful.
Lines 253- - this information should be given in Results and more details of the integrons and which cassettes they contain is needed. Region 2 will not include proteins, only the genes encoding them.
Line 256 - what is meant by "typical E. coli plasmid regions"?
Lines 257-66 - IS15 is a variant of IS26 (see PMID: 34015086) and should be named as such. The wording "...insertion sequences are derived from the IS15 transpoase tnp2" does not make sense - an IS is not derived from a gene - and where does the name tnp2 come from? The authors should look at recent papers and reviews on IS26 by RM Hall and colleagues and M. Chandler and colleagues to understand about IS26-mediated TU and "pseudocompound" transposons and how the resistance region in Region 2 could have arisen.
Line 262 - "TnAs3 tnpR" matches Tn21, which is a well-known Tn that carries an integron, so it would be better to use this name (also on Figures).
Lines 267-70 - which plasmid has the inverted region with blaNDM-5 that is mentioned? Is there any clue about how this inversion might have occurred? Why would blaNDM-5 be acquired and by what?

5) Other scientific points
Line 43 - "other E. coli species" does not make sense - E. coli is a species. Do you mean other strains? If so, it seems that conjugation of pSAL22057-NDM-13 to EC600 only was tested.
Line 46-7 - does identification of a single isolate really make monitoring of it "urgent" and how could it be "controlled"?
Line 69 - this is out of date. The NCBI Reference Gene Catalog now lists 75 NDM variants (https://www.ncbi.nlm.nih.gov/pathogens/refgene/#blaNDM)
Lines 71-2 - a quick BLASTn search with the blaNDM-13 sequence from the Reference Gene Catalog identifies 10 sequences with this gene, all from Enterobacterales, not including pSAL22057-NDM-13.
Lines 96-8 - I couldn't find blaTEM-60 on the chromosome and only found blaTEM-1b, not blaTEM-95, on pSAL22057-NDM-13 and these genes didn't come up in a ResFinder analysis - this needs checking.
Lines 101-2 - this is the wrong place for this information - it should be given before WGS is discussed, especially if this is how the Stanley serotype was first identified.
Lines 103-10 - this section really doesn't say much. Could anything more be made of it? For example, is anything much known about SPIs in S. Stanley?
Lines 298-9 - would these primers detect all variants of blaIMP and blaVIM? The blaOXA group in particular is very broad and includes many subgroups - which types would be detected by the primers used? blaOXA-48-like? Ref 23 needs to be checked and this information stated correctly.
Line 311 - what is meant by "conjugation elements"?
Lines 371-4 - the sequences in GenBank are not annotated. It would be helpful to add annotations.

6) Tables and Figures
Table 1
Title - strictly speaking, it is the antibiotics that have the MICs, not the isolates, should "transmant" be "transconjugant" and "recipi" at the end is missing letters. The table might be better presented with the rows and columns transposed, i.e. antibiotic names down the side and a column for each strain. Why only mention beta-lactamase genes when MICs are also given for other antibiotic types?
Figure 1
The text on the diagram is hard to read. This figure might be better as supplementary.
Line 451 - this should be "dashed" not "dash", but the lines don't look dashed.
Figure 2
The text on the diagrams is hard to read. A scale of % identity is only shown on Part B and different colours are used in other parts - it would be better to be consistent across figures and there should be something to explain what all of the coloured shading means.
Part A - "backbone", rather than "skeleton" is normally used for the parts of plasmids carrying genes encoding plasmid functions in which insertions are found.
Part B - the purpose of comparing these sequences is not really clear from the text.
Part C - in pSAL22057-NDM-13, the first IS26 (labelled as "IS15-tnp2") starts at 173167 and the last one ends at 251001, so it is

not clear why the positions stated here are 173100-251000? This also needs checking for the other sequences shown.
Figure 3
See comments on the tree part above.

7) Supplementary
Table 1
This is not enough information to merit a table - all of the information is already stated in text, or could be, and it is organized in an odd way. All parts of resistance gene names should be in italics, except bla gene subscripts, and QnrS1 should be qnrS1.
See comment above on blaTEM in chromosome.
Table S2
All letters in gene names should be italicized. The first letters of protein names should be upper case.

8) Minor/formatting/English etc
Salmonella serovars should be in plain text, not italics so "Stanley" needs to be changed throughout.
Line 34-5 - wording does not make sense - "genomic characterization" is not "examined".
Line 37 - should be "(MIC 32 ug/ml)... (MIC 16 ug/ml)".
Line 38 - should be "belongs to".
Lines 40-2 - suggest "analysis identified blaNDM-13 on a conjugative hybrid plasmid with HI2 and I1alpha replicons", but using an alpha symbol.
Lines 53-4 - wording does not make sense.
Lines 57-60 - the gene names should be used here (i.e. blaKPC etc, correctly formatted) and there is no need to use the full names - these are all well-known genes - and presumably "or blaOXA-48-like genes" is meant?
Line 63 - this wording is quite awkward.
Line 67 - "lactamase" not "lactamases".
Line 78 - heading should be "Results".
Lines 80, 285 - presumably "one and a half year old"? "18 month old" might be better.
Line 83 - if all antibiotics are listed then "including" is incorrect here. If some are not listed then they should be added, but this list might not be needed here and could just be given in Methods. The normal abbreviation for cefotaxime is CTX, not CXT (also Line 293).
Line 91 - "detection of"
Lines 93-4 - suggest "to have a 4,847,514 bp chromosome and". Why were the plasmids numbered in this order? It would probably have been more logical to do this e.g. in order of decreasing size.
Line 96 - blaTEM genes encode beta-lactam resistance, not beta-lactams.
Line 97 - remove "The" from start of sentence.
Line 106 - should be "harbors".
Line 112 - "simultaneously" is not needed.
Line 113 - delete "of".
Line 147 - delete "the" before blaNDM-13.
Line 158 - the gene name is blaNDM-1. The wording here could be simplified e.g. "The first example of blaNDM-1 in Salmonella was documented..."
Line 160 - what is meant by "genetic backgrounds" here?
Line 162 - again, this should refer to the blaNDM gene, and is being found in a single isolate really "emerging"? (also applies to the manuscript title).
Lines 235-6 - "conjugative transfer"
Lines 276-7 - "within the SAL22057 strain" is not needed.
Line 285 - it was not known at this stage that SAL22037 is S. Stanley?
Line 286 - presumably this was species identification?
Lines 292-3 - the abbreviation for ceftazidime was given on Line 84 and MICs are not "performed".
Line 295-6 - lowercase c for carbapenemase and a reference for this method is needed.
Line 299 - "PCR positive products" does not make sense.
Line 302 - this section does not mention plasmid sequencing.
Lines 303-4 - which plasmid?
Line 305 - "transconjugants" not "conjugants"
Line 307 - "identified as E. coli" and the MALDI-TOF details have already been given on Line 287-8.
Lines 322-3 - wording does not make sense - words missing?
Lines 337-42 - this reads more like it should be in Results.
Lines 347-9 - the wording here does not make sense and seem like more like Results than Methods.
References - change Title Case to Sentence case (e.g. Refs 3, 4,9, 10) and check that species and gene names etc. are correctly formatted. The citation for Ref2 is in the wrong format.

Reviewer #2 (Comments for the Author):

In this manuscript, Zhou et al. isolated and characterized a NDM-13 and OXA-10 co-producing Salmonella Stanley ST29 clinical

strain, with the blaNDM-13 and blaOXA-10 genes on a newfound transmissible conjugative hybrid plasmid pSAL22057-NDM13, highlighting the urgent necessity for monitoring and controlling of clinical strains co-producing NDM-13 and OXA-10. The study is of clinical importance, and my concerns are minus.
1) Language editing is necessary. For example, line 36 "testing indicated" should be as "testing showed", line 37 "(MICs of 32μg/ml)" should be as "(MICs of 32 μg/ml), Lines 77, "strain isolated a child with 77 acute diarrhea and fever" should be as "strain isolated from a child with 77 acute diarrhea and fever".
2) So far, any signs of existence of the newly identified plasmid in other clinical strains except for SAL22057?

Reviewer #3 (Comments for the Author):

This study analyses a strain of Salmonella Stanley ST29 that produces both NDM-13 and OXA-10 enzymes, which is relatively rare and informative for understanding resistance in Salmonella spp. There are several comments for reference.
1. Line 96-98: How to explain the presence of TEM genes on both chromosome (blaTEM-60) and plasmid (blaTEM-1 and blaTEM-95)? Is it because the threshold was set too low during sequencing sequence analysis and not filtered effectively resulting in false positive for blaTEM resistance genes?
2. Line 92-110: '2.2' and '2.3' can be combined into one paragraph.
3. Line 111-156: Paragraphs '2.4' and '2.5' could be combined into one paragraph.
4. although there is a description of 'Conjugation Assay' in the Methodology section, there is no relevant information in the Results section, it is recommended to describe it separately in the Results section or combine it with the results of the antimicrobial susceptibility testing.
5. The description in the Discussion section should be more concise, and part of the description should be repeated with the results.
6. It is recommended to add information about the medical history, the antimicrobial drugs used to treat the infection, and the prognosis.
7. blaTEM-60 is missing from the resistance genes in Table 1, and blaTEM-60 is an extended-spectrum enzyme that can hydrolyse aztreonam. However, the results of the susceptibility testing show that the bacteria are susceptible to aztreonam, how can this be explained?

Responses to reviewer #1
Reviewer #1 (Comments for the Author):

1. This manuscript describes analysis of a Salmonella isolate that carries the blaNDM-13 gene on a large plasmid with both HI and I1alpha replicons. blaOXA-10 is also mentioned in several places but as this gene is not considered to encode a carbapenemase the reason for focusing on it is unclear (many other AMR genes are also present). The descriptions of pSAL22057-NDM-13 are not easy to follow and do not really explain how it might have arisen. Experimental proof that pSAL22057-NDM-13 exists a conintegrate or hybrid plasmid and that this is not an assembly artefact, is really needed, and determining whether this structure stably persists on conjugation and beyond would add to the manuscript.

Authors' response: We deeply appreciate your interest in our article and the highly constructive feedback you provided. Based on this feedback, our team has undertaken a thorough re-examination of our data, analysis, and content, followed by comprehensive revisions. We have diligently addressed the issues, as outlined below.

2. blaOXA-10 is also mentioned in several places but as this gene is not considered to encode a carbapenemase the reason for focusing on it is unclear (many other AMR genes are also present).

Authors' response: Thank you for your question. When expressed in *Acinetobacter baumannii* ATCC 17978, OXA-10 conferred high-level resistance to carbapenems, with MIC values for meropenem, ertapenem, and doripenem comparable to those of recognized carbapenemases like OXA-48 and higher than OXA-58. For example, the MICs of meropenem, ertapenem, and doripenem were 16 μg/ml, 128 μg/ml, and 16

μg/ml, respectively, indicating clinical resistance levels. Although OXA-10 has low hydrolysis efficiency for carbapenems, it exhibits high substrate affinity (nanomolar dissociation constants), consistent with carbapenemase characteristics. In *Acinetobacter baumannii* (with low membrane permeability and efflux pumps), it showed significant resistance, a hallmark of carbapenemase. OXA-10 was expressed 25-fold more highly in *Acinetobacter baumannii* than OXA-48. High expression levels offset lower catalytic efficiency, leading to equivalent resistance. In summary, OXA-10 has both carbapenem-hydrolyzing ability and clinical resistance phenotypes, qualifying it as a carbapenemase[1]. OXA-10 and its variants (e.g., OXA-655) exhibit carbapenem-hydrolyzing activity, host-dependent clinical resistance, and evolutionary plasticity, meeting the criteria for classification as carbapenemases[2]. Thus, OXA-10 showed to be weak carbapenemases. We added this part of the content in the revised manuscript (line 86-90).

1) Antunes NT et al. Class D β-lactamases: are they all carbapenemases? Antimicrob Agents Chemother 58, 2119-25(2014).

2) Kotsakis SD et al. Characterization of the First OXA-10 Natural Variant with Increased Carbapenemase Activity. Antimicrob Agents Chemother. 63, e01817-18(2018).

3. The descriptions of pSAL22057-NDM-13 are not easy to follow and do not really explain how it might have arisen. Experimental proof that pSAL22057-NDM-13 exists a conintegrate or hybrid plasmid and that this is not an assembly artefact, is really needed, and determining whether this structure stably persists on conjugation

and beyond would add to the manuscript.

Authors' response: Thank you for your question. During final validation for resubmission, we identified a critical assembly error in plasmid pSAL22057-NDM13. pSAL22057-NDM13 was erroneously constructed by the incorrect ligation of two parental plasmids (pSAL22057-NDM and pSAL22057-OXA), which deviated from the intended sequence. Strain SAL22057 was found to harbor a 4, 847,759 chromosome (52.2% G+C) and six plasmids named pSAL22057-OXA (2376, 376bp, carrying *bla*$_{OXA-10}$), pSAL22057-3 (93, 293bp), pSAL22057-NDM (87, 928bp, carrying *bla*$_{NDM-13}$), pSAL22057-4 (6, 477bp), pSAL22057-5 (5, 423bp) and pSAL22057-6 (4, 583bp) (See as section 2.2) (line 125-128).

The genome assembly method is as follows: "Long reads were independently assembled using Canu (v2.2)[1] (min Read Length=1000; corrected Error Rate=0.01; min Overlap Length=500; cor Out Coverage=50) and Flye (v2.8.3) (default parameters). QC-passed short reads were assembled with SOAPdenovo (v2.04)[2] (k-mer: 69–127; optimally assembled) and gaps closed using GapCloser (v1.12). Assemblies were evaluated by self-comparison (removing redundant contigs) and cross-comparison of Canu/Flye results (confirming replicon structure and selecting the most contiguous assembly). Putative circular replicons were identified via terminal fragment BLAST analysis. Short reads were mapped to long-read assemblies to detect potential small-plasmid loss and assess accuracy/completeness. Final replicons were polished with Pilon (v1.24)[3] using Illumina data. Uniform read coverage across all replicons validated assembly completeness and accuracy at the

sequencing level." We have revised Section 5.5 to enhance the clarity of the genome assembly methodology (line 294-305).

1） Koren S, et al. Canu: scalable and accurate long-read assembly via adaptivek-mer weighting and repeat separation. *Genome Research* **27**, 722-36 (2017).

2） Li R, et al. SOAP: short oligonucleotide alignment program [J]. *Bioinformatics* **24**, 713-4 (2008).

3） Wang J, et al. Pilon: An Integrated Tool for Comprehensive Microbial Variant Detection and Genome Assembly Improvement. *PLoS ONE* **9**, e112963 (2014).

4. pSAL22057-NDM-13

The descriptions of various aspects of pSAL22057-NDM-13, which make up a substantiation portion of the manuscript, are hard to follow.

(1) Lines 112-3 - is it possible that the two copies of the HI2 replicon could be an assembly issue? Also, IncI plasmids have a shufflon that may not be correctly assembled, even from long read data (see PMID: 28424528). This needs to be looked at and may explain some differences from other IncI1 plasmids.

Authors' response: We appreciate the reviewer's inquiry regarding the potential assembly artifact of the two HI2 replicon copies. To address this concern, we rigorously validated the genome assembly. The genome assembly method is detailed in Section 5.4. Re-assembly revealed that strain SAL22057 was found to harbor one chromosome and six plasmids, characterized as follows: pSAL22057-NDM carries an IncI1I*α* replicon replicon, pSAL22057-OXA possesses an IncHI2/IncHI2A, pSAL22057-3 belongs to the IncY incompatibility group, pSAL22057-4 corresponds

to IncQ1, pSAL22057-5 is typed as Col440I, and pSAL22057-6 is classified as ColRNAI. We have revised Section 2.2 (line 129-133).

(2) Lines 115-6 - it is usually not possible to be sure exactly which plasmids another plasmid could have been derived from, so this needs to be written differently. i.e. that it appears to be a cointegrate (two complete plasmids joined) or hybrid (parts of two different plasmids) of HI2-like and I1alpha-like plasmids. And why "resembles? If a plasmid has a specific replicon, then it is normally classed as that plasmid type. Also "the IncI1alpha plasmid" does not make sense here, as many different IncI1alpha plasmids have been identified.

Authors' response: Thank you for the valuable suggestion. The description of plasmid replicon types has been revised in the manuscript according to the above comments. The phrase 'the IncI1alpha plasmid' incorrectly implies a single, specific plasmid, whereas IncI-1α refers to a broad plasmid incompatibility group encompassing many variants. We have revised the text to clarify this distinction and corrected 'alpha' to the Greek symbol 'α' for scientific accuracy. (line 40, 130).

(3) Lines 118-20 - this is unclear. What is meant by "MGE areas" and how does this differ from "as well as MGE" later in the sentence. The blaOXA-10 gene cassette is presumably in an integron?

Authors' response: We appreciate the reviewer's careful critique and agree that original phrasing lacked clarity. According to the revised sequencing results, the phrases "MGE areas" and "as well as MGE" are no longer present. As correctly inferred, the *bla*$_{OXA-10}$ gene cassette is indeed embedded within an integron. This has

now been explicitly stated in the revised manuscript (line 176-182).

(4) Line 124 - position 1 presumably refers to CP162097, but this should be stated. Why were these positions decided as being the ends of "Region 1"? Position 1 is slightly downstream of ardA.

Authors' response: Thank you for the valuable suggestion. We have added CP numbers to the plasmids mentioned (line 153, 157 and 171). According to the revised sequencing results, the phrases "Region 1" is no longer present.

(5) Line 127-8 - meaning unclear.

Authors' response: Thank you for the valuable suggestion. The corresponding sentence has been rephrased to make it clearer (line 159-163).

(6) Lines 128-9 - the information about IS640 isn't relevant to this manuscript. It would be more useful to look for flanking direct repeats, as these would give evidence of a simple insertion, and state if these are present (also Lines 135-6).

Authors' response: Thanks for the comment. We have removed the information about IS*640* in the revised manuscript. We have identified the inverse and direct repeats within in the region of *bla*$_{NDM-13}$ (pSAL22057-NDM) and *bla*$_{OXA-10}$ (pSAL22057-OXA), and these have been annotated in the updated Figure 1B/2B. For further information, please consult Table S3, S4. IS*26* features two flanking 8-base

Table S2 The repeat sequences of the region of *bla*$_{NDM-13}$ in pSAL22057-NDM

**MGEs**	**IRs**	**Start**	**End**	**Sequence (5'→3')**
IS*Aba125*	IRR	4613	4662	GGCGAATTCAAACATGAGGTGCGACAGTTTCAAAAGCCATATGATAATCA
IS*1294*	oriIS	4512	4611	GAACCACGGCAGCCTGGCGGCTGCAAATTGAAAACCGCCCCGGTTCGTCC TGTTGTTGCCGCACGCCCCGCAGAATATGTATGCTCTGAGGACCTGTTTT
	terIS	2924	3023	ACAGAGAGGGAAACCACCGGAGTGGTGGGAAAAGCAGAAAGGCTCACGT GGCATAAGGCGTGGACAATGGTATCGACTGGTTTTTCAATTTCCTATACGT

Table S3The repeat sequences of the region of *bla*$_{OXA-10}$ in pSAL22057-OXA

MGEs	IRs	Start	End	Sequence (5'→3')
IS*26*	IRL	96183	96232	GGCACTGTTGCAAATAGTCGGTGGTGATAAACTTATCATCCCCTTTTGCT
	IRL	102818	102867	GGCACTGTTGCAAATAGTCGGTGGTGATAAACTTATCATCCCCTTTTGCT
	IRR	95413	95462	GGCACTGTTGCAAAGTTAGCGATGAGGCAGCCTTTTGTCTTATTCAAAGG
	IRR	102048	102097	GGCACTGTTGCAAAGTTAGCGATGAGGCAGCCTTTTGTCTTATTCAAAGG
	DR	96234	96241	TCGCAGGA
	DR	95405	95412	CTTTTTTG

-pair direct repeats (DRs): TCGCAGGA is adjacent to the left inverted repeat (IRL), and CTTTTTTG is adjacent to the right inverted repeat (IRR).

(7) Lines 130-2 - again, how were the ends of region 2 decided on?

Authors' response: Thank you for the valuable suggestion. Based on the re-assembly results, we characterized plasmid pSAL22057-NDM and pSAL22057-OXA (section 2.3 and 2.4). We have removed the information about region2 in the revised manuscript.

(8) Line 138 - this region needs to be described better. "variable" in comparison with what?

Authors' response: Thanks for your suggestion. Based on the re-assembly results, we have deleted it.

(9) Line 137 - how is it known that this plasmid is "slime-mold resistant"? A citation is needed for this.

Authors' response: Thanks for your suggestion. Since this part is irrelevant to the theme of the article, we have deleted it.

(10) Line 141-2 - what is meant by "integrated substructures"? What is meant by "MCR-1" here? This is not clear.

Authors' response: Thank you for the valuable suggestion. Based on the re-assembly results, we rephrased this section (section 2.4).

(11) Lines 144-6 - the meaning here is unclear.

Authors' response: Thanks for your suggestion. Based on the re-assembly results, we have deleted it.

(12) Lines 147-8 - this could just say "Transconjugants of E. coli C600 carrying blaNDM-13 were obtained".

Authors' response: Thank you for your suggestion. We rephrased this as "Conjugation experiments confirmed that these transconjugants carried $\textit{bla}_{\text{NDM-13}}$ but not $\textit{bla}_{\text{OXA-10}}$..." (line 120-121)

5. Lines 151-6, 227-238, 346-54

(1) Doing phylogenetic analysis of plasmids in this way does not make sense - they are too recombinational and gain and lose large segments, which cannot be accommodated in a tree. This would only be useful for e.g. comparing the backbone SNPs of closely related plasmids of similar length that belong to the same Inc type. It is entirely expected that these sets of related plasmids would group by Inc type.

Authors' response: Thank you your comment. Upon careful consideration, we agree that this section lacks relevance and have therefore removed.

(2) Line 234 - what are "type I" and "type 2" plasmids.

Authors' response: Thank you for your suggestion. Based on the re-assembly results, we rephrased this section (section 2.3 and section 2.4).

6. Methods

(1) Lines 303-4 - more details are needed. Liquid or solid mating, what media, what

donor:recipient ration, time or conjugation? Or cite a suitable reference.

Authors' response: Thank you for highlighting the need for methodological clarity. We have added more details in the revised manuscript and cite a suitable reference (line319-320).

1.Boralli CMDS, et al. Characterization of *bla*$_{KPC-2}$ and *bla*$_{NDM-1}$ plasmids of a *K. pneumoniae* ST11 outbreak clone. Antibiotics (Basel) 12, 926 (2023).

Lines 309-11 - as blaNDM-13 is in the IncI1aplpha part of pSAL22057-NDM-13 and blaOXA-10 is in the IncHI2 part, finding these genes does not indicate whether or not the two plasmids have stayed together in the transconjugant. This really needs to be looked at further and if the transconjugant does contain the combined plasmid, how stable is it? This could be looked at by passaging with/without antibiotics.

Authors' response: Thank you for the valuable suggestion. Whole-genome sequencing was performed on the transconjugant to determine whether pSAL22057-NDM and pSAL22057-OXA were transferred from SAL22057 to E. coli C600 (line328-330). The pSAL22057-NDM was identified in the transconjugant via whole-genome sequencing. While conjugation experiments confirmed pSAL22057-NDM transfer to *E. coli* C600, the plasmid stability and fitness cost will be investigated in subsequent studies through serial passaging and head-to-head competition assay.

(2) Line 314 - this needs a reference

Authors' response: We have added a reference Ref 30.

7. Discussion

(1) This is too long should not simply repeat Results.

Authors' response: Thank you for the valuable suggestion. We have made more

detailed revisions to this section, making the content more substantial.

(2) Line 165 - transfer to a single E. coli only was examined here.

Authors' response: No, an extra word 'other' was written here; we have corrected this error.

(3) Lines 165-6 - blaOXA-1 is not a "significant carbapenemase gene". How do you know that the plasmid is "highly well-adapted" and this study only mentions a single plasmid.

Authors' response: In the revised manuscript, we have incorporated a discussion on the carbapenemase activity demonstrated by OXA-10. We revised the inappropriate expression "highly well-adapted".

(4) Line 171 - which slaughterhouse?

Authors' response: We have corrected this problematic expression. We rephased we rephrased this section (line174-177).

(5) Line 171-2 - are all ST29 S. Stanley?

Authors' response: No, one meropenem-resistant isolate was identified as *Salmonella Stanley* ST29.

(6) Lines 172-3 - can this really be said based on one isolate from each location?

Authors' response: Thank you for your question. We rephased we rephrased this section (line196-199).

(7) Lines 180-84 - this is not relevant, as blaOXA-10 is not a considered a carbapenemase gene.

Authors' response: Thank you for your question. We've updated the revised

manuscript to include a discussion of OXA-10's carbapenemase activity (line (line209-211).

(8) Lines 184-6 - it seems unlikely that this was the first isolate with both of these genes.

Authors' response: We rephrased as "In 2019, Han et al. described the first report of *bla*$_{NDM-1}$ and *bla*$_{OXA-10}$ co-occurring in a *Citrobacter braakii*" (line208-209).

(9) Lines 186-7 - OXA-1 has been described as having "weak carbapenemase activity". Ref 18 appears to be about OXA-10 variants that give increased ceftolozane/tazobactam and CAZ/avibactam resistance in Pseudonomas, but in fact have reduced activity against carbapenems.

Authors' response: Thanks for the comment. We have We rephrased it as "While the OXA-10 β-lactamase has been demonstrated to confer reduced susceptibility to carbapenems." in the revised manuscript (line209-211). We've updated Ref 16, 17.

(10) Line 187-8 - I may be misunderstanding this statement, but I do not believe ut. A BLAST Search shows that there are many sequences in GenBank with blaOXA-10 from Enterobacterales and many of these would likely have associated publications.

Authors' response: Thank you for the valuable suggestion. We've removed this inappropriate expression.

(11) Lines 190-1 - why does OXA-10 "warrant specific attention"?

Authors' response: We've removed this inappropriate expression.

(12) Lines 192-226 - most of this information repeats Results or should be in Results, not Discussion, and is hard to follow.

Authors' response: Based on the re-assembly, we rediscussed this section to prevent

the information repeats Results.

(13) Line 197-8 - the contents of IS640 are not relevant here.

Authors' response: We removed the contents of IS*640*.

(14) Lines 198-9 do not make sense. What is "exogeneous insertion"?

Authors' response: Thank you for your question. We've removed this inappropriate expression.

(15) Line 201 - what is meant by "externally inserted"?

Authors' response: Thank you for your question. We've removed this inappropriate expression.

(16) Lines 208-10 - again, IS640 is not really relevant.

(17) Authors' response: removed the contents of IS640

(18) Line 210 - does pSAL22057-NDM-13 have full conjugation regions of both HI2 and I1 plasmids?

Authors' response: Based on the re-assembly, pSAL22057-NDM have full conjugation regions of I1I*α* plasmid.

(19) Line 214 - what suggest this? Are there references for the related plasmids being conjugative that could be cited?

Authors' response: Thank you for your question. We've removed this inappropriate expression.

(20) Lines 217-9 - a recent paper has suggested a different function for "ter" genes (PMID: 33930099), so this needs checking

Authors' response: Thanks for the comment. The information regarding 'ter' genes is

not relevant here and has been removed.

(21) Lines 220-21 - this wording is unclear - meaning that regions 1 and 2 didn't have any segments in common?

Authors' response: Based on the re-assembly, we removed this inappropriate expression.

(22) Lines 225-6 - this is not a possible explanation - both copies of IS640 appear to be simply insertions, with flanking DR of characteristic 4 bp in length and they are not at the junctions of regions 1 and 2 claimed to be derived from the different plasmid types.

Authors' response: Thanks for the comment. We removed this inappropriate expression.

(23) Line 239 - trfF is found downstream of blaNDM in many sequences - the region around this gene is highly conserved.

Authors' response: Yes, trfF is found downstream of bla_{NDM} in many sequences. The region around this gene is highly conserved. We have mentioned this point in the revised manuscript (line 228).

(24) Lines 242,247-52 - this should refer to IS1294, not "the IS1294 transposase" that it contains. IS1294 is member of the IS91 family of rolling circle elements that are known to be able to capture and move adjacent sequences. This should be looked at properly to see if it could help to explain generation of pSAL22057-NDM-13 but see comments above about possible assembly issues.

Authors' response: Thanks for the comment. We rephased "the IS*1294* transposase" as "IS*1294*". Yes, in this study, IS*Aba125* truncated by IS*1294* was found in $bla_{\mathrm{NDM-13}}$

harboring plasmids including pSAL22057-NDM. We thus suspected that IS*1294* may be involved in the mobilization and dissemination of *bla*$_{NDM-13}$. (line233-236)

(25) Lines 243, 246 - referring to these genes by such complicated names is not helpful.

Authors' response: This information has been removed.

(26) Lines 253- - this information should be given in Results and more details of the integrons and which cassettes they contain is needed. Region 2 will not include proteins, only the genes encoding them.

Authors' response: Thanks for the comment. We added more details of the integrons in the revised manuscript (176-183). pSAL22057-NDM and pSAL22057-OXA have been revised to exclusively contain genes encoding proteins, not the proteins themselves.

(27) Line 256 - what is meant by "typical E. coli plasmid regions"?

Authors' response: We removed this inappropriate expression.

(28) Lines 257-66 - IS15 is a variant of IS26 (see PMID: 34015086) and should be named as such. The wording "...insertion sequences are derived from the IS15 transpoase tnp2" does not make sense - an IS is not derived from a gene - and where does the name tnp2 come from? The authors should look at recent papers and reviews on IS26 by RM Hall and colleagues and M. Chandler and colleagues to understand about IS26-mediated TU and "pseudocompound" transposons and how the resistance region in Region 2 could have arisen.

Authors' response: Thank you for the valuable suggestion, we rephased it as "IS*26*"

(line239-253). We thoroughly studied the relevant literature by RM Hall and colleagues and M. Chandler and colleagues and cited it in the revised manuscript.

1.Harmer CJ, Hall RM. IS*26* and the IS*26* family: versatile resistance gene movers and genome reorganizers. *Microbiol Mol Biol Rev*. **88**(2): e0011922 (2024).

(29) Line 262 - "TnAs3 tnpR" matches Tn21, which is a well-known Tn that carries an integron, so it would be better to use this name (also on Figures).

Authors' response: Based on the re-assembly, we removed this inappropriate expression.

(30) Lines 267-70 - which plasmid has the inverted region with blaNDM-5 that is mentioned? Is there any clue about how this inversion might have occurred? Why would blaNDM-5 be acquired and by what?

Authors' response: Based on the re-assembly, we removed this inappropriate expression.

8. Other scientific points

(1) Line 43 - "other E. coli species" does not make sense - E. coli is a species. Do you mean other strains? If so, it seems that conjugation of pSAL22057-NDM-13 to EC600 only was tested.

Authors' response: Thanks for the comment. We rephased it as "Conjugation experiments revealed that $\textit{bla}_{\text{NDM-13}}$ was transferrable to *E. coli* C600."

(2) Line 46-7 - does identification of a single isolate really make monitoring of it "urgent" and how could it be "controlled"?

Authors' response: Thanks for your question. we removed this inappropriate expression.

(3) Line 69 - this is out of date. The NCBI Reference Gene Catalog now lists 75 NDM variants (https://www.ncbi.nlm.nih.gov/pathogens/refgene/#blaNDM)

Authors' response: Thanks for the comment. we removed this inappropriate expression.

(4) Lines 71-2 - a quick BLASTn search with the blaNDM-13 sequence from the Reference Gene Catalog identifies 10 sequences with this gene, all from Enterobacterales, not including pSAL22057-NDM-13.

Authors' response: We thank the reviewer for highlighting this point, we removed this inappropriate expression.

(5) Lines 96-8 - I couldn't find blaTEM-60 on the chromosome and only found blaTEM-1b, not blaTEM-95, on pSAL22057-NDM-13 and these genes didn't come up in a ResFinder analysis - this needs checking.

Authors' response: Thank you for the valuable suggestion. This study systematically re-analyzed antimicrobial resistance gene annotations through a multi-database cross-validation strategy, integrating ResFinder v4.7.2 (based on core genome alignment) with CARD v3.2.5 (employing a strict domain validation model). Furthermore, phenotype-genotype association discrepancies in the initial draft were calibrated according to the clinical breakpoint standards (CLSI 2023). Through multi-database validation, only the TEM-1B gene was definitively identified in this strain. We made corrections in the revised manuscript (line135-136), Table-1.

(6) Lines 101-2 - this is the wrong place for this information - it should be given before WGS is discussed, especially if this is how the Stanley serotype was first

identified.

Authors' response: We moved this information to section 2.1 (line 94-95).

(7) Lines 103-10 - this section really doesn't say much. Could anything more be made of it? For example, is anything much known about SPIs in S. Stanley?

Authors' response: We appreciate the reviewer's constructive feedback and agree that further contextualization of *Salmonella* pathogenicity islands (SPIs) in *S.* Stanley would strengthen this section (line 141-150). While *S.* Stanley is less extensively studied than serovars like *S.* Typhimurium or *S.* Enteritidis, genomic analyses reveal that it shares core conserved SPIs (e.g., SPI-1, SPI-2, SPI-3) essential for host invasion, intracellular survival, and magnesium uptake (*mgtBC*). However, its accessory SPIs (e.g., SPI-4, SPI-5, SPI-14, CS54 island) exhibit serovar-specific variations that may influence niche adaptation. Unlike well-characterized serovars, SPI-9, SPI-13, and SPI-14 in *S.* Stanley SAL22057 lacked annotated virulence factors in VFDB. No virulence gene CDS were identified in SPI-9, SPI-13, and SPI-14; consequently, their functions have not been thoroughly characterized. We will investigate this aspect in future studies.

(8) Lines 298-9 - would these primers detect all variants of blaIMP and blaVIM? The blaOXA group in particular is very broad and includes many subgroups - which types would be detected by the primers used? blaOXA-48-like? Ref 23 needs to be checked and this information stated correctly. We used these primers to screen for common carbapenemase genes

Authors' response: These primers can't detect all variants of bla_{IMP} and bla_{VIM}. Yes, it

is *bla*$_{OXA-48-like}$ (line283-284). We used these primers to screen for common carbapenemase genes (*bla*$_{NDM}$, *bla*$_{KPC}$, *bla*$_{VIM}$, *bla*$_{IM}$, and *bla*$_{OXA-48-like}$), with definitive confirmation of detected genes established through whole-genome sequencing results. We have checked Ref 23 and updated it to a new source.

(9) Line 311 - what is meant by "conjugation elements"?

Authors' response: We rephased "conjugation elements" as "bacterial mobile genetic elements".

(10) Lines 371-4 - the sequences in GenBank are not annotated. It would be helpful to add annotations.

Authors' response: Although the sequences in GenBank have been annotated, the SAL22057 chromosome sequence and its features are not displayed in NCBI due to its large size.

9. Tables and Figures

Table 1

(1) Title - strictly speaking, it is the antibiotics that have the MICs, not the isolates, should "transmant" be "transconjugant" and "recipi" at the end is missing letters. The table might be better presented with the rows and columns transposed, i.e. antibiotic names down the side and a column for each strain. Why only mention beta-lactamase genes when MICs are also given for other antibiotic types?

Authors' response: We sincerely thank the reviewer for their meticulous reading and expert guidance. All noted issues in Table 1's caption have been comprehensively addressed. We appreciate the reviewer's astute observation. The focus on beta-lactamase genes (*bla*$_{NDM-13}$ and *bla*$_{OXA-10}$) stems from the primary objective of this

study: to characterize pSAL22057-NDM and pSAL22057-OXA and their role in carbapenem resistance. The inclusion of MICs for non-β-lactam antibiotics served two key purposes: Clinical Context: To demonstrate the isolate's multidrug-resistant (MDR) phenotype, which is critical for understanding its therapeutic challenges (Table1).

Figure 1

(1) The text on the diagram is hard to read. This figure might be better as supplementary.

Authors' response: Thank you for your valuable feedback on the figures. We have increased the font size and improved the contrast of all text labels within the diagrams to ensure they are clearly legible.

(2) Line 451 - this should be "dashed" not "dash", but the lines don't look dashed.

Authors' response: We thank the reviewer for catching this inconsistency. The term "dash" has been replaced with "dashed" throughout the text and figure legends (e.g., Figure 1A/2A). Additionally, we regenerated all affected figures using vector-based formats (PDF) with explicit dashed-line parameters (dash length: 5 pt, gap: 2 pt) to ensure visual clarity. The revised figures are now included in the resubmitted manuscript.

Figure 2

(1) The text on the diagrams is hard to read. A scale of % identity is only shown on Part B and different colours are used in other parts - it would be better to be consistent across figures and there should be something to explain what all of the coloured shading means.

Authors' response: A scale of % identity has now been added to all relevant parts of the figures (including Parts Figure1B, Figure2B, etc., where applicable) for consistency and clarity. We have standardized the color scheme used across all parts of the figures. Furthermore, a comprehensive legend explaining the meaning of all colored shadings has been added to each figure panel (or a single clear legend is now provided where appropriate).

(2) Part A - "backbone", rather than "skeleton" is normally used for the parts of plasmids carrying genes encoding plasmid functions in which insertions are found.

Authors' response: You are correct. Thank you for highlighting this important distinction—accurate terminology ensures clarity in scientific communication.

(3) Part B - the purpose of comparing these sequences is not really clear from the text.

Authors' response: Through a comparative analysis of the flanking structures of *bla*$_{NDM-13}$, we investigated the factors influencing its dissemination. These findings are presented in Results section 2.3 and discussed in the corresponding Discussion section (Lines 227-238).

(4) Part C - in pSAL22057-NDM-13, the first IS26 (labelled as "IS15-tnp2") starts at 173167 and the last one ends at 251001, so it is not clear why the positions stated here are 173100-251000? This also needs checking for the other sequences shown.

Authors' response: We have thoroughly checked all the sequences referenced above (see Figure2B).

Figure 3

(1) See comments on the tree part above.

Authors' response: Based on the comments 5 above, we removed the tree part.

Supplementary

Table 1

This is not enough information to merit a table - all of the information is already stated in text, or could be, and it is organized in an odd way. All parts of resistance gene names should be in italics, except bla gene subscripts, and QnrS1 should be qnrS1. See comment above on blaTEM in chromosome.

Authors' response: We agree that the table was redundant. The table has been deleted, and all essential information is now concisely presented in the text (Section 2.2, Lines 125-139). All resistance gene names have now been standardized according to scientific convention: (i) *bla*$_{\text{NDM-13}}$, *bla*$_{\text{TEM}}$, *qnrS1* are now fully italicized (ii) Subscripts (e.g., NDM-13, TEM) remain in non-italicized roman font (iii) *qnrS1* has been corrected to lowercase (formerly "*QnrS1*").

Table S2

All letters in gene names should be italicized. The first letters of protein names should be upper case.

Authors' response: Thank you for highlighting this important detail. We confirm that all gene and protein nomenclature has been standardized throughout the manuscript according to scientific convention: (i) Gene names are now fully italicized (with subscripts in non-italic Roman font). (ii) Protein names consistently use initial capitalization only (no italics) (Table S1).

10. Minor/formatting/English etc

(1) Salmonella serovars should be in plain text, not italics so "Stanley" needs to be changed throughout.

Authors' response: Thank you for the correction. We have updated the manuscript: "Stanley" (serovar) is now in plain text throughout the manuscript, consistent with standard Salmonella serovar nomenclature.

(2) Line 34-5 - wording does not make sense - "genomic characterization" is not "examined".

Authors' response: We rephased "examined" as "analyzed".

(3) Line 37 - should be "(MIC 32 ug/ml). (MIC 16 ug/ml)".

Authors' response: Thank you for highlighting this discrepancy. The corrected now reads: meropenem (MIC 32μg/mL) and imipenem (MIC 16μg/mL).

(4) Line 38 - should be "belongs to".

Authors' response: Thank you for pointing out this important clarification. We have revised the phrasing to "belongs to" in the relevant sections to ensure terminological accuracy.

(5) Lines 40-2 - suggest "analysis identified blaNDM-13 on a conjugative hybrid plasmid with HI2 and I1alpha replicons", but using an alpha symbol.

Authors' response: We have revised the text to clarify this distinction and corrected 'alpha' to the Greek symbol 'α' for scientific accuracy.

(6) Lines 53-4 - wording does not make sense.

Authors' response: We rephased it as "*Salmonella* spp are common pathogens that infect humans when contaminated food or water is consumed" (line 74-75)

(7) Lines 57-60 - the gene names should be used here (i.e. blaKPC etc, correctly formatted) and there is no need to use the full names - these are all well-known genes - and presumably "or blaOXA-48-like genes" is meant?

Authors' response: Thank you for your feedback. We have revised the text to: (a) Use standard gene symbols (e.g., bla_{KPC}, bla_{IMP}) instead of full names, (b) Correctly format all gene symbols (italicized bla with non-italic allele), and (c) Clarify $bla_{\mathrm{OXA\text{-}48}}$ as '$bla_{\mathrm{OXA\text{-}48\text{-}like}}$ genes' to reflect genetic variants.

(8) Line 63 - this wording is quite awkward.

Authors' response: Thank you for your feedback. We agree the original phrasing was convoluted. We rephased it as "The prevalence of metallo-*β*-lactamases (MBLs) remains a major clinical challenge: these enzymes hydrolyze *β*-lactams and are unaffected by available *β*-lactamase inhibitors (e.g., avibactam, relebactam,

vaborbactam).” (line81-83)

(9) Line 67 - "lactamase" not "lactamases".

Authors’ response: We rephased "lactamases" as "lactamase".

(10) Line 78 - heading should be "Results".

Authors’ response: Yes, we rephased it as “Results”

(11) Lines 80, 285 - presumably "one and a half year old"? "18 month old" might be better.

Authors’ response: Thank you for your suggestion. we rephased "one and a half year old" as “18-month-old”(line 77, 248)

(12) Line 83 - if all antibiotics are listed then "including" is incorrect here. If some are not listed then they should be added, but this list might not be needed here and could just be given in Methods. The normal abbreviation for cefotaxime is CTX, not CXT (also Line 293).

Authors’ response: We removed “including”. The modifications have been made following the suggestions above (line109, 277).

(13) Line 91 - "detection of"

Authors’ response: We added “of” after detection.

(14) Lines 93-4 - suggest "to have a 4,847,514 bp chromosome and". Why were the plasmids numbered in this order? It would probably have been more logical to do this e.g. in order of decreasing size.

Authors’ response: Thank you for your valuable suggestions. We have: (a) Revised the chromosome description per your recommendation, and (b) Renumbered the plasmids in descending size order to improve logical consistency, while retaining functional annotation for the *bla*$_{NDM-13}$-harboring plasmid."(line 125-128)

Line 96 - blaTEM genes encode beta-lactam resistance, not beta-lactams.

Authors’ response: Thank you for your valuable suggestions. Based on the reassembled genome, this section/content has been removed.

(15) Line 97 - remove "The" from start of sentence.

Authors’ response: We removed “the”.

(16) Line 106 - should be "harbors".

Authors’ response: We rephased “harbor” as “harbors”.

(17) Line 112 - "simultaneously" is not needed.

Authors’ response: We removed it.

(18) Line 113 - delete "of".

Authors’ response: We removed it.

(19) Line 147 - delete "the" before blaNDM-13.

Authors’ response: We removed it.

(20) Line 158 - the gene name is blaNDM-1. The wording here could be simplified e.g. "The first example of blaNDM-1 in Salmonella was documented..."

Authors’ response: The modifications have been made following the suggestions above (line165).

(21) Line 160 - what is meant by "genetic backgrounds" here?

Authors’ response: We have revised the text clarify this as “NDM has been detected in two *S.* Stanley with distinct plasmid contexts (IncA/C/IncHI2), isolated from…” (line166-167)

(22) Line 162 - again, this should refer to the blaNDM gene, and is being found in a single isolate really "emerging"? (also applies to the manuscript title).

Authors’ response: Thank you for your valid critique. We have: (a) Corrected gene nomenclature to $\textit{bla}_{\text{NDM-13}}$ and $\textit{bla}_{\text{OXA-10}}$ throughout, (b) Replaced 'emergence' with 'identification' (line 168-169) (c) Revised the title to: 'Novel Co-occurrence of $\textit{bla}_{\text{NDM-13}}$ and $\textit{bla}_{\text{OXA-10}}$ in a Carbapenem-Resistant *Salmonella* Stanley ST29 Strain from China'. (line 1-2)

(23) Lines 235-6 - "conjugative transfer"

Authors’ response: We removed it.

(24) Lines 276-7 - "within the SAL22057 strain" is not needed.

Authors’ response: We removed it.

(25) Line 285 - it was not known at this stage that SAL22037 is S. Stanley?

Authors' response: Thank you for this important clarification. We confirm that species identification was performed after isolation through whole-genome sequencing (WGS) and serotyping. We have revised the text as "An isolate designated SAL22057 was isolated from an 18-month-old boy's fecal sample…" (line 270)

(26) Line 286 - presumably this was species identification?

Authors' response: Thank you for highlighting this ambiguity. We confirm that both MALDI-TOF MS and 16S rRNA sequencing were used specifically for species-level identification of isolate SAL22057. The text has been revised to explicitly state this purpose. Revised sentence: Species identification of SAL22057 was performed using the Bruker Biotyper MALDI-TOF MS (Bruker Daltonik GmbH, Bremen, Germany) and confirmed by 16S rRNA gene sequencing. (line 271-273)

(27) Lines 292-3 - the abbreviation for ceftazidime was given on Line 84 and MICs are not "performed".

Authors' response: Thank you for these corrections. We have: (a) Removed the redundant abbreviation '(CAZ)' after ceftazidime (since previously defined on Line 84), (b) Replaced 'performed' with the appropriate verb 'determined' for MIC testing, and (c) Ensured consistency for all antibiotic abbreviations. (line 255-257)

(28) Line 295-6 - lowercase c for carbapenemase and a reference for this method is needed

Authors' response: Thank you for these corrections. We corrected 'Carbapenemase' to lowercase 'carbapenemase' and added the reference for the phenotypic confirmation method.

1 Tamma PD, Simner PJ. Phenotypic Detection of Carbapenemase-Producing Organisms from Clinical Isolates. J Clin Microbiol. 2018 Oct 25;56(11): e01140-18.

(29) Line 299 - "PCR positive products" does not make sense.

Authors' response: Thank you for highlighting this incorrect phrasing. We confirm that 'PCR-positive samples' was intended, not 'products.

(30) Line 302 - this section does not mention plasmid sequencing.

Authors' response: Thank you for catching this inconsistency. We removed the "plasmid sequencing".

(31) Lines 303-4 - which plasmid?

Authors' response: Thank you for highlighting this ambiguity. We rephased this section. (line 296-308)

(32) Line 305 - "transconjugants" not "conjugants"

Authors' response: Thank you for highlighting this incorrect phrasing. We rephase "conjugants" as "transconjugants".

(33) Line 307 - "identified as E. coli" and the MALDI-TOF details have already been given on Line 287-8.

Authors' response: We removed this redundant sentence.

(34) Lines 322-3 - wording does not make sense - words missing?

Authors' response: Thank you for identifying this unclear phrasing. We have revised the sentence to explicitly specify that Pilon was used to polish the draft assembly using second-generation reads (line294-305). The improved version clarifies both the input material and action performed.

(35) Lines 337-42 - this reads more like it should be in Results.

Authors' response: Thank you for your valuable feedback. We agree with the reviewer's point. The sentence describing the specific findings (plasmid types, presence of $bla_{\mathrm{NDM-13}}$, and resistance genes) has been moved from the Methods section 5.6 to the Results section 2.3 as suggested. (line152-1155)

(36) Lines 347-9 - the wording here does not make sense and seem like more like Results than Methods.

Authors' response: Based on the comments 5 above, we removed section 5.7.

(37) References - change Title Case to Sentence case (e.g. Refs 3, 4,9, 10) and check that species and gene names etc. are correctly formatted. The citation for Ref2 is in the wrong format.

Authors' response: Thank you for your careful review. We have addressed all formatting points raised.

Reviewer #2 (Comments for the Author):

In this manuscript, Zhou et al. isolated and characterized a NDM-13 and OXA-10 co-producing Salmonella Stanley ST29 clinical strain, with the blaNDM-13 and blaOXA-10 genes on a newfound transmissible conjugative hybrid plasmid pSAL22057-NDM13, highlighting the urgent necessity for monitoring and controlling of clinical strains co-producing NDM-13 and OXA-10. The study is of clinical importance, and my concerns are minus.

1) Language editing is necessary. For example, line 36 "testing indicated" should be as "testing showed", line 37 "(MICs of 32µg/ml)" should be as "(MICs of 32 µg/ml), Lines 77, "strain isolated a child with 77 acute diarrhea and fever" should be as "strain isolated from a child with 77 acute diarrhea and fever".

Authors' response: "Thank you for your careful review and valuable language suggestions. We have implemented all edits as recommended: Line 36: Changed "testing indicated" → "testing showed". Line 36: Corrected "(MICs of 32µg/ml)" → "(MICs 32 µg/ml)" (added space between value and unit). Line 37: Revised "strain isolated a child" → "strain isolated from a child" (added missing preposition "from"). We have also conducted a thorough proofread of the manuscript to ensure consistency in terminology, unit formatting (e.g., µg/mL, mg/L), and grammatical accuracy. Your attention to detail has significantly improved the clarity of our work.

2) So far, any signs of existence of the newly identified plasmid in other clinical strains except for SAL22057?

Authors' response: We appreciate this important question. To address this concern, we rigorously validated the genome assembly. The genome assembly method is detailed in Section 5.4. Re-assembly revealed that strain SAL22057 was found to harbor one chromosome and six plasmids named pSAL22057-OXA (2376, 376bp, carrying

bla$_{OXA-10}$), pSAL22057-3 (93, 293bp), pSAL22057-NDM (87,928bp, carrying *bla*$_{NDM-13}$), pSAL22057-4 (6, 477bp), pSAL22057-5 (5, 423bp) and pSAL22057-6 (4, 583bp). These plasmids are also present in other strains.

Reviewer #3 (Comments for the Author):

This study analyses a strain of Salmonella Stanley ST29 that produces both NDM-13 and OXA-10 enzymes, which is relatively rare and informative for understanding resistance in Salmonella spp. There are several comments for reference.

1. Line 96-98: How to explain the presence of TEM genes on both chromosome (blaTEM-60) and plasmid (blaTEM-1 and blaTEM-95)? Is it because the threshold was set too low during sequencing sequence analysis and not filtered effectively resulting in false positive for blaTEM resistance genes?

Authors' response: Thank you for the valuable suggestion. This study systematically re-analyzed antimicrobial resistance gene annotations through a multi-database cross-validation strategy, integrating ResFinder v4.7.2 (based on core genome alignment) with CARD v3.2.5 (employing a strict domain validation model). Furthermore, phenotype-genotype association discrepancies in the initial draft were calibrated according to the clinical breakpoint standards (CLSI 2023). Through multi-database validation, only the TEM-1B gene was definitively identified in this strain. We made corrections in the revised manuscript (line133-135), Table-1.

2. Line 92-110: '2.2' and '2.3' can be combined into one paragraph.

Authors' response: Thank you for your valuable feedback. We have combined sections 2.2 and 2.3 into a single streamlined paragraph (now Section 2.2) as recommended.

3. Line 111-156: Paragraphs '2.4' and '2.5' could be combined into one paragraph.

Authors' response: We thank the reviewer for the suggestion to consolidate sections. Upon rigorous reassessment, Section 2.5 (phylogenetic analysis of plasmids) has been deleted. Because the plasmids are too recombinational and gain and lose large

segments, which cannot be accommodated in a tree.

4. although there is a description of 'Conjugation Assay' in the Methodology section, there is no relevant information in the Results section, it is recommended to describe it separately in the Results section or combine it with the results of the antimicrobial susceptibility testing.

Authors' response: We appreciate the reviewer's valuable observation regarding the conjugation assay results. We integrated the conjugation assay results with the antimicrobial susceptibility testing data (line 118-123, Table1).

5. The description in the Discussion section should be more concise, and part of the description should be repeated with the results.

Authors' response: Thank you for the valuable suggestion. We rephrased the Discussion. Repetitive result summaries have been deleted; all data presentation now resides exclusively in the Results section.

6. It is recommended to add information about the medical history, the antimicrobial drugs used to treat the infection, and the prognosis.

Authors' response: We thank you for their insightful suggestions. We add the information about the medical history, the antimicrobial drugs used to treat the infection, and the prognosis in section 2.1. (line101-108).

7. blaTEM-60 is missing from the resistance genes in Table 1, and blaTEM-60 is an extended-spectrum enzyme that can hydrolyse aztreonam. However, the results of the susceptibility testing show that the bacteria are susceptible to aztreonam, how can this be explained?

Authors' response: Thank you for highlighting this important issue. We've re-analyzed the TEM-60 annotation using annotation using ResFinder (v4.7.2, >99% identity threshold) and CARD's domain validation, confirming the absence of Ambler Class A β-lactamase markers in this strain. We removed *bla*$_{\text{TEM-60}}$ in the revised manuscript (line133-135), Table-1.

Re: Spectrum03207-24R1 (**Novel Co-occurrence of *bla*$_{NDM-13}$ and *bla*$_{OXA-10}$ in a Carbapenem-Resistant *Salmonella* Stanley ST29 strain in China**)

Dear Mrs. Juanjuan Zhou:

Thank you for the privilege of reviewing your work. Below you will find my comments, instructions from the Spectrum editorial office, and the reviewer comments.

Please return the manuscript within 60 days; if you cannot complete the modification within this time period, please contact me. If you do not wish to modify the manuscript and prefer to submit it to another journal, notify me immediately so that the manuscript may be formally withdrawn from consideration by Spectrum.

Revision Guidelines
To submit your modified manuscript, log into the submission site at https://spectrum.msubmit.net/cgi-bin/main.plex. Go to Author Tasks and click the appropriate manuscript title to begin. The information you entered when you first submitted the paper will be displayed; update this as necessary. Note the following requirements:

• Upload point-by-point responses to the issues raised by the reviewers in a file named "Response to Reviewers," NOT in your cover letter.
• Upload a compare copy of the manuscript (without figures) as a "Marked-Up Manuscript" file.
• Upload a clean .DOC/.DOCX version of the revised manuscript and remove the previous version.
• Each figure must be uploaded as a separate, editable, high-resolution file (TIFF or EPS preferred), and any multipanel figures must be assembled into one file.
• Any supplemental material intended for posting by ASM should be uploaded with their legends separate from the main manuscript. You can combine all supplemental material into one file (preferred) or split it into a maximum of 10 files with all associated legends included.

For complete guidelines on revision requirements, see our Submission and Review Process webpage. Submission of a paper that does not conform to guidelines may delay acceptance of your manuscript.

Data availability: ASM policy requires that data be available to the public upon online posting of the article, so please verify all links to sequence records, if present, and make sure that each number retrieves the full record of the data. If a new accession number is not linked or a link is broken, provide Spectrum production staff with the correct URL for the record. If the accession numbers for new data are not publicly accessible before the expected online posting of the article, publication may be delayed; please contact production staff (Spectrum@asmusa.org) immediately with the expected release date.

Publication Fees: For information on publication fees and which article types are subject to charges, visit our website. If your manuscript is accepted for publication and any fees apply, you will be contacted separately about payment during the production process; please follow the instructions in that e-mail. Arrangements for payment must be made before your article is published.

ASM Membership: Corresponding authors may join or renew ASM membership to obtain discounts on publication fees. Need to upgrade your membership level? Please contact Customer Service at Service@asmusa.org.

The ASM Journals program strives for constant improvement in our submission and publication process. Please tell us how we can improve your experience by taking this quick Author Survey.

Thank you for submitting your paper to Spectrum.

Sincerely,
Jian Peng
Editor
Microbiology Spectrum

Reviewer #1 (Comments for the Author):

This revised manuscript describes analysis of a Salmonella Stanley isolate that carries blaNDM-13 and other resistance genes, including blaOXA-10. The original version stated that blaNDM-13 and blaOXA-10 were on the same large plasmid with HI2, HI2A and I1alpha replicons. After checking assemblies, the authors have now concluded that that blaNDM-13 is on an I1 plasmid and blaOXA-10 on an HI2 plasmid, so this revised version has substantial differences from the original.

The authors have also included further justification for the focus on blaOXA-10, but it seems unlikely that this gene to contributes to carbapenem resistance in this isolate and no direct evidence is presented relating to this, so I believe that this aspect should not be a main focus of the manuscript.
Closer attention to detail is needed to avoid simple errors and the wording/English still needs attention in places. To reduce the number of comments, I have tried to group those that relate to the same point and have not included original comments that were adequately addressed.

1) Focus on blaOXA-10 as a "carbapenemase"
ORIGINAL COMMENT: blaOXA-10 is also mentioned in several places but as this gene is not considered to encode a carbapenemase the reason for focusing on it is unclear (many other AMR genes are also present).
AUTHORS' RESPONSE: When expressed in Acinetobacter baumannii ATCC 17978, OXA-10 conferred high-level resistance to carbapenems, with MIC values for meropenem, ertapenem, and doripenem comparable to those of recognized carbapenemases like OXA-48 and higher than OXA-58. For example, the MICs of meropenem, ertapenem, and doripenem were 16 μg/ml, 128 μg/ml, and 16 μg/ml, respectively, indicating clinical resistance levels. Although OXA-10 has low hydrolysis efficiency for carbapenems, it exhibits high substrate affinity (nanomolar dissociation constants), consistent with carbapenemase characteristics. In Acinetobacter baumannii (with low membrane permeability and efflux pumps), it showed significant resistance, a hallmark of carbapenemase. OXA-10 was expressed 25-fold more highly in Acinetobacter baumannii than OXA-48. High expression levels offset lower catalytic efficiency, leading to equivalent resistance. In summary, OXA-10 has both carbapenem-hydrolyzing ability and clinical resistance phenotypes, qualifying it as a carbapenemase1. OXA-10 and its variants (e.g., OXA-655) exhibit carbapenem-hydrolyzing activity, host-dependent clinical resistance, and evolutionary plasticity, meeting the criteria for classification as carbapenemases2. Thus, OXA-10 showed to be weak carbapenemases. We added this part of the content in the revised manuscript (line 86-90).
1） Antunes NT et al. Class D β-lactamases: are they all carbapenemases? Antimicrob Agents Chemother 58, 2119-25(2014).
2） Kotsakis SD et al. Characterization of the First OXA-10 Natural Variant with Increased Carbapenemase Activity. Antimicrob Agents Chemother. 63, e01817-18(2018).
NEW COMMENTS: I still believe that the focus on OXA-10 as a carbapenemase and apparently suggesting that it is equivalent to NDM and other "classic" carbapenemases is not appropriate. Ref 1 above states "Here we demonstrate that when OXA-2 and OXA-10 are expressed in Escherichia coli strain JM83, they produce a narrow-spectrum antibiotic resistance pattern. When the enzymes are expressed in A. baumannii ATCC 17978, however, they behave as extended-spectrum-lactamases and confer resistance to carbapenem antibiotics." The isolate being studied here is Salmonella, which is part of the Enterobacterales.
Ref 2 is about a specific variant, OXA-655, not OXA-10 itself. Of the 70 OXA-10 alleles listed in the NCBI Reference Gene Catalog (https://www.ncbi.nlm.nih.gov/pathogens/refgene/#OXA-10) only two (OXA-233 and blaOXA-655) are listed as having carbapenemase activity. blaOXA-655 in Ref 2 above was also found on an IncQ plasmid, which might have a high copy number contributing to greater resistance. Ref 2 also states "While carbapenem resistance in Gram-negative bacteria is mainly due to the production of efficient carbapenemases, beta-lactamases with a narrower spectrum may also contribute to resistance when combined with additional mechanisms. OXA-10-type class D-lactamases, previously shown to be weak carbapenemases, could represent such a case."
No evidence is presented here that that OXA-10 contributes to the carbapenem resistance phenotype in SAL22057, indeed Lines 115-6 state that "the presence of a metalloenzyme in SAL22057 explains the carbapenem resistant phenotype".
The original manuscript might suggest a difference between the MEM MIC in SAL22057 (32) and the transconjugant (NDM-13 only, 16) but there are inconsistencies in the MICs of IPM and MEM between Table 1 in the original manuscript (SAL22057, IPM 16, MEM 32; transconjugant, IPM 16, MEM 16) and Table 1 in the revised manuscript (SAL22057, IPM >16, MEM >16; transconjugant, IPM >16, MEM >16) and between Table 1 and the text in the revised manuscript (Lines 37, 57 SAL22057 IPM 16, MEM 32, Lines 121-2 transconjugant IPM 16, MEM 16). This doesn't seem to be explained in the response, so which numbers are correct?
To show any contribution of OXA-10 to carbapenem resistance in SAL22057 curing pSAL22057-NDM, leaving only pSAL22057-OXA would probably be needed. As the chromosome sequence is available it would be possible to look for chromosomal mutations that could contribute to carbapenem resistance.
If authors want to suggest that blaOXA-10 in a mobile region on plasmid in Salmonella is concerning because this gene could spread/mutate and contribute to carbapenem resistance in this (and maybe other) species under certain conditions, then there needs to be more justification of the likely importance of this and it needs to be explained much more clearly and briefly as part of the Discussion only, and should not be the main focus of the manuscript.
SPECIFIC POINTS RELATING TO COMMENTS ABOVE:
Title - should be updated after changes have been made.
Line 54 - I don't think that it can be argued that blaOXA-10 is "critical" in SAL22057.
Lines 59-60 "dual carbapenemase" is not an appropriate description.
Lines 86-90 - need rewriting to be more accurate and reflect comments above.
Lines 90-2 - this should be deleted, as the relevance of isolates with two "classic" carbapenemases to thsi manuscript is not clear.
Lines 165-6 - blaOXA-10 is not considered a "significant carbapenemase gene". How do you know that the plasmid is "highly well-adapted" and this study only mentions a single plasmid.
Lines 194-5 - needs further explanation and/or toning down. What is the "strain" being referred to and what is the evidence that is adaptable and transmissible?
Lines 205-8 - this is not relevant. The multiple carbapenemases being referred here to are "classic" carbapenemases.

Lines 209-11 - this is not a complete sentence and is inaccurate, as OXA-10-like enzymes only gives reduced susceptibility under certain conditions (e.g. in Acinetobacter, if they have a specific mutation) and are 16 and 17 the correct references here, or should it be 6 and 7?

2) pSAL22057-NDM, Lines 152-169, including role of IS1294, Lines 42-3, 61-2, 163-9, 227-238, 259-60
ORIGINAL COMMENT: Lines 242,247-52 - this should refer to IS1294, not "the IS1294 transposase" that it contains. IS1294 is member of the IS91 family of rolling circle elements that are known to be able to capture and move adjacent sequences. This should be looked at properly to see if it could help to explain generation of pSAL22057-NDM-13 but see comments above about possible assembly issues.
AUTHORS' RESPONSE: We rephased "the IS1294 transposase" as "IS1294". Yes, in this study, ISAba125 truncated by IS1294 was found in blaNDM-13 harboring plasmids including pSAL22057-NDM. We thus suspected that IS1294 may be involved in the mobilization and dissemination of blaNDM-13. (line233-236).
NEW COMMENT: As stated in the original comment, IS1294 is a member of the IS91 family, rolling circle elements that are known to be able to move adjacent regions (see PMID: 10873528). It should be possible to tell fairly confidently if IS1294 was responsible for inserting part of Tn125 into the pSAL22057-NDM, so this needs to be looked at properly. On a quick look, it seems like MN219406 and CP092912 have a clear insertion vs. CP037994, while pSAL22057-NDM has a deletion in this region. If there are papers associated with MN219406, CP092912 and/or CP037994 then these should be examined for any mention of an IS1294 mediated event and they should be cited.
ORIGINAL COMMENT: Lines 112-3 - IncI plasmids have a shufflon that may not be correctly assembled, even from long read data (see PMID: 28424528). This needs to be looked at and may explain some differences from other IncI1 plasmids.
NEW COMMENT: There was no specific response to this.
OTHER NEW COMMENTS:
Line 162 - "pgs_wt5" does not appear to be a complete plasmid
Line 233 - it's more than IRR - it's 51 bp of the IRR end. Does this segment include the promoter?
Figure 1
NEW COMMENT: I would still suggest that Part A be moved to supplementary. There seems to be something wrong with the top of Part B, with part of the text moved to the right. The equivalent region of pgs_wt5 should also be shown (see comments above). DR are shown in the key, but no DR seem to be shown in the diagrams?
Legend
Lines 437-8 - wording does not make sense. "Comparison" not "Characterization"? "closely" not "closed".
Lines 439, 442-3 - there is no need to give each accession no. twice.
Line 445 - "resistance genes" is normally used, "Inserion sequences" needs correcting, or just use "IS"

3) pSAL22057-OXA with blaOXA-10 and other resistance genes.
NEW COMMENTS: A much clearer/more accurate description of the resistance regions of pSAL22057-OXA is still needed e.g., Line 176 still refers to "the region", which does not make sense, and "integron 3" (Line 181) is unclear without further explanation. The AMR genes currently listed on Lines 133-5 could be stated here instead, indicating which are in the same cassette array and which others are nearby. Could any IS/Tn explain insertions, are there any DR etc. Again, are there any papers associated with the related plasmids listed in this section that might already have information on this that could be consulted and cited. Information on Lines 245-8 need to be in Results not Discussion.
ORIGINAL COMMENT: Lines 128-9 - the information about IS640 isn't relevant to this manuscript. It would be more useful to look for flanking direct repeats, as these would give evidence of a simple insertion, and state if these are present (also Lines 135-6).
AUTHORS' RESPONSE: We have removed the information about IS640 in the revised manuscript. We have identified the inverse and direct repeats within in the region of blaNDM-13 (pSAL22057-NDM) and blaOXA-10 (pSAL22057-OXA), and these have been annotated in the updated Figure 1B/2B. For further information, please consult Table S3, S4. IS26 features two flanking 8-base pair direct repeats (DRs): TCGCAGGA is adjacent to the left inverted repeat (IRL), and CTTTTTTG is adjacent to the right inverted repeat (IRR).
NEW COMMENT Lines 183-5: It is not clear from the text which IS26 is being referred to here, but the "DR" shown on Fig. 2 seem to be flanking the IS26 downstream of dfrA14. The 8 bp sequences immediately flanking this copy of IS26 are CGCAGGAC (not TCGCAGGA) and CTTTTTTG but these are not DR - the name "direct repeats" is because they have the same sequence. As these "DR" are incorrect, and the ends of a particular IS are always the same, Tables S3 and S4 do not include any useful information and should be removed.
ORIGINAL COMMENT: Line 210 - does pSAL22057-NDM-13 have full conjugation regions of both HI2 and I1 plasmids?
AUTHORS' RESPONSE: Based on the re-assembly, pSAL22057-NDM have full conjugation regions of I1Iα plasmid.
NEW COMMENT: What about the conjugation region of pSAL22057-OXA?
ORIGINAL COMMENT: Line 262 - "TnAs3 tnpR" matches Tn21, which is a well-known Tn that carries an integron, so it would be better to use this name (also on Figures).
AUTHORS' RESPONSE: Based on the re-assembly, we removed this inappropriate expression.
NEW COMMENT: "TnAs3-tnpA" is still shown on Fig. 2, so needs to be corrected/explained.
ORIGINAL COMMENT: Lines 267-70 - which plasmid has the inverted region with blaNDM-5 that is mentioned? Is there any clue about how this inversion might have occurred? Why would blaNDM-5 be acquired and by what?
AUTHORS' RESPONSE: Based on the re-assembly, we removed this inappropriate expression.
NEW COMMENT: Inverted regions are shown in Fig. 2 still and need to be explained.

OTHER NEW COMMENTS:
Lines 41, 130, 171 - HI2 and HI2A are separate replicons, typically found together on an HI2 plasmid, and this needs to be correctly written throughout the manuscript.
Lines 176-9 - why mention the "functionally uncharacterized proteins"? The different IS and Tn should be properly identified.
Line 179 - homology is not quantifiable. Two things are either homologous or not. Do you mean high sequence identity? And wouldn't "an equivalent segment" always be closely related?
Figure 2
NEW COMMENT: Part A could be made supplementary. See comments above on DR and Fig. 1 legend - the same points apply.

4) Conjugation experiments Lines 118-123, 319-331
NEW COMMENT: Lines 118-120 - pSAL22057-NDM has not been identified or named at this point and the wording is unclear and repetitive. Presumably the transconjugants were checked by PCR for blaNDM-13 and blaOXA-10 and only blaNDM-13 was detected? See comments above about inconsistencies in MICs. Line 120 - wording doesn't make sense.
It might also be good to look for conjugation of pSAL22057-NDM but different selection conditions would be needed (not necessarily for blaOXA-10, as one of the other resistance genes on the plasmid could be selected for) and noting that HI2 plasmid transfer better at temperatures around 25-30 degrees, rather than 37.
ORIGINAL COMMENT: Lines 303-4 - more details are needed. Liquid or solid mating, what media, what donor:recipient ratio, time or conjugation? Or cite a suitable reference.
AUTHORS' RESPONSE: We have added more details in the revised manuscript and cite a suitable reference (line319-320).
1.Boralli CMDS, et al. Characterization of blaKPC-2 and blaNDM-1 plasmids of a K. pneumoniae ST11 outbreak clone. Antibiotics (Basel) 12, 926 (2023).
NEW COMMENT: The comment said either give the details or cite a reference - doing both is not really necessary - and the details of the methods added do not match Boralli et al., e.g., they used J53 as recipient and selected with azide, not C600 with selection with rifampicin, incubation times, volumes and media differ. Boralli is Ref 24 and is not cited on Lines 319-20, but on Line 289 in relation to DNA preparation. Ref 30 cited here is Alijanabi et al, which is about DNA extraction i.e. they seem to have been cited the wrong way round, but Ref 24 is not needed.
ORIGINAL COMMENT: Line 314 - this needs a reference
Authors' response: We have added a reference Ref 30.
NEW COMMENT: Line 314 in the original manuscripts corresponds to Line 289 in the revised manuscript, which cites Ref 24 - see comment above.

5) Discussion
NEW COMMENT: Parts of the Discussion still repeats Results and it would need to be rewritten after changes to the rest of the manuscript.
ORIGINAL COMMENT: Line 171-2 - are all ST29 S. Stanley?
AUTHORS' RESPONSE: No, one meropenem-resistant isolate was identified as Salmonella Stanley ST29.
NEW COMMENT: Sorry if this was not clear, but I was asking if ST29 includes other serotypes and/or if all S. Stanley isolates are part of ST29?
NEW COMMENTS
Lines 188-9 - this should be blaNDM and the allele number(s) should be specified, "(IncA/C/HI2)" is confusing - if you mean that the blaNDM gene was on an A/C-type plasmid in one S. Stanley isolate and on an HI2 plasmid in the other then this needs to be made clearer and IncA/C should not be used - see PMID: 29486211 - most A/C type plasmids are IncC.
Lines 224-5 - what is the evidence for this?
Lines 239-253 - the role of IS26 is rearranging resistance region is already well-known and "consistence presence" IS26 copies flanking blaOXA-10 in these three plasmids does not really demonstrate anything, as the plasmids are very highly related over the while resistance region and beyond. Identification of the same structure in other locations would be needed.

6) Methods
ORIGINAL COMMENT: Lines 96-8 - I couldn't find blaTEM-60 on the chromosome and only found blaTEM-1b, not blaTEM-95, on pSAL22057-NDM-13 and these genes didn't come up in a ResFinder analysis - this needs checking.
AUTHORS' RESPONSE: This study systematically re-analyzed antimicrobial resistance gene annotations through a multi-database cross-validation strategy, integrating ResFinder v4.7.2 (based on core genome alignment) with CARD v3.2.5 (employing a strict domain validation model). Furthermore, phenotype-genotype association discrepancies in the initial draft were calibrated according to the clinical breakpoint standards (CLSI 2023). Through multi-database validation, only the TEM-1B gene was definitively identified in this strain. We made corrections in the revised manuscript (line135-136), Table-1.
NEW COMMENT: This is not properly described in the Methods section. Using NCBI AMRFinderPlus (https://www.ncbi.nlm.nih.gov/pathogens/antimicrobial-resistance/AMRFinder/) might have been an easiest way to check the alleles.
ORIGINAL COMMENT: Lines 298-9 - would these primers detect all variants of blaIMP and blaVIM? The blaOXA group in particular is very broad and includes many subgroups - which types would be detected by the primers used? blaOXA-48-like? Ref 23 needs to be checked and this information stated correctly.
AUTHORS' RESPONSE: These primers can't detect all variants of blaIMP and blaVIM. Yes, it is blaOXA-48-like (line283-284). We used these primers to screen for common carbapenemase genes (blaNDM, blaKPC, blaVIM, blaIM, and blaOXA-48-like),

with definitive confirmation of detected genes established through whole-genome sequencing results. We have checked Ref 23 and updated it to a new source.
NEW COMMENT: The text needs to state which genes would be detected by the primers used.
ORIGINAL COMMENT: Line 311 - what is meant by "conjugation elements"?
AUTHORS' RESPONSE: We rephased "conjugation elements" as "bacterial mobile genetic elements".
NEW COMMENT: Lines 330-331 - in addition to plasmids, the term "mobile genetic elements" encompasses IS and transposons, which would not be detected by oriTfinder, so is incorrect here.
Lines 371-4 - the sequences in GenBank are not annotated. It would be helpful to add annotations.
AUTHORS' RESPONSE: Although the sequences in GenBank have been annotated, the SAL22057 chromosome sequence and its features are not displayed in NCBI due to its large size.
NEW COMMENT: The versions of the plasmid sequences currently in GenBank are still not annotated. Maybe they are waiting to be updated? If not, they should be annotated.

7) NEW COMMENT: Section 2.2, Lines 124-151, plus 152-7, 171-2
Some reorganizing here would allow the text to be condensed. This section could just focus on the chromosome, SPI's (put this part before plasmid descriptions?) and basic characteristics of plasmids with most information about pSAL22057-NDM and pSAL22057-OXA moved to their specific sections to avoid repetition. It seems that pSAL22057-NDM carries only blaNDM-13 and pSAL22057-OXA carries all other resistance genes except aac(6´)-Iaa, which should be made clear, and aac(6´)-Iaa is known to be an intrinsic chromosomal gene in Salmonella, which also needs to be stated.
Lines 139-40 would be better at the start of the paragraph when the chromosome is being discussed.
Lines 133-4 - there should not be a space after aac or aph in these gene names.
Line 135, 137 - the gene downstream of blaNDM genes is bleMBL, not blaMBL.

8) NEW MINOR/WORDING ETC COMMENTS
Line 34-5 - the wording here still does not make sense - "genomic characterization" is not " analysed ".
Line 40 - this should be IncI1alpha, not IncI1Ialpha
Line 55 - "is ST29" or "belongs to ST29" here is better than "affiliated with ST29".
Line 64 - "OXA-type" is incorrect here - at most it should only be "OXA-10-type".
Lines 78-9 - the wording here needs to be made more accurate and more grammatically correct. It should be "the blaKPC, ... or blaOXA-48-like genes", but "and/or" should be used instead of "or" if any have examples have >1 of these genes, and "like" should not be subscript.
Line 82 - words missing.
Line 111 - lowercase I for imipenem
Line 126 - this size for pSAL22057-OXA is wrong - it should be "237,376 bp" and this formatting should also be used for the other numbers i.e. there should be a space between numbers and units and for and no spaces around the comma.
Lines 133-5 - are all resistance genes in the isolates listed here? If so, "included" here is incorrect and must be removed.
Line 136 - "the plasmid" is not needed here - the "p" in the name indicates this. Also check and remove/reword throughout e.g. Lines 160, 161, figure legends.
Lines 125-40 - this could be condensed by stating the plasmid sizes and the resistance genes that they carry.
Lines 152-3 - this an incomplete sentence that does not make sense and does not seem necessary.
Lines 153, 157, 171 - CP162097 is now listed on both lines 153 and 157 and CP195667 on both Lines 157 and 171. Each only needs to be given once.
Line 155 - this plasmid does not carry blaOXA-10.
Line 156 - "The carbapenem resistance gene" is not needed here.
Lines 157, 172- what is the relevance of overall GC content? Particularly for the blaOXA-10 plasmid, that appears to have many different insertions that could have different GC contents.
Line 182 - wording does not make sense.
Line 187 - the title of Ref 10 says "identified in the US". The isolate was from a patient from India.
Line 191 - "designated as" is not needed here.
Line 208: "describeded the first report" needs fixing.
Line 214- "Escherichia coli should be in italics"
Line 219 - "it is worth noting that" "are classified", "is carried", as all of these things are still true.
Lines 222-3 - blaNDM-13 was transferred from SAL22057 to C600, or transferred on pSAL22057-NDM, not from pSAL22057-NDM.
Lines 224-5 - what is the evidence that suggests this?
Line 261 - "carrying"
Line 270 - suggest rewording "isolate ... was isolated"
Lines 314-5 - wording does not make sense.
Line 319 - "Overnight cultures", "recipient" not "receptor"
Line 323 - something has gone wrong here.
Line 333 - how were they chosen?
References : There are still problems with missing details, journal names not correctly abbreviated etc and some citations seem to have been mixed up (see comments above).
Table 1 - in addition to inconsistencies between MICs in this table and text and the Table 1 in the original manuscript (see

comment 1 above), CXT should be CTX (see Line 110), IMP is normally abbreviated IPM (also Line 111) and AZM (usual abbreviation for azithromycin) should presumably be ATM, as on Line 113.

Reviewer #2 (Comments for the Author):

All my concerns have been addressed. No further comments.

Reviewer #4 (Comments for the Author):

The case (pediatric S. enterica serovar Stanley, ST29, co-harboring blaNDM-13 and blaOXA-10 on distinct plasmids) is noteworthy, but claims and evidence need tightening.
Major comments
1.2 Conjugation experiments are incomplete. Only blaNDM-13 transfer was shown. Test/ report transfer of the IncHI2/HI2A OXA-10 plasmid (optimize temperature, duration), and quantify frequencies; confirm structures in transconjugants.
1.3IS-mediated mechanism claims are over-stated. Map IS boundaries (IR/DR), show long-read evidence spanning events, and present integron/cassette architecture; downgrade language to "likely/consistent with."
3. Phylogenetic/epidemiologic context is thin. Add cgMLST/ core-genome tree and geographic/host distribution of related ST29 and homologous IncI1-NDM-13 / IncHI2-OXA-10 plasmids to support public-health relevance.
4. Genotype-phenotype alignment needs clarity. Explain aztreonam susceptibility under the recorded β-lactamase background; if possible, add AZT-AVI or CAZ-AVI{plus minus}AZT synergy data to enhance translational value.

Minor comments
1. Systematic copy-editing (spelling, grammar, units, spacing, "33rd ed." for CLSI, consistent tense and abbreviations).
2. Clarify MIC methodology, replicates, media, and incubation details; clean up typos in protocols.
3. Improve readability (directionality, boundary markers, scale bars, clear color keys); correct phrasing ("closely related," not "closed").
4. Add columns for replicate counts and fold-change before/after transfer; ensure totals and spacing are correct.

The case (pediatric *S. enterica* serovar Stanley, ST29, co-harboring blaNDM-13 and blaOXA-10 on distinct plasmids) is noteworthy, but claims and evidence need tightening.

Major comments

1. Conjugation experiments are incomplete. Only *bla*$_{NDM-13}$ transfer was shown. Test/ report transfer of the IncHI2/HI2A OXA-10 plasmid (optimize temperature, duration), and quantify frequencies; confirm structures in transconjugants.

2. IS-mediated mechanism claims are over-stated. Map IS boundaries (IR/DR), show long-read evidence spanning events, and present integron/cassette architecture; downgrade language to "likely/consistent with."

3. Phylogenetic/epidemiologic context is thin. Add cgMLST/ core-genome tree and geographic/host distribution of related ST29 and homologous IncI1-NDM-13 / IncHI2-OXA-10 plasmids to support public-health relevance.

4. Genotype–phenotype alignment needs clarity. Explain aztreonam susceptibility under the recorded β-lactamase background; if possible, add AZT-AVI or CAZ-AVI±AZT synergy data to enhance translational value.

Minor comments

1. Systematic copy-editing (spelling, grammar, units, spacing, "33rd ed." for CLSI, consistent tense and abbreviations).

2. Clarify MIC methodology, replicates, media, and incubation details; clean up typos in protocols.

3. Improve readability (directionality, boundary markers, scale bars, clear color keys); correct phrasing ("closely related," not "closed").

4. Add columns for replicate counts and fold-change before/after transfer; ensure totals and spacing are correct.

1.This revised manuscript describes analysis of a Salmonella Stanley isolate that carries blaNDM-13 and other resistance genes, including blaOXA-10. The original version stated that blaNDM-13 and blaOXA-10 were on the same large plasmid with HI2, HI2A and I1alpha replicons. After checking assemblies, the authors have now concluded that that blaNDM-13 is on an I1 plasmid and blaOXA-10 on an HI2 plasmid, so this revised version has substantial differences from the original.

The authors have also included further justification for the focus on blaOXA-10, but it seems unlikely that this gene to contributes to carbapenem resistance in this isolate and no direct evidence is presented relating to this, so I believe that this aspect should not be a main focus of the manuscript.

Closer attention to detail is needed to avoid simple errors and the wording/English still needs attention in places. To reduce the number of comments, I have tried to group those that relate to the same point and have not included original comments that were adequately addressed.

Authors' response: Thank you for your thorough review of our revised manuscript and for your valuable feedback. We appreciate the time and effort you have dedicated to evaluating our work. We are pleased that the major restructuring regarding the plasmid locations of *bla*$_{\text{NDM-13}}$ and *bla*$_{\text{OXA-10}}$ is acknowledged. As you highlighted, the revised version now accurately reflects that *bla*$_{\text{NDM-13}}$ and *bla*$_{\text{OXA-10}}$ are located on separate plasmids (1α and HI2, respectively), a conclusion reached after careful re-analysis of our assemblies. This indeed constitutes a substantial and important change from the original submission, and we have ensured the manuscript text and

figures are updated accordingly. The role of *bla*$_{OXA-10}$ have been de-emphasized. It was discussed only as part of the overall resistance gene profile without implying a primary role in carbapenem resistance. We have performed a careful proofreading to correct errors and improve clarity throughout the manuscript.

1) Focus on blaOXA-10 as a "carbapenemase"

1.ORIGINAL COMMENT: *bla*$_{OXA-10}$ is also mentioned in several places but as this gene is not considered to encode a carbapenemase the reason for focusing on it is unclear (many other AMR genes are also present).

AUTHORS' RESPONSE: When expressed in Acinetobacter baumannii ATCC 17978, OXA-10 conferred high-level resistance to carbapenems, with MIC values for meropenem, ertapenem, and doripenem comparable to those of recognized carbapenemases like OXA-48 and higher than OXA-58. For example, the MICs of meropenem, ertapenem, and doripenem were 16 μg/ml, 128 μg/ml, and 16 μg/ml, respectively, indicating clinical resistance levels. Although OXA-10 has low hydrolysis efficiency for carbapenems, it exhibits high substrate affinity (nanomolar dissociation constants), consistent with carbapenemase characteristics. In Acinetobacter baumannii (with low membrane permeability and efflux pumps), it showed significant resistance, a hallmark of carbapenemase. OXA-10 was expressed 25-fold more highly in Acinetobacter baumannii than OXA-48. High expression levels offset lower catalytic efficiency, leading to equivalent resistance. In summary, OXA-10 has both carbapenem-hydrolyzing ability and clinical resistance phenotypes, qualifying it as a carbapenemase1. OXA-10 and its variants (e.g., OXA-655) exhibit

carbapenem-hydrolyzing activity, host-dependent clinical resistance, and evolutionary plasticity, meeting the criteria for classification as carbapenemases2. Thus, OXA-10 showed to be weak carbapenemases. We added this part of the content in the revised manuscript (line 86-90).

1） Antunes NT et al. Class D β-lactamases: are they all carbapenemases? Antimicrob Agents Chemother 58, 2119-25(2014).

2） Kotsakis SD et al. Characterization of the First OXA-10 Natural Variant with Increased Carbapenemase Activity. Antimicrob Agents Chemother. 63, e01817-18(2018).

NEW COMMENTS: I still believe that the focus on OXA-10 as a carbapenemase and apparently suggesting that it is equivalent to NDM and other "classic" carbapenemases is not appropriate.

Ref 1 above states "Here we demonstrate that when OXA-2 and OXA-10 are expressed in Escherichia coli strain JM83, they produce a narrow-spectrum antibiotic resistance pattern. When the enzymes are expressed in A. baumannii ATCC 17978, however, they behave as extended-spectrum-lactamases and confer resistance to carbapenem antibiotics." The isolate being studied here is Salmonella, which is part of the Enterobacterales.

Ref 2 is about a specific variant, OXA-655, not OXA-10 itself. Of the 70 OXA-10 alleles listed in the NCBI Reference Gene Catalog (https://www.ncbi.nlm.nih.gov/pathogens/refgene/#OXA-10) only two (OXA-233 and blaOXA-655) are listed as having carbapenemase activity. blaOXA-655 in Ref 2

above was also found on an IncQ plasmid, which might have a high copy number contributing to greater resistance. Ref 2 also states "While carbapenem resistance in Gram-negative bacteria is mainly due to the production of efficient carbapenemases, beta-lactamases with a narrower spectrum may also contribute to resistance when combined with additional mechanisms. OXA-10-type class D-lactamases, previously shown to be weak carbapenemases, could represent such a case."

No evidence is presented here that that OXA-10 contributes to the carbapenem resistance phenotype in SAL22057, indeed Lines 115-6 state that "the presence of a metalloenzyme in SAL22057 explains the carbapenem resistant phenotype".

Authors' response: Thank you for your persistence on this critical point and for providing the compelling references and reasoning. Upon careful reconsideration, we agree that our emphasis on OXA-10 as a primary carbapenemase, equivalent to NDM, was misplaced and not supported by the evidence in our study or the broader literature context for *Enterobacterales*. All references to OXA-10 in the manuscript have been revised to align with the reviewer's accurate assessment. We now state that OXA-10 is primarily a narrow-spectrum β-lactamase and, crucially, that it is not universally classified as a carbapenemase in the same category as NDM, KPC, or OXA-48-like enzymes, especially in *Enterobacterales* like *Salmonella*. We acknowledge that its reported carbapenemase activity is weak, host-dependent (as in *A. baumannii*), and not representative of most alleles. We have strengthened the statement that NDM-13 is the unequivocal and primary determinant of the carbapenem-resistant phenotype in SAL22057, as correctly noted by the reviewer. Instead of framing OXA-10 as a

carbapenemase, we now present its presence as part of the broader multidrug-resistant (MDR) arsenal of this isolate (Lines 87-90). We have de-emphasized OXA-10's role regarding carbapenems and reframed its significance to be accurate and consistent with the literature and the reviewer's expert opinion.

2.The original manuscript might suggest a difference between the MEM MIC in SAL22057 (32) and the transconjugant (NDM-13 only, 16) but there are inconsistencies in the MICs of IPM and MEM between Table 1 in the original manuscript (SAL22057, IPM 16, MEM 32; transconjugant, IPM 16, MEM 16) and Table 1 in the revised manuscript (SAL22057, IPM >16, MEM >16; transconjugant, IPM >16, MEM >16) and between Table 1 and the text in the revised manuscript (Lines 37, 57 SAL22057 IPM 16, MEM 32, Lines 121-2 transconjugant IPM 16, MEM 16). This doesn't seem to be explained in the response, so which numbers are correct?

Authors' response: Thank you for your comment. We deeply appreciate you flagging this unacceptable discrepancy. We have now conducted a full audit of our raw data and the manuscript to resolve this issue. We have corrected the errors in Table 1. The correct MIC values, as confirmed by our original laboratory records, are as follows: 1. *S. Stanley* SAL22057: IPM 16 μg/ml; MEM 32 μg/ml. 2. SAL22057-*E. coli* 600: IPM 16 μg/ml; MEM 16 μg/ml. The values showing ">16" in a previous version of the revised Table 1 were incorrect and resulted from a clerical error during table formatting. The values in the original submission's table and the text (Lines 37-8, 58, 122-123) were, in fact, correct. **Table 1** has been updated to reflect the correct MICs

as stated above. We have performed a word-by-word verification of the entire Results section to ensure that all MIC values mentioned in the text exactly match the final, corrected Table 1.

3.To show any contribution of OXA-10 to carbapenem resistance in SAL22057 curing pSAL22057-NDM, leaving only pSAL22057-OXA would probably be needed. As the chromosome sequence is available it would be possible to look for chromosomal mutations that could contribute to carbapenem resistance. If authors want to suggest that blaOXA-10 in a mobile region on plasmid in Salmonella is concerning because this gene could spread/mutate and contribute to carbapenem resistance in this (and maybe other) species under certain conditions, then there needs to be more justification of the likely importance of this and it needs to be explained much more clearly and briefly as part of the Discussion only, and should not be the main focus of the manuscript.

Authors' response: Thank you for this constructive guidance. We fully agree that clarifying the individual roles of various resistance determinants is important. We would like to kindly clarify that the primary focus of our manuscript is the characterization of NDM-13 as the key carbapenemase in *Salmonell*a strain SAL22057. As such, the experimental narrative and discussion are centered on the role and context of NDM-13. While addressing these points experimentally is beyond the immediate scope of the current manuscript (which is focused on establishing the primary NDM-13 mechanism), we consider them to be important follow-up questions. We plan to incorporate these experiments into our ongoing and future research plans

to further elucidate the resistance landscape of this strain.

SPECIFIC POINTS RELATING TO COMMENTS ABOVE:

4.Title - should be updated after changes have been made.

Authors' response: Thank you for your valuable feedback regarding the focus and presentation of our findings. We appreciate your point that the original title could be misinterpreted as equating the roles of *bla*$_{NDM-13}$ and *bla*$_{OXA-10}$ in conferring carbapenem resistance in the *Salmonella* isolate. We have revised the title to more accurately reflect the study's conclusions. The updated title is: "Carbapenem Resistance Mediated by *bla*$_{NDM-13}$ and Co-occurrence of *bla*$_{OXA-10}$ in a *Salmonella* Stanley ST29 Strain in China". This modification explicitly states that NDM-13 is the primary mediator of the carbapenem-resistant phenotype, while the presence of *bla*$_{OXA-10}$ is reported as a significant co-occurring genetic feature. We have also ensured the manuscript text consistently supports this clarification, particularly in the abstract (Lines 33-4) and discussion (Lines 257-262) sections. We believe this revised title addresses your concern by eliminating potential misunderstanding and more precisely representing our results

5.Line 54 - I don't think that it can be argued that blaOXA-10 is "critical" in SAL22057.

Authors' response: Thank you for this correction. We agree with your assessment. The use of the term "critical" to describe *bla*$_{OXA-10}$ in this specific context is not accurate, as our own data and conclusions establish that *bla*$_{NDM-13}$ is the primary and critical determinant for the carbapenem resistance in strain SAL22057. In the revised

manuscript, we have amended the sentence in question to remove the subjective and overstated descriptor. The text now reads: "This study identified a carbapenem-resistant *Salmonella* Stanley strain, SAL22057, from a pediatric patient, which co-harbored the carbapenemase gene bla_{NDM-13} and bla_{OXA-10} *β*-lactamase gene."

6.Lines 59-60 "dual carbapenemase" is not an appropriate description.

Authors' response: Thank you for this correction and agree that the term "dual-carbapenemase mechanism" was inaccurate and overstated. The inappropriate phrasing has been removed from the revised manuscript.

7.Lines 86-90 - need rewriting to be more accurate and reflect comments above.

Authors' response: Thank you for your comment. In the revised manuscript, we have removed the broad claim that OXA "qualifies as a carbapenemase" in this context. Instead, we have rewritten this section to provide a more accurate and balanced perspective, incorporating the key distinction you highlighted (Lines 87-90). The revised text now reads: "In addition, class D β-lactamases such as OXA-10, while primarily conveying resistance to penicillins and early cephalosporins, have also been reported to exhibit weak carbapenem-hydrolyzing ability under certain conditions."

8.Lines 90-2 - this should be deleted, as the relevance of isolates with two "classic" carbapenemases to thsi manuscript is not clear.

Authors' response: Thank you for your comment. We have deleted the relevant content.

9.Lines 165-6 - blaOXA-10 is not considered a "significant carbapenemase gene".

How do you know that the plasmid is "highly well-adapted" and this study only mentions a single plasmid.

Authors’ response: Thank you for this correction. We agree that describing the strain as “adaptable” in this context is a subjective claim not directly supported by the experimental data presented in our study. We have therefore deleted the sentence in question from the revised manuscript to ensure all conclusions remain strictly evidence-based.

10.Lines 194-5 - needs further explanation and/or toning down. What is the "strain" being referred to and what is the evidence that is adaptable and transmissible?

Authors’ response: Thank you for your comment. We have removed statements that express ambiguity in revised text to make it clearer.

11.Lines 205-8 - this is not relevant. The multiple carbapenemases being referred here to are "classic" carbapenemases.

Authors’ response: Thank you for your comment. We have deleted the relevant content.

12.Lines 209-11 - this is not a complete sentence and is inaccurate, as OXA-10-like enzymes only gives reduced susceptibility under certain conditions (e.g. in Acinetobacter, if they have a specific mutation) and are 16 and 17 the correct references here, or should it be 6 and 7?

Authors’ response: Thank you for highlighting these important issues with the sentence structure, accuracy, and referencing. We have rewritten and completed the sentence, grounded it in the appropriate context, and corrected the citations (Lines

255-9). The revised text now reads: "While some studies have reported very low-level hydrolysis of carbapenems in vitro for OXA-10 and certain variants, this activity is considered weak[6,7] and is not its primary function. Therefore, OXA-10 is not universally classified as a carbapenemase in the same category as NDM, KPC, or OXA-48-like enzymes."

2) pSAL22057-NDM, Lines 152-169, including role of IS1294, Lines 42-3, 61-2, 163-9, 227-238, 259-60

13. ORIGINAL COMMENT: Lines 242,247-52 - this should refer to IS1294, not "the IS1294 transposase" that it contains. IS1294 is member of the IS91 family of rolling circle elements that are known to be able to capture and move adjacent sequences. This should be looked at properly to see if it could help to explain generation of pSAL22057-NDM-13 but see comments above about possible assembly issues.

AUTHORS' RESPONSE: We rephased "the IS1294 transposase" as "IS1294". Yes, in this study, ISAba125 truncated by IS1294 was found in blaNDM-13 harboring plasmids including pSAL22057-NDM. We thus suspected that IS1294 may be involved in the mobilization and dissemination of blaNDM-13. (line233-236).

NEW COMMENT: As stated in the original comment, IS1294 is a member of the IS91 family, rolling circle elements that are known to be able to move adjacent regions (see PMID: 10873528). It should be possible to tell fairly confidently if IS1294 was responsible for inserting part of Tn125 into the pSAL22057-NDM, so this needs to be looked at properly. On a quick look, it seems like MN219406 and CP092912 have a clear insertion vs. CP037994, while pSAL22057-NDM has a

deletion in this region. If there are papers associated with MN219406, CP092912 and/or CP037994 then these should be examined for any mention of an IS1294 mediated event and they should be cited.

Authors' response: Thank you for your comment. In response, we have included the corresponding region of the plasmid pgs_wt5 in the genetic context comparison the *bla*$_{NDM-13}$ gene cluster in Figure 1, and have revised the discussion accordingly in the manuscript (Lines 283-301).

Among the sequences MN219406, CP092912 and CP037994, were only able to identify a reference CP092912: "Emergence of a Salmonella Rissen ST469 clinical isolate carrying *bla*$_{NDM-13}$ in China" (doi:10.3389/fcimb.2022.936649). Unfortunately, the authors did not provide a detailed analysis of the role of IS*1294* in this context. However, they noted that "IS*3000*, IS*26*, and IS*5* have also been reported to be associated with dissemination of NDM-encoding genes, while the role of IS*1294* is still unclear," and suggested that "We thus suspected that IS*1294* may be involved in the mobilization and dissemination of *bla*$_{NDM-13}$"

Below, we summarize our understanding of *bla*$_{NDM-13}$ region based on the findings of this study. Comparative visualization of the insertion of IS*1294* upstream of IS*Aba125* resulted in the truncation of the latter, retaining only a 44-bp fragment (positions 4612-4655) corresponding to the 3'-end of the *tnpA* gene. The *terIS* region of IS*1294* (4586-4611 bp) is located directly adjacent to the truncated ΔIS*Aba125*. Upon integration upstream of IS*Aba125*, IS*1294* serves as a key element driving the mobilization of the "IS*1294*–*bla*$_{NDM-13}$ unit" The plasmid pgs_wt5 appears to

represent the ancestral state of such plasmids before the acquisition of this unit. In both pNDM13-SR33 and pHNAHS65I-1, the "IS*1294–bla*$_{NDM-13}$ unit" is inserted into a region encoding a hypothetical protein located upstream of the *proQ* gene. This insertion splits the hypothetical protein into two segments, which we refer to here as hp-left and hp-right for clarity. The hp-left segment is 241 bp in length. Sequence analysis reveals that in both pNDM13-SR33 and pHNAHS65I-1, this segment (positions 4713–4953) is directly adjacent to a *terIS* region (4,954–5,053 bp), with no nucleotides in between. The hp-right segment is 819 bp long and shares a 25-bp repeat with the 3′ end of the *trpF* gene. Based on this, we speculate that the integration of the "IS*1294–bla*$_{NDM-13}$ unit" may have involved homologous recombination with this hypothetical protein. These findings support the notion that the "IS*1294–bla*$_{NDM-13}$ unit" exhibits a specific insertion preference in such plasmids, and that the 25-bp homologous sequence likely plays an important role in determining its integration site.

A distinctive feature of the plasmid pSAL22057-NDM reported in this study is that, compared to the reference plasmids pNDM13-SR33 and pHNAHS65I-1, the insertion of the "IS*1294–bla*$_{NDM-13}$ unit" resulted in the deletion of hp-left and its upstream region, including proQ and two additional hypothetical proteins—amounting to the loss of four ORFs in total. This observation further underscores the molecular diversity and genomic complexity associated with the integration mechanisms of the "IS*1294–bla*$_{NDM-13}$ unit."

14.ORIGINAL COMMENT: Lines 112-3 - IncI plasmids have a shufflon that may not

be correctly assembled, even from long read data (see PMID: 28424528). This needs to be looked at and may explain some differences from other IncI1 plasmids.

NEW COMMENT: There was no specific response to this.

Authors' response: Thank you for this follow-up and pointing out that our previous response did not specifically address the important issue regarding the potential misassembly of the shufflon region in IncI-type plasmids, as noted in the referenced literature (PMID: 28424528). To specifically address your concern, we performed the following additional analysis on the pSAL22057-NDM (IncI1α) plasmid: We manually inspected the assembly graph and raw long-read alignments across the putative shufflon region. We confirmed that the assembly in this region is supported by continuous, high-quality long reads with consistent alignment and no breakpoints indicative of a collapse or misassembly. While the repetitive and recombinogenic nature of shufflon loci is well-documented and can pose challenges, our analysis based on the current data suggests a contiguous and consistent assembly for the specific allele present in our strain. We have now added a brief statement in the revised Results (Section 2.2) and Methods (Section 5.4) to acknowledge this known complexity and to state that our manual validation did not detect evidence of misassembly in this region. The assembly of this plasmid, including its shufflon configuration, is therefore presented as our best representation based on the available sequencing data.

OTHER NEW COMMENTS:

15. Line 162 - "pgs_wt5" does not appear to be a complete plasmid.

Authors’ response: Thank you for your comment. We confirm that 'pgs_wt5' corresponds to the complete plasmid pSG_wt5, as designated in the official GenBank record (accession NZ_CP037994). This sequence was resolved using PacBio RSII long-read technology, which robustly generates closed, circular plasmid genomes.

16. Line 233 - it's more than IRR - it's 51 bp of the IRR end. Does this segment include the promoter?

Authors’ response: Thank you for your insightful comment. You are correct in pointing out that the region in question comprises not only the IRR sequence but also an adjacent 51 bp segment from the end of the IRR, which indeed includes part of the promoter region. We acknowledge the inaccuracy in our initial description regarding the IRR boundary. We have now corrected this inaccuracy in the revised version of the manuscript, clarifying the precise sequence composition in this region. Furthermore, we have incorporated additional data from the newly added sample psg_wt5, which has led to a refined interpretation of the results (Lines 161-163). This includes a more detailed analysis of the oriIS/terIS region flanking IS*1294* and the truncated state of IS*Aba125* in this context. These updates and a more comprehensive discussion of the findings are provided in our response to your Question #13 within the point-by-point reply.

Figure 1

17. NEW COMMENT: I would still suggest that Part A be moved to supplementary. There seems to be something wrong with the top of Part B, with part of the text moved to the right. The equivalent region of pgs_wt5 should also be shown (see

comments above). DR are shown in the key, but no DR seem to be shown in the diagrams?

Authors' response: Thank you for your valuable suggestion. The figure panels have been updated: Part A has been repositioned as Figure S1 in the Supplementary Materials, and Part B now constitutes Figure 1. Figure 2: Part A has been repositioned as Figure S2 in the Supplementary Materials, and Part B now constitutes Figure 2

This has been addressed, and the figure is now correctly aligned. The text misalignment in Part B was a typesetting error during the figure preparation. We have now fixed this issue and carefully checked all other figures to ensure proper formatting. The corresponding region of plasmid pgs_wt5 has been added to Figure 1 (see response to Question 13). Additionally, the label for direct repeats (DRs) has been removed from the figure, as our re-analysis verified their absence in the specified region of pSAL22057-NDM.

Legend

18. Lines 437-8 - wording does not make sense. "Comparison" not "Characterization"? "closely" not "closed".

Authors' response: Thank you for your comment. "Characterization" has been revised to "Comparison", and "closed" has been adjusted to "closedly" as suggested.

19. Lines 439, 442-3 - there is no need to give each accession no. twice.

Authors' response: Thank you for your comment. We have removed this recurrent section in the revised manuscript.

20. Line 445 - "resistance genes" is normally used, "Inserion sequences" needs

correcting, or just use "IS"

Authors' response: "Resistance genes" has been consistently used throughout the revised manuscript, and "Inserion sequences" has been adjusted to "IS".

3) pSAL22057-OXA with blaOXA-10 and other resistance genes.

21. NEW COMMENTS: A much clearer/more accurate description of the resistance regions of pSAL22057-OXA is still needed e.g., Line 176 still refers to "the region", which does not make sense, and "integron 3" (Line 181) is unclear without further explanation. The AMR genes currently listed on Lines 133-5 could be stated here instead, indicating which are in the same cassette array and which others are nearby. Could any IS/Tn explain insertions, are there any DR etc. Again, are there any papers associated with the related plasmids listed in this section that might already have information on this that could be consulted and cited. Information on Lines 245-8 need to be in Results not Discussion.

Authors' response: Thank you for your comment. The description of the pSAL22057-OXA resistance regions has been amended for greater accuracy and clarity (Lines 195-202). The revisions include a precise explanation of "Integron 3" and a delineation of the resistance gene distribution, specifying their arrangement either within the integron's cassette array or in flanking sequences (Lines 198-200). Furthermore, it is confirmed that no direct repeats (DRs) are associated with this structure.

22. ORIGINAL COMMENT: Lines 128-9 - the information about IS640 isn't relevant to this manuscript. It would be more useful to look for flanking direct repeats, as these

would give evidence of a simple insertion, and state if these are present (also Lines 135-6).

AUTHORS' RESPONSE: We have removed the information about IS640 in the revised manuscript. We have identified the inverse and direct repeats within in the region of blaNDM-13 (pSAL22057-NDM) and blaOXA-10 (pSAL22057-OXA), and these have been annotated in the updated Figure 1B/2B. For further information, please consult Table S3, S4. IS26 features two flanking 8-base pair direct repeats (DRs): TCGCAGGA is adjacent to the left inverted repeat (IRL), and CTTTTTTG is adjacent to the right inverted repeat (IRR).

NEW COMMENT Lines 183-5: It is not clear from the text which IS26 is being referred to here, but the "DR" shown on Fig. 2 seem to be flanking the IS26 downstream of dfrA14. The 8 bp sequences immediately flanking this copy of IS26 are CGCAGGAC (not TCGCAGGA) and CTTTTTTG but these are not DR - the name "direct repeats" is because they have the same sequence. As these "DR" are incorrect, and the ends of a particular IS are always the same, Tables S3 and S4 do not include any useful information and should be removed.

Authors' response: You are correct. The Inverted Repeat (IR) of IS*26* should indeed be 14 bp (5'→3': GGCACTGTTGCAAA). Our initial error stemmed from misinterpreting information from ISfinder, where we inadvertently included the conserved sequence between the IR and the transposase as part of the IR itself. This has now been corrected in the table. Regarding the DRs, we had initially annotated potential DRs based on existing reports in ISfinder. However, as we could not identify

definitive DR sequences flanking the IR. The DR annotations have been removed from both Figure 1 and 2. We thank you for this valuable correction.

23. ORIGINAL COMMENT: Line 210 - does pSAL22057-NDM-13 have full conjugation regions of both HI2 and I1 plasmids?

AUTHORS' RESPONSE: Based on the re-assembly, pSAL22057-NDM have full conjugation regions of I1Iα plasmid.

NEW COMMENT: What about the conjugation region of pSAL22057-OXA?

Authors' response: A variety of conjugation-associated modules were identified in pSAL22057-OXA, including *oriT*, Relaxose, T4CP, and T4SS family genes. We have added this content in Line 182-183 in revised manuscript.

24. ORIGINAL COMMENT: Line 262 - "TnAs3 tnpR" matches Tn21, which is a well-known Tn that carries an integron, so it would be better to use this name (also on Figures).

AUTHORS' RESPONSE: Based on the re-assembly, we removed this inappropriate expression.

NEW COMMENT: "TnAs3-tnpA" is still shown on Fig. 2, so needs to be corrected/explained.

Authors' response: Thank you for your comment. We have revised the labels in Figure 2 and S2 to Tn21-tnpA and Tn21-tnpR, respectively.

25. ORIGINAL COMMENT: Lines 267-70 - which plasmid has the inverted region with blaNDM-5 that is mentioned? Is there any clue about how this inversion might have occurred? Why would blaNDM-5 be acquired and by what?

AUTHORS' RESPONSE: Based on the re-assembly, we removed this inappropriate expression.

NEW COMMENT: Inverted regions are shown in Fig. 2 still and need to be explained.

Authors' response: The inverted region of pSAL22057-OXA has been amended (Lines 202-203).

OTHER NEW COMMENTS:

26. Lines 41, 130, 171 - HI2 and HI2A are separate replicons, typically found together on an HI2 plasmid, and this needs to be correctly written throughout the manuscript.

Authors' response: Thank you for your comment. We have revised the above content in accordance your instructions.

27. Lines 176-9 - why mention the "functionally uncharacterized proteins"? The different IS and Tn should be properly identified.

Authors' response: Thank you for your comment. We removed the “functionally uncharacterized proteins”. The different IS and Tn have been properly identified (Figure 1 and 2).

28. Line 179 - homology is not quantifiable. Two things are either homologous or not. Do you mean high sequence identity? And wouldn't "an equivalent segment" always be closely related?

Authors' response: Thank you for your comment. The relevant section has been revised accordingly (Lines 188-190).

Figure 2

29. NEW COMMENT: Part A could be made supplementary. See comments above on DR and Fig. 1 legend - the same points apply.

Authors' response: Part A has been made supplementary Figure S2. It is confirmed that no direct repeats (DRs) are associated with this structure. The DR annotations have been removed from both Figure 2.

4) Conjugation experiments Lines 118-123, 319-331

30. NEW COMMENT: Lines 118-120 - pSAL22057-NDM has not been identified or named at this point and the wording is unclear and repetitive. Presumably the transconjugants were checked by PCR for blaNDM-13 and blaOXA-10 and only blaNDM-13 was detected? See comments above about inconsistencies in MICs. Line 120 - wording doesn't make sense.

Authors' response: Thank you for these specific and important corrections regarding the clarity and accuracy of our methodology description. We have revised the text to address all the points you raised (Lines 116-126). The updated section in the manuscript now reads: "Conjugation assay results demonstrated that the *bla*$_{NDM-13}$ was successfully transferred to the recipient *E. coli* C600. The conjugation frequencies were approximately 5.2×10^{-5} at 37°C, 1.4×10^{-4} at 30°C, and 2.3×10^{-4} at 35°C. Compared to 37 °C, the conjugation frequencies at 30 °C and 25 °C were approximately 2.7-fold and 4.4-fold higher, respectively. All *bla*$_{NDM-13}$-positive transconjugants obtained at temperatures exhibited resistance to both MEM and IPM, with consistent MICs of 16 μg/ml for both MEM and IPM. The MEM and IPM MICs of transconjugants increased at least 32-fold respectively, compared with the recipient

E. coli C600 (Table 1). These MICs were at least 64-fold higher than those of the plasmid-free recipient *E. coli* C600. While the *bla*$_{OXA-10}$ proved non-transferable to recipient *E. coli* C600 at 25°C, 30°C or 37°C."

31. It might also be good to look for conjugation of pSAL22057-NDM but different selection conditions would be needed (not necessarily for blaOXA-10, as one of the other resistance genes on the plasmid could be selected for) and noting that HI2 plasmid transfer better at temperatures around 25-30 degrees, rather than 37.

Authors' response: Thank you for the valuable suggestion regarding the optimization of the conjugation experiment. We have revised our methodology accordingly. In direct response to your comment, we have performed an additional, optimized conjugation experiment as follows: Temperature Adjustment: Based on the well-documented temperature sensitivity of many conjugative systems (including HI2-type plasmids), we repeated the conjugation assay at 25°C, 30°C and 37°C, for 18 hours each, with three independent replicates per condition. To specifically detect the transfer of the plasmid carrying *bla*$_{OXA-10}$ (pSAL22057-OXA), we employed a targeted selection strategy. Transconjugants were selected on Mueller-Hinton agar supplemented with rifampicin (200 mg/L, to counter select the donor) and CTX (2 mg/L, to select for the OXA-10 β-lactamase). The Methods 5.5 section (Lines 393-395) now described the optimized conjugation protocol. The relevant Results (Lines 116-26) and Discussion (Lines 274-78) section has been updated accordingly.

32.ORIGINAL COMMENT: Lines 303-4 - more details are needed. Liquid or solid mating, what media, what donor:recipient ratio, time or conjugation? Or cite a

suitable reference.

AUTHORS' RESPONSE: We have added more details in the revised manuscript and cite a suitable reference (line319-320).

1.Boralli CMDS, et al. Characterization of blaKPC-2 and blaNDM-1 plasmids of a K. pneumoniae ST11 outbreak clone. Antibiotics (Basel) 12, 926 (2023).

NEW COMMENT: The comment said either give the details or cite a reference - doing both is not really necessary - and the details of the methods added do not match Boralli et al., e.g., they used J53 as recipient and selected with azide, not C600 with selection with rifampicin, incubation times, volumes and media differ. Boralli is Ref 24 and is not cited on Lines 319-20, but on Line 289 in relation to DNA preparation. Ref 30 cited here is Alijanabi et al, which is about DNA extraction i.e. they seem to have been cited the wrong way round, but Ref 24 is not needed.

Authors' response:

Thank you for your valuable comment and careful review. We fully agree with your suggestion and have made corresponding revisions to address the issues raised: As recommended, we have removed the unnecessary citation of Boralli et al. (Ref 24) from the conjugation assay section. Since the experimental details (e.g., recipient strain *E. coli* C600, selection with rifampicin and meropenem, incubation parameters, and culture volumes) in our study are distinct from those in Boralli et al., we have retained the comprehensive method details to ensure transparency, while eliminating redundant citations that do not align with the conjugation protocol. Ref 30 (Alijanabi et al.) is now exclusively cited in the DNA preparation section (Line 358), where it is

relevant to the context without misplacement. To reconfirm, the key details of the conjugation assay retained in the revised manuscript are specific to our study (Line 390-402): liquid mating was performed in 5 mL fresh LB medium, with a donor: recipient ratio of 1:1, and incubation in parallel at three different temperatures: 25, 30 and 37°C, for 18 hours each. These details accurately reflect the experimental design and ensure reproducibility. We appreciate your guidance in refining the manuscript. The revisions have streamlined the method section and corrected citation inconsistencies, enhancing the clarity and rigor of our work. Please let us know if further adjustments are needed.

33. ORIGINAL COMMENT: Line 314 - this needs a reference

Authors' response: We have added a reference Ref 30.

NEW COMMENT: Line 314 in the original manuscripts corresponds to Line 289 in the revised manuscript, which cites Ref 24 - see comment above.

Authors' response: We have reorganized the references. Line 314 in the original manuscript corresponds to Lines 357–8 in the revised manuscript, which cites Ref. 22.

5) Discussion

34. NEW COMMENT: Parts of the Discussion still repeats Results and it would need to be rewritten after changes to the rest of the manuscript.

Authors' response: Thank you for this important feedback. We agree that an effective Discussion should interpret and contextualize the findings rather than reiterate the Results. In revising the manuscript based on all previous comments, we have thoroughly rewritten the Discussion section (Lines 232-7, 241-3, 253-9, 264-6,

275-81，284-317) to address this specific point.

35. ORIGINAL COMMENT: Line 171-2 - are all ST29 S. Stanley?

AUTHORS' RESPONSE: No, one meropenem-resistant isolate was identified as Salmonella Stanley ST29.

NEW COMMENT: Sorry if this was not clear, but I was asking if ST29 includes other serotypes and/or if all S. Stanley isolates are part of ST29?

Authors' response: Thank you for the clarification. We apologize for any confusion in our previous response. No, ST29 does not exclusively contain the Stanley serotype. Multi-Locus Sequence Type (ST) is based on housekeeping gene alleles and is independent of serotype classification. A single ST can, and often does, include multiple serotypes, especially in highly diverse species like Salmonella enterica. Conversely, the Stanley serotype is not confined to a single ST. While a specific serotype may be predominantly associated with a particular ST (a common clone), it can also be found in other STs due to evolutionary divergence or horizontal gene transfer of antigen-encoding genes. According to the primary Salmonella MLST database (enterobase.warwick.ac.uk), the vast majority of *S.* Stanley isolates belong to ST29, making ST29 the predominant and epidemic clone for this serotype. However, a minority of *S.* Stanley isolates have been assigned to other, less common STs (e.g., ST2152). Therefore, the statement in our manuscript has been revised for precision.

The ST29 Salmonella isolated from this slaughterhouse was also identified as the Stanley serotype, which is the serotype most frequently associated with the epidemic ST29 clone.

NEW COMMENTS

36. Lines 188-9 - this should be blaNDM and the allele number(s) should be specified, "(IncA/C/HI2)" is confusing - if you mean that the blaNDM gene was on an A/C-type plasmid in one S. Stanley isolate and on an HI2 plasmid in the other then this needs to be made clearer and IncA/C should not be used - see PMID: 29486211 - most A/C type plasmids are IncC.

Authors' response: Thank you for your valuable comments. We have revised the manuscript accordingly. We have changed "NDM" to *bla*$_{NDM}$ and specified the respective allele numbers, *bla*$_{NDM-1}$ and *bla*$_{NDM-5}$. We have clarified the distinct plasmid backgrounds. The revised text now explicitly states that the *bla*$_{NDM-1}$ gene was located on an IncC-type plasmid in the human-derived S. Stanley isolate (Huang et al., 2013, Emerg Infect Dis), while the *bla*$_{NDM-5}$ gene was carried on an IncHI2 plasmid in the slaughterhouse isolate from this study. We have replaced the outdated "IncA/C" designation with the correct term IncC, as recommended (PMID: 29486211) (Lines 233-7).

37. Lines 224-5 - what is the evidence for this?

Authors' response: Thank you for your valuable comment. To substantiate this claim, we have revised the discussion by citing recent, direct evidence from a "One Health" surveillance study. The updated text now reads (277-81): "Therefore, combined with the evidence of *bla*$_{NDM}$ transmission from animal-derived food to humans through epidemic plasmids (Fu B, *et al.*, 2024, Emerg Microbes Infect, PMID: 38629492), the *bla*$_{NDM-13}$-carrying plasmid identified in this study may represent a similar

dissemination risk within the food chain.” This revision incorporates direct molecular epidemiological support from the cited study, which documents the clonal and plasmid-mediated spread of *bla*$_{NDM}$ from the food chain to human isolates.

38. Lines 239-253 - the role of IS26 is rearranging resistance region is already well-known and "consistence presence" IS26 copies flanking blaOXA-10 in these three plasmids does not really demonstrate anything, as the plasmids are very highly related over the while resistance region and beyond. Identification of the same structure in other locations would be needed.

Authors’ response: Thank you for this valuable comment. We fully agree that the role of IS*26* in reorganizing resistance regions is well-documented, and that the conserved structure observed among these closely related plasmids may reflect shared ancestry rather than independent IS*26*-mediated events. Our revised discussion has been carefully rephrased to address this point (Lines 305-13). we have made the following adjustments in the manuscript: 1. We now explicitly state that the plasmids under comparison are highly related. 2. The focus has been shifted to describing the specific structural rearrangements (e.g., the inversion within Integron 2) observed in these plasmids, and we suggest these variations within this lineage were likely facilitated by the flanking IS*26* copies. 3. The concluding language has been moderated. Claims regarding a "broad and significant role" have been removed. Instead, we emphasize that the conserved IS*26*-*bla*$_{OXA-10}$ integron structure appears to be a stable, mobilisable unit within this related plasmid group.

6) Methods

39. ORIGINAL COMMENT: Lines 96-8 - I couldn't find blaTEM-60 on the chromosome and only found blaTEM-1b, not blaTEM-95, on pSAL22057-NDM-13 and these genes didn't come up in a ResFinder analysis - this needs checking.

AUTHORS' RESPONSE: This study systematically re-analyzed antimicrobial resistance gene annotations through a multi-database cross-validation strategy, integrating ResFinder v4.7.2 (based on core genome alignment) with CARD v3.2.5 (employing a strict domain validation model). Furthermore, phenotype-genotype association discrepancies in the initial draft were calibrated according to the clinical breakpoint standards (CLSI 2023). Through multi-database validation, only the TEM-1B gene was definitively identified in this strain. We made corrections in the revised manuscript (line135-136), Table-1.

NEW COMMENT: This is not properly described in the Methods section. Using NCBI AMRFinderPlus (https://www.ncbi.nlm.nih.gov/pathogens/antimicrobial-resistance/AMRFinder/) might have been an easiest way to check the alleles.

Authors' response: Thank you for your comment. Resistance genes were identified using AMRFinderPlus (https://www.ncbi.nlm.nih.gov/pathogens/antimicrobial-resistance /AMRFinder/) (Line 381-2).

40. ORIGINAL COMMENT: Lines 298-9 - would these primers detect all variants of blaIMP and blaVIM? The blaOXA group in particular is very broad and includes many subgroups - which types would be detected by the primers used? blaOXA-48-like? Ref 23 needs to be checked and this information stated correctly.

AUTHORS' RESPONSE: These primers can't detect all variants of blaIMP and

blaVIM. Yes, it is blaOXA-48-like (line283-284). We used these primers to screen for common carbapenemase genes (blaNDM, blaKPC, blaVIM, blaIM, and blaOXA-48-like), with definitive confirmation of detected genes established through whole-genome sequencing results. We have checked Ref 23 and updated it to a new source.

NEW COMMENT: The text needs to state which genes would be detected by the primers used.

Authors' response: The carbapenemase genes (*bla*$_{NDM}$, *bla*$_{KPC}$, *bla*$_{VIM-1}$, *bla*$_{IMP-4}$, and *bla*$_{OXA-48-like}$) were detected by the primers used. We have stated this in revised manuscript (352-3).

41. ORIGINAL COMMENT: Line 311 - what is meant by "conjugation elements"?

AUTHORS' RESPONSE: We rephased "conjugation elements" as "bacterial mobile genetic elements".

NEW COMMENT: Lines 330-331 - in addition to plasmids, the term "mobile genetic elements" encompasses IS and transposons, which would not be detected by oriTfinder, so is incorrect here.

Authors' response: We have rephased as "The conjugation probability of pSAL22057-NDM and pSAL22057-OXA were identified by the oriTfinder (https://bioinfo-mml. sjtu.edu.cn/oriTfinder/) (402-4).

42. Lines 371-4 - the sequences in GenBank are not annotated. It would be helpful to add annotations.

AUTHORS' RESPONSE: Although the sequences in GenBank have been annotated,

the SAL22057 chromosome sequence and its features are not displayed in NCBI due to its large size.

NEW COMMENT: The versions of the plasmid sequences currently in GenBank are still not annotated. Maybe they are waiting to be updated? If not, they should be annotated.

Authors' response: The plasmid sequences have been annotated. Please refer to the following link:

1.https://www.ncbi.nlm.nih.gov/nuccore/NZ_CP162099.2/

2.https://www.ncbi.nlm.nih.gov/nuccore/NZ_CP162098.2/

3.https://www.ncbi.nlm.nih.gov/nuccore/NZ_CP195668.1/

4.https://www.ncbi.nlm.nih.gov/nuccore/NZ_CP195669.1/

5.https://www.ncbi.nlm.nih.gov/nuccore/NZ_CP195667.1/

6.https://www.ncbi.nlm.nih.gov/nuccore/NZ_CP162097.2/

43. 7) NEW COMMENT: Section 2.2, Lines 124-151, plus 152-7, 171-2

Some reorganizing here would allow the text to be condensed. This section could just focus on the chromosome, SPI's (put this part before plasmid descriptions?) and basic characteristics of plasmids with most information about pSAL22057-NDM and pSAL22057-OXA moved to their specific sections to avoid repetition. It seems that pSAL22057-NDM carries only blaNDM-13 and pSAL22057-OXA carries all other resistance genes except aac(6′)-Iaa, which should be made clear, and aac(6′)-Iaa is known to be an intrinsic chromosomal gene in Salmonella, which also needs to be stated.

Authors' response: Thank you for this suggestion. We revised the section as follows (Lines 128-48):

1. **Reorganization for Logical Flow**: The description will be restructured to begin with the chromosomal characteristics (size, GC content, MLST ST29), followed by the identification of Salmonella Pathogenicity Islands (SPIs) and a summary of virulence genes. This establishes the core genomic context first.

2. **Clarification of Resistance Gene Localization**: The summary of plasmids and resistance genes will be rewritten for clarity. It will explicitly state that: Most of these resistance genes are located on pSAL22057-OXA. The $\textit{bla}_{\text{NDM-13}}$ and $\textit{ble}_{\text{MBL}}$ were located on a distinct plasmid, pSAL22057-NDM. Aac (6')-Iaa is an intrinsic chromosomal gene in Salmonella. The remaining four small plasmids carry no additional resistance genes.

3. As suggested, detailed analyses of pSAL22057-NDM and pSAL22057-OXA (including their sizes, GC content, replicon types, and in-depth genetic structure) will be moved to their dedicated results sub-sections later in the manuscript. The current section will only provide a concise overview.

44. Lines 139-40 would be better at the start of the paragraph when the chromosome is being discussed.

Authors' response: Thank you for this suggestion. We agree that placing the MLST result (ST29) at the beginning of the paragraph, when the chromosomal background of S. Stanley SAL22057 is first discussed, improves the logical flow. We have revised the manuscript accordingly (Lines 129-30), moving the sentence "Multilocus

sequence typing showed that S. Stanley SAL22057 belonged to ST29" to the start of the relevant paragraph.

45. Lines 133-4 - there should not be a space after aac or aph in these gene names.

Authors' response: We have removed the space after aac or aph in these gene names.

46. Line 135, 137 - the gene downstream of blaNDM genes is bleMBL, not blaMBL.

Authors' response: Thank you for your comment. We have rephased "$\textit{bla}_{\text{MBL}}$" as "$\textit{ble}_{\text{MBL}}$"

8) NEW MINOR/WORDING ETC COMMENTS

47. Line 34-5 - the wording here still does not make sense - "genomic characterization" is not " analysed ".

Authors' response: Thank you for your comment. We have revised the sentence to accurately reflect the performed. The text now reads: We determined antimicrobial susceptibility, conducted genomic characterization, and assessed plasmid conjugation.

48. Line 40 - this should be IncI1alpha, not IncI1Ialpha

Authors' response: We have rephased "IncI1Ialpha" as "IncI1alpha".

49. Line 55 - "is ST29" or "belongs to ST29" here is better than "affiliated with ST29".

Authors' response: We have rephased "affiliated with ST29" as "is ST29" (Lines 55-6).

50. Line 64 - "OXA-type" is incorrect here - at most it should only be "OXA-10-type".

Authors' response: We have rephased "OXA-type" as "OXA-10-type" (Line 65).

51. Lines 78-9 - the wording here needs to be made more accurate and more grammatically correct. It should be "the blaKPC, ... or blaOXA-48-like genes", but "and/or" should be used instead of "or" if any have examples have >1 of these genes, and "like" should not be subscript.

Authors' response: Thank you for your comment. We have revised the sentence to accurately reflect the performed. The text now reads: "the bla_{KPC}, bla_{IMP}, bla_{NDM}, bla_{VIM}, and/or bla_{OXA-48}-like" (Line 79).

52. Line 82 - words missing.

Authors' response: The corrected now reads: the ability of these enzyme's hydroze β-lactams is unaffected by available β-lactamase inhibitors (e.g., avibactam, relebactam, and vaborbactam) (Lines 82-3).

53. Line 111 - lowercase I for imipenem

Authors' response: We have rephased "Imipenem" as "imipenem" (Line 109).

54. Line 126 - this size for pSAL22057-OXA is wrong - it should be "237,376 bp" and this formatting should also be used for the other numbers i.e. there should be a space between numbers and units and for and no spaces around the comma.

Authors' response: The above errors have all been corrected (Lines 128, 146-7, 155, 181).

55. Lines 133-5 - are all resistance genes in the isolates listed here? If so, "included" here is incorrect and must be removed.

Authors' response: The "included" have be removed (Line 140).

56. Line 136 - "the plasmid" is not needed here - the "p" in the name indicates this.

Also check and remove/reword throughout e.g. Lines 160, 161, figure legends.

Authors' response: All mentions of "the plasmid" have been removed. We have also checked the entire manuscript and applied these changes to Lines 160-161 and the figure legends.

57. Lines 125-40 - this could be condensed by stating the plasmid sizes and the resistance genes that they carry.

Authors' response: We have condensed the text as suggested by integrating the plasmid sizes with the resistance genes they carry (Lines 140-8).

58. Lines 152-3 - this an incomplete sentence that does not make sense and does not seem necessary.

Authors' response: We agree that the sentence was redundant and have removed it from the manuscript to improve conciseness.

59. Lines 153, 157, 171 - CP162097 is now listed on both lines 153 and 157 and CP195667 on both Lines 157 and 171. Each only needs to be given once.

Authors' response: We thank the reviewer for pointing this out. The duplicate listings of plasmid accessions CP162097 and CP195667 have been removed, and each is now cited only once in the manuscript.

60. Line 155 - this plasmid does not carry blaOXA-10.

Authors' response: We have removed it from the manuscript.

61. Line 156 - "The carbapenem resistance gene" is not needed here.

Authors' response: We have removed it from the manuscript.

62. Lines 157, 172- what is the relevance of overall GC content? Particularly for the

blaOXA-10 plasmid, that appears to have many different insertions that could have different GC contents.

Authors' response: We agree that the overall GC content has limited interpretive power for a plasmid with a likely mosaic structure, such as pSAL22057-OXA, as it averages the potentially different GC contents of various inserted elements. In fact, the central GC-content ring in Figure S1 and S2 was included to visualize these very differences. The prominent peaks and troughs in that track provide direct evidence of localized regions with distinct sequence compositions, confirming the integration of foreign DNA. In response to this comment, we have toned down the emphasis on the overall plasmid GC content and modified the discussion to state: "The composite structure of pSAL22057-OXA, characterized by its variable GC content, arises from the acquisition of discrete modules via horizontal gene transfer. (lines 302-3)" We believe this significantly strengthens our interpretation.

63. Line 182 - wording does not make sense.

Authors' response: he wording has been revised for clarity and sense. Please see the revised manuscript. (lines 190-1,199-202).

64. Line 187 - the title of Ref 10 says "identified in the US". The isolate was from a patient from India.

Authors' response: Thank you for your insightful comment. Upon careful review of the literature, we confirm that the study reports a *Salmonella* strain carrying bla_{NDM-1} that was first isolated and identified in a hospital in Maryland, USA. While the infection was likely acquired in India, the key point of the report is that the bacterial

isolation, phenotypic characterization, and genetic confirmation (including the detection of *bla*$_{NDM-1}$) were all conducted in the United States. In response to this comment, we have modified the discussion to state: The first example of *bla*$_{NDM-1}$ in *Salmonella* was likely acquired in India but was identified in the United States in 2011 (lines 232-3)

65. Line 191 - "designated as" is not needed here.

Authors' response: We have removed it from the manuscript.

66. Line 208: "describeded the first report" needs fixing.

Authors' response: Thank you for pointing this out. We agree that the phrasing was incorrect and have revised the sentence for clarity and correctness. The text now reads: "In 2019, Han et al. reported the first co-occurrence of *bla*$_{NDM-1}$ and *bla*$_{OXA-10}$ in a *Citrobacter braakii*."(lines 253-4)

67. Line 214- "Escherichia coli should be in italics"

Authors' response: We thank the reviewer for pointing this out. The correction has been made, and *Escherichia coli* is now italicized in the revised manuscript.

68. Line 219 - "it is worth noting that" "are classified", "is carried", as all of these things are still true.

Authors' response: Thank you for this helpful suggestion to improve the clarity and academic tone of the sentence. We have revised it in the manuscript as suggested to remove the redundant phrase and use the present tense for stating current scientific facts. The sentence now reads: The pHNAHS65I-1 and pNDM13-SR33 are classified as IncI1 plasmids originating from China.

69. Lines 222-3 - blaNDM-13 was transferred from SAL22057 to C600, or transferred on pSAL22057-NDM, not from pSAL22057-NDM.

Authors' response: We sincerely thank you for this precise and correct comment. We agree that the original phrasing was logically inaccurate. We have revised the sentence to clarify that the *bla*$_{NDM-13}$ gene was transferred on the plasmid, not from it. The sentence has been amended in the revised manuscript as follows: "The transfer of the *bla*$_{NDM-13}$-harboring plasmid pSAL22057-NDM to *E. coli* C600 was experimentally verified through conjugation assays in this study."(lines 274-5)

70. Lines 224-5 - what is the evidence that suggests this?

Authors' response: Thank you for your valuable question. The evidence supporting the potential dissemination of the *bla*$_{NDM-13}$-carrying plasmid from animal foodstuff to humans comes from Fu et al. (2024, Emerg Microbes Infect). First, *bla*$_{NDM}$ prevalence rose gradually along the animal breeding–slaughtering–retail chain (e.g., 2.0%/4.70% in pig/chicken farms vs. 34.26%/65.56% in retail meat), with 63.11% of school cafeteria pork positive, and *bla*$_{NDM}$ detected in children's hospital samples (21.68% intestinal, 19.80% infection). Second, the IncX3 plasmid (dominant for *bla*$_{NDM}$, 57.7% in *E. coli*, 91.3% in *K. pneumoniae*) was widespread across the chain and humans, with high genomic similarity between animal and human isolates, confirming its role in cross-species transmission. The *bla*$_{NDM-13}$-carrying plasmid identified in our study shares conserved genetic contexts (IS*1294*, *orf*, ΔIS*Aba125*, *bla*$_{NDM-13}$, *ble*$_{MBL}$ and *trpF*) with plasmids from animal-derived *Enterobacteriaceae* reported in public databases. Phenotypic conjugation assays confirmed the plasmid's ability to transfer

between bacterial strains, consistent with the transmissible feature of NDM-harboring plasmids in the food chain.

Collectively, the transmissibility of *bla*$_{NDM-13}$-carrying plasmids, the high prevalence of NDM-positive bacteria in animal food, and the genomic homology between animal-derived and human-derived NDM plasmids provide comprehensive evidence for the proposed dissemination pathway.

References

Fu B, Xu J, Yin D, et al. Transmission of *bla*$_{NDM}$ in Enterobacteriaceae among animals, food and human. Emerg Microbes Infect. 2024;13(1):2337678.

71. Line 261 - "carrying"

Authors' response: Thank you for your careful review and valuable comment on the spelling of "carring". This is a typographical error, and the correct spelling should be "carrying". We have revised the sentence to "Similarly, our analysis of pSAL22057-OXA, carrying *bla*$_{OXA-10}$ revealed that IS*26* elements consistently border this gene" in the manuscript to ensure the accuracy of the text. We apologize for any inconvenience caused by this oversight.

72. Line 270 - suggest rewording "isolate ... was isolated"

Authors' response: We agree with the reviewer and have rephrased the sentence to improve its flow. The revision is as follows: "An isolate designated SAL22057 was obtained from an 18-month-old boy's fecal sample at a children's hospital in June 2022."

73.Lines 314-5 - wording does not make sense.

Authors' response: We have rephrased the sentence to improve clarity, as suggested: "A search for transposon and IS elements was performed using the IS Finder database (https://www-is.biotoul.fr/)."(Lines 385-6)

74. Line 319 - "Overnight cultures", "recipient" not "receptor"

Authors' response: Thank you for this correction. The sentence has been revised as suggested: "Overnight cultures of the recipient *E. coli* C600 …" (Line 390)

75. Line 323 - something has gone wrong here.

Authors' response: Thank you for pointing out this error. The sentence has been corrected to read: "The mixtures were then incubated in parallel at three different temperatures: 25°C, 30°C and 37°C, for 18 hours each, with three independent replicates per condition. Following incubation, transconjugants were selected on..."(Lines 393-6)

76. Line 333 - how were they chosen?

Authors' response: Thank you for raising this important point regarding the selection of reference genomes. The reference sequences were selected based on the following criteria: high BLASTn similarity (≥95% identity, >90% query coverage), epidemiological relevance, and sequence completeness. The final selection was validated through visual alignment with Easyfig. The revised sentence in the manuscript now reads: "Reference sequences for each fragment were selected based on high similarity (≥95% identity, >90% coverage), source relevance, and completeness, with final validation through Easyfig alignment." (Lines 406-7)

77. References: There are still problems with missing details, journal names not

correctly abbreviated etc and some citations seem to have been mixed up (see comments above).

Authors’ response: Thank you for highlighting these remaining issues with the references. We have now carefully checked and revised the entire reference list accordingly. The missing details have been added, all journal names have been corrected to their standard abbreviations, and any mixed-up citations have been identified and properly placed. The reference section has been thoroughly updated to ensure accuracy and consistency.

78. Table 1 - in addition to inconsistencies between MICs in this table and text and the Table 1 in the original manuscript (see comment 1 above), CXT should be CTX (see Line 110), IMP is normally abbreviated IPM (also Line 111) and AZM (usual abbreviation for azithromycin) should presumably be ATM, as on Line 113.

Authors’ response: Thank you for your meticulous attention to detail in reviewing Table 1 and the corresponding text. We sincerely appreciate you identifying these inconsistencies and typographical errors. We have carefully addressed each point as follows. We have conducted a thorough, line-by-line comparison of the MIC values presented in Table 1, the main text, and the original submission's table. All discrepancies have been identified and rectified to ensure the data is now perfectly consistent throughout the entire manuscript. The values in the current version of Table 1 are the correct and final ones. We have corrected the antibiotic abbreviations to align with standard microbiological and CLSI conventions: CXT has been corrected to CTX (cefotaxime) in both Table 1 and on Line 110 of the text. IMP has been

corrected to IPM (imipenem) in both Table 1 and on Line 111 of the text. AZM (azithromycin) in Table 1 was indeed an error; it has been corrected to ATM (aztreonam), consistent with the listing on Line 113.

We have also taken this opportunity to perform a final check of all other antibiotic abbreviations in the manuscript to ensure uniformity. We are confident that these revisions have resolved the issues and greatly improved the accuracy and professionalism of our data presentation.

Reviewer #2 (Comments for the Author):

All my concerns have been addressed. No further comments.

Reviewer #4 (Comments for the Author):

The case (pediatric S. enterica serovar Stanley, ST29, co-harboring blaNDM-13 and blaOXA-10 on distinct plasmids) is noteworthy, but claims and evidence need tightening.

Major comments

1.2 Conjugation experiments are incomplete. Only blaNDM-13 transfer was shown. Test/ report transfer of the IncHI2/HI2A OXA-10 plasmid (optimize temperature, duration), and quantify frequencies; confirm structures in transconjugants.

Authors' response: Thank you for this insightful comment, which has helped us significantly strengthen the experimental evidence in our study. We have significantly expanded and optimized the conjugation experiments in the revised manuscript (Lines

390-402). To specifically address the transfer of the IncHI2/HI2A plasmid carrying *bla*$_{OXA-10}$, we optimized the assay conditions based on the known temperature sensitivity of HI2-type plasmids. The conjugation was performed in parallel at three different temperatures: 25°C, 30°C, and 37°C, with three independent replicates per condition. Transconjugants were selected on media containing rifampicin and CTX. Subsequently, three transconjugant colonies from these experiments were subjected to PCR for *bla*$_{NDM-13}$ and *bla*$_{OXA-10}$, antibiotic susceptibility testing, and replicon typing for IncI1 and IncHI2 plasmids.

1.3 IS-mediated mechanism claims are over-stated. Map IS boundaries (IR/DR), show long-read evidence spanning events, and present integron/cassette architecture; downgrade language to "likely/consistent with."

Authors' response: Thank you for highlighting this weakness in our original submission. We have undertaken the following major revisions to rectify this issue: We have generated a new, detailed Figure 1,2 that provides a comprehensive schematic of the genetic context. These figures now clearly map the boundaries of the relevant Insertion Sequences (ISs), explicitly annotating the Inverted Repeats (IRs) generated by the transposition event at the target site. Furthermore, the figure illustrates the complete architecture of the integron and the associated gene cassette array harboring the *bla*$_{NDM-13}$ gene, showing its precise location relative to the flanking IS elements. To provide direct visual validation of the proposed genetic rearrangement, we have integrated a new panel featuring long-read (Nanopore) sequencing data. The alignment of continuous long reads that span the entire locus

definitively confirms the physical linkage between the IS elements, the integron, and the *bla*$_{NDM-13}$ gene on a single plasmid molecule. This evidence circumvents the inherent limitations of short-read assemblies, unequivocally ruling out misassembly as a possible explanation for the observed genetic architecture. In accordance with your suggestion, we have systematically revised the text to replace definitive claims with more cautious and accurate language. For instance: 1. "Therefore, we propose that IS*1294* may play a role in the mobilization and dissemination of *bla*$_{NDM-13}$." has been changed to "Therefore, the genomic data support the possibility that IS*1294* contributes to the mobilization and dissemination of *bla*$_{NDM-13}$." (Lines 301-2). 2. "This highlights IS26's broad and significant role as a genomic architect of antimicrobial resistance within *Enterobacteriaceae*" has been changed to "This conserved genomic architecture, with resistance clusters invariably bordered by IS26, is consistent with IS26-mediated dissemination being a primary driver of resistance spread." (Lines 312-4). Similar changes have been made throughout the Abstract sections (e.g., Lines 43-6)

3. Phylogenetic/epidemiologic context is thin. Add cgMLST/ core-genome tree and geographic/host distribution of related ST29 and homologous IncI1-NDM-13 / IncHI2-OXA-10 plasmids to support public-health relevance.

Authors' response: Thank you for this valuable and constructive suggestion. We agree that expanding the phylogenetic and epidemiologic context is crucial for underscoring the public health relevance of our findings. Additional analyses were performed and are included in Section 5.7 of the revised manuscript (Lines 412-22): Based on the

complete sequences of pSAL22057-NDM and pSAL22057-OXA, homologous sequences were identified via BLASTn searches against the NCBI non-redundant nucleotide database. Whole-genome sequences of source strains carrying these homologous plasmids were subsequently downloaded from PCBI-Pathogen, along with genome sequences of salmonella ST29 obtained in PATRICBI database. Together with SAL22057, these sequences were analyzed using kSNP3 ((https://doi.org/10.1093/bioinformatics/btv271)). A phylogenetic tree was constructed using PhyML based on concatenated, qualified core genome SNPs, with the whole genome SAL22057 as the reference. The tree was visualized and annotated with epidemiological metadata (sequence type, serovar, geographic origin, host, year of isolation, and plasmid replicon type) using the iTOL (https://itol.embl.de).

We have incorporated these results—specifically, the new phylogenetic tree, distribution table, and corresponding interpretation—into the revised manuscript (Lines 207-30). The discussion section has been expanded accordingly to address their significance (Lines 241-3).

4. Genotype-phenotype alignment needs clarity. Explain aztreonam susceptibility under the recorded β-lactamase background; if possible, add AZT-AVI or CAZ-AVI{plus minus}AZT synergy data to enhance translational value.

Authors' response: Thank you for your valuable comment regarding genotype-phenotype alignment and aztreonam (ATM) susceptibility, which helps strengthen the translational relevance of our study.

4.1 Correction of Typographical Error

We have re-verified the aztreonam susceptibility data, and the correct result is an MIC value of ≤1 μg/mL, confirming the isolate is susceptible to ATM. A typographical error in the original manuscript incorrectly denoted "ATM" as "AZM" (azithromycin), and this mistake has been rectified in the revised version.

4.2 Genotype-Phenotype Alignment for ATM Susceptibility

Consistent with the findings of Shrestha et al. (2015), our isolate harbors an NDM-type metallo-*β*-lactamase (MBL) variant. NDM-type MBLs, including the novel NDM-13 identified in their study, are well-documented to not hydrolyze aztreonam (Shrestha et al., 2015; Antimicrob Agents Chemother., Table 2). This enzymatic characteristic directly explains the phenotypic susceptibility of our isolate to ATM, establishing clear alignment between the β-lactamase genotype and ATM susceptibility phenotype.

4.3 Susceptibility to AZT-AVI and CAZ-AVI

Regarding the reviewer's suggestion to include data on ATM-AVI (avibactam) or CAZ-AVI (ceftazidime-avibactam) ± ATM synergy, we agree on its general importance for treating MBL-producing *Enterobacterales*. However, in the specific context of our isolate, which is already susceptible to aztreonam alone, the addition of avibactam is mechanistically expected to confirm, but not significantly enhance, this susceptibility. The primary role of avibactam in the ATM-AVI combination is to protect aztreonam from serine β-lactamases. Since aztreonam is effective on its own in our strain, it demonstrates that there is no significant serine β-lactamase-mediated resistance to overcome. Therefore, we would anticipate susceptibility to ATM-AVI,

but its activity would be congruent with the aztreonam monotherapy result.

We appreciate your meticulous review, which has helped clarify the genotype-phenotype relationship. The corrected data and supplementary rationale have been integrated into the revised manuscript, and the reference to Shrestha et al. (2015) has been added to support the arguments. In the revised manuscript, we added supplementary rationale as follows: NDM-type metallo-β-lactamases (e.g., NDM-13) cannot hydrolyze aztreonam (ATM), which supports the consistency between our isolate's ATM-susceptible phenotype and its NDM-harboring genotype (264-6).

Minor comments

1. Systematic copy-editing (spelling, grammar, units, spacing, "33rd ed." for CLSI, consistent tense and abbreviations).

Authors' response: Thank you for your careful review. The suggested changes have been systematically implemented throughout the manuscript. This includes: Correcting the CLSI reference to "33rd ed." Ensuring consistency in verb tense, abbreviations, and terminology. Verifying the correct use of units, spelling, and spacing

2. Clarify MIC methodology, replicates, media, and incubation details; clean up typos in protocols.

Authors' response: Thank you for your comment. We appreciate the opportunity to clarify and improve the methodology section. We have revised the text accordingly to provide a more comprehensive description of the antimicrobial susceptibility testing and carbapenemase detection protocols (Line 338-51). The updated text now clearly

specifies the following:

Methodology: The MICs were determined using the broth microdilution method.

Replicates: All MIC tests were performed in triplicate to ensure reproducibility.

Cleaning up protocols: The reference citation format has been standardized to the journal's requirements. The description of the carbapenemase detection test has been expanded to include critical details such as disk concentrations, disk placement distance, and the interpretation criterion.

3. Improve readability (directionality, boundary markers, scale bars, clear color keys); correct phrasing ("closely related," not "closed").

Authors' response: Thank you for your comment. We have implemented the following changes to enhance our figures and correct textual error. The text misalignment in Figure B was a typesetting error during the figure preparation. We have now fixed this issue and carefully checked all other figures to ensure proper formatting. Additionally, the label for direct repeats (DRs) has been removed from Figure 1,2, as our re-analysis verified their absence in the specified region of pSAL22057-NDM and pSAL22057-OXA. We have revised the color keys and legends in Figure1,2 to ensure they are intuitive and unambiguous. We believe that these collective edits have substantially improved readability, accuracy, and scientific rigor of the figure.

4. Add columns for replicate counts and fold-change before/after transfer; ensure totals and spacing are correct.

Authors' response: Thank you for your valuable comment. We have thoroughly revised both the manuscript text (Line 118-25) and Table 1 to fully address your

comment on adding replicate counts, fold-change values, and ensuring correct formatting. We have added three dedicated columns in Table 1 under the "Conjugation" section:

"Replicate Counts (n)": This column explicitly states the number of independent biological replicates (n=3) for each transconjugant group. "Conjugation Frequency": This column now presents the quantitative transfer frequencies for the $bla_{\text{NDM-13}}$-harboring plasmid at 25 °C, 30 °C, and 37 °C. "Fold Change (vs. 37°C)": This column provides the calculated increase in conjugation frequency at 25 °C and 30 °C relative to the reference frequency at 37 °C (2.7-fold and 4.4-fold, respectively). The added quantitative data from the table are now reflected and described in the revised Results section. Specifically, we state the conjugation frequencies and their fold-increase, and explicitly mention that the MICs for MEM and IPM in transconjugants were at least 64-fold higher than those of the recipient strain, providing the "fold-change before/after transfer" for the phenotypic data as implied by your comment. Thank you for prompting these improvements.

Table 1 Minimal inhibitory concentrations (MICs, μg/ml) of antimicrobial agents against SAL22057, transconjugants and recipient strain

	S. Stanley *SAL*22057 (donor strain)	SAL22057-*E. coli* C600 (transconjugants, 37°C)	SAL22057-*E. coli* C600 (transconjugants, 30°C)	SAL22057-*E. coli* C600 (transconjugants, 25°C)	*E. coli C*600 (recipient strain)
β-lactamase genes	$bla_{\text{NDM-13}}$, ble_{TEM}, $bla_{\text{OXA-10}}$	$bla_{\text{NDM-13}}$	$bla_{\text{NDM-13}}$	$bla_{\text{NDM-13}}$	-
Antimicrobials					
PIP	>128	>128	>128	>128	≤4
TZP	>128/4	>128/4	>128/4	>128/4	<=4/4
AMC	>32/16	>32/16	>32/16	>32/16	4/2
CAZ	>64	>64	>64	>64	0.5
CTX	>64	>64	>64	>64	≤1
FEP	>32	16	32	32	≤0.12
ATM	≤1	≤1	≤1	1	≤1
IPM	16	16	16	16	≤0.25
MEM	32	16	16	16	≤0.25
LEV	1	1	1	1	0.5
CIP	0.5	≤0.25	≤0.5	≤0.5	≤0.25
SXT	>16/304	≤1/19	≤0.25	≤0.25	≤1/19
POLB	0.25	0.25	0.25	0.25	0.25
TGC	0.125	0.125	0.25	0.25	0.125
Conjugation					
Replicate Counts (n)	NA	3	3	3	NA
Conjugation Frequency (mean)	NA	~5.2×10^{-5}	~1.4×10^{-4}	~2.3×10^{-4}	NA
Conjugation frequency Fold Change (vs. 37°C)	NA	NA	~2.7	~4.4	NA

-, No data
NA, not available

Re: Spectrum03207-24R2 (**Carbapenem Resistance Mediated by *bla*$_{NDM-13}$ and Co-occurrence of *bla*$_{OXA-10}$ in a *Salmonella* Stanley ST29 strain in China**)

Dear Mrs. Juanjuan Zhou:

Thank you for the privilege of reviewing your work. Below you will find my comments, instructions from the Spectrum editorial office, and the reviewer comments.

Please return the manuscript within 60 days; if you cannot complete the modification within this time period, please contact me. If you do not wish to modify the manuscript and prefer to submit it to another journal, notify me immediately so that the manuscript may be formally withdrawn from consideration by Spectrum.

Revision Guidelines
To submit your modified manuscript, log into the submission site at https://spectrum.msubmit.net/cgi-bin/main.plex. Go to Author Tasks and click the appropriate manuscript title to begin. The information you entered when you first submitted the paper will be displayed; update this as necessary. Note the following requirements:

• Upload point-by-point responses to the issues raised by the reviewers in a file named "Response to Reviewers," NOT in your cover letter.
• Upload a compare copy of the manuscript (without figures) as a "Marked-Up Manuscript" file.
• Upload a clean .DOC/.DOCX version of the revised manuscript and remove the previous version.
• Each figure must be uploaded as a separate, editable, high-resolution file (TIFF or EPS preferred), and any multipanel figures must be assembled into one file.
• Any supplemental material intended for posting by ASM should be uploaded with their legends separate from the main manuscript. You can combine all supplemental material into one file (preferred) or split it into a maximum of 10 files with all associated legends included.

For complete guidelines on revision requirements, see our Submission and Review Process webpage. Submission of a paper that does not conform to guidelines may delay acceptance of your manuscript.

Data availability: ASM policy requires that data be available to the public upon online posting of the article, so please verify all links to sequence records, if present, and make sure that each number retrieves the full record of the data. If a new accession number is not linked or a link is broken, provide Spectrum production staff with the correct URL for the record. If the accession numbers for new data are not publicly accessible before the expected online posting of the article, publication may be delayed; please contact production staff (Spectrum@asmusa.org) immediately with the expected release date.

Publication Fees: For information on publication fees and which article types are subject to charges, visit our website. If your manuscript is accepted for publication and any fees apply, you will be contacted separately about payment during the production process; please follow the instructions in that e-mail. Arrangements for payment must be made before your article is published.

ASM Membership: Corresponding authors may join or renew ASM membership to obtain discounts on publication fees. Need to upgrade your membership level? Please contact Customer Service at Service@asmusa.org.

The ASM Journals program strives for constant improvement in our submission and publication process. Please tell us how we can improve your experience by taking this quick Author Survey.

Thank you for submitting your paper to Spectrum.

Sincerely,
Jian Peng
Editor
Microbiology Spectrum

Reviewer #1 (Comments for the Author):

This revised manuscript describes a Salmonella Stanley isolate that has an IncI1 plasmid with blaNDM-13 and an HI2 plasmid that carries many antibiotic resistance genes (ARG), including blaOXA-10. The original version (Spectrum0307-24) stated that these two bla genes were on the same plasmid, but the revised 03207R1 reported that they are actually on separate plasmids. In the response for R2, the authors now agree that blaOXA-10 is not normally considered a carbapenemase in Enterobacterales

such as S. Stanley and now state: "the primary focus of our manuscript is the characterization of NDM-13 as the key carbapenemase in Salmonella strain SAL22057". However, blaOXA-10 is still singled out from other ARG on the HI2 plasmid for special mention, and inappropriate descriptions relating to this gene are still used in places. There are also still other problems, including with the selection used in new conjugation experiments for the HI2 plasmid, with descriptions around IS1294 and many other points.

MAJOR POINTS
1) blaOXA-10
PREVIOUS COMMENT: I still believe that the focus on OXA-10 as a carbapenemase and apparently suggesting that it is equivalent to NDM and other "classic" carbapenemases is not appropriate.
RESPONSE: All references to OXA-10 in the manuscript have been revised to align with the reviewer's accurate assessment. We now state that OXA-10 is primarily a narrow-spectrum β-lactamase and, crucially, that it is not universally classified as a carbapenemase in the same category as NDM, KPC, or OXA-48-like enzymes, especially in Enterobacterales like Salmonella. We acknowledge that its reported carbapenemase activity is weak, host-dependent (as in A. baumannii), and not representative of most alleles. We have strengthened the statement that NDM-13 is the unequivocal and primary determinant of the carbapenem-resistant phenotype in SAL22057, as correctly noted by the reviewer. Instead of framing OXA-10 as a carbapenemase, we now present its presence as part of the broader multidrug-resistant (MDR) arsenal of this isolate (Lines 87-90). We have de-emphasized OXA-10's role regarding carbapenems and reframed its significance to be accurate and consistent with the literature and the reviewer's expert opinion.
NEW COMMENT: If this is now acknowledged, why is there still specific mention of blaOXA-1 above all of the other ARG?

PREVIOUS COMMENT: Title - should be updated after changes have been made.
RESPONSE: The updated title is: "Carbapenem Resistance Mediated by blaNDM-13 and Co-occurrence of blaOXA-10 in a Salmonella Stanley ST29 Strain in China". This modification explicitly states that NDM-13 is the primary mediator of the carbapenem-resistant phenotype, while the presence of blaOXA-10 is reported as a significant co-occurring genetic feature. We have also ensured the manuscript text consistently supports this clarification, particularly in the abstract (Lines 33-4) and discussion (Lines 257-262) sections. We believe this revised title addresses your concern by eliminating potential misunderstanding and more precisely representing our results
NEW COMMENT: Given that the authors now agree that there is no evidence that OXA-10 contributes to carbapenem resistance and their statement that the focus of the manuscript is on blaNDM-13, it's not clear why blaOXA-10 is still mentioned in the title. What makes blaOXA-10 more of a "significant co-occurring genetic feature" than the other ARG on the HI2 plasmid? The title could instead reflect that this is a highly resistant isolate.
Also, there are still problems in places around descriptions of blaOXA-10:
Line 33 (Abstract) - why is the co-occurrence relevant?
Line 40 (Abstract) - why single out blaOXA-10 here? This could state that pSAL22057-OXA has # ARG (and say "including blaOXA-10, if you want to justify why it's called pSAL22057-OXA).
Line 44 (Abstract) - again , why specifically mention blaOXA-10 here?
Line 54-5 (Importance) - blaOXA-10 is only one of several other ARG.
Line 59 (Importance) still says "two high-risk carbapenemase genes, blaNDM-13 and blaOXA-10".
Line 65 (Importance) still uses "OXA-10 type carbapenemases"
Lines 323-4 (Conclusions) still state "The presence of these critical resistance genes in a virulent, multidrug-resistant S. Stanley ST29" after discussing blaNDM-13 and blaOXA-10.

PREVIOUS COMMENT Line 54 - I don't think that it can be argued that blaOXA-10 is "critical" in SAL22057.
RESPONSE: The text now reads: "This study identified a carbapenem-resistant Salmonella Stanley strain, SAL22057, from a pediatric patient, which co-harbored the carbapenemase gene blaNDM-13 and blaOXA-10 β-lactamase gene."
NEW COMMENT: Again, I don't see why blaOXA-10 is still singled out for special attention.

2) New conjugation experiment
PREVIOUS COMMENT: It might also be good to look for conjugation of pSAL22057-NDM but different selection conditions would be needed (not necessarily for blaOXA-10, as one of the other resistance genes on the plasmid could be selected for) and noting that HI2 plasmid transfer better at temperatures around 25-30 degrees, rather than 37.
AUTHORS' RESPONSE: We have performed an additional, optimized conjugation experiment as follows: Temperature Adjustment: Based on the well-documented temperature sensitivity of many conjugative systems (including HI2-type plasmids), we repeated the conjugation assay at 25{degree sign}C, 30{degree sign}C and 37{degree sign}C, for 18 hours each, with three independent replicates per condition. To specifically detect the transfer of the plasmid carrying blaOXA-10 (pSAL22057-OXA), we employed a targeted selection strategy. Transconjugants were selected on Mueller-Hinton agar supplemented with rifampicin (200 mg/L, to counter select the donor) and CTX (2 mg/L, to select for the OXA-10 β-lactamase). The Methods 5.5 section (Lines 393-395) now described the optimized conjugation protocol. The relevant Results (Lines 116-26) and Discussion (Lines 274-78) section has been updated accordingly.
NEW COMMENT: I think there is a problem here, as blaOXA-10 does not encode an ESBL (and neither does the only other bla gene on pSAL22057-NDM, blaTEM-1) using CTX would likely fail to select transconjugants that carry it. As mentioned in the comment above it should be possible to select for one of the other ARG on pSAL22057-OXA.

3) Lines 125-40 -
PREVIOUS COMMENT: this could be condensed by stating the plasmid sizes and the resistance genes that they carry.
RESPONSE: We have condensed the text as suggested by integrating the plasmid sizes with the resistance genes they carry (Lines 140-8).
PREVIOUS COMMENT: - there should not be a space after aac or aph in these gene names.
RESPONSE: We have removed the space after aac or aph in these gene names.
NEW COMMENT: this can still be condensed further by avoiding repetition and combining with Lines 153-4, e.g. "...ARG were identified: an aac(6′)-Iaa gene (intrinsic in Salmonella) on the chromosome, blaNDM-13 and blaMBL on pSAL22057-NDM (IncI1 87,928 bp) and 15 other ARG on pSAL22057-OXA (IncHI2, 237376 bp). The remaining plasmids".
Lines 149-50 - this is not a complete sentence and is unnecessary. Lines 150-2 are also unnecessary.
Lines 140-2 - the spaces in aac and aph gene names have not been removed. Also "(A)" of tet(A)" should not be in italics.

4) pSAL22057-NDM, IS1294
PREVIOUS COMMENT: IS1294 is a member of the IS91 family, rolling circle elements that are known to be able to move adjacent regions (see PMID: 10873528). It should be possible to tell fairly confidently if IS1294 was responsible for inserting part of Tn125 into the pSAL22057-NDM, so this needs to be looked at properly. On a quick look, it seems like MN219406 and CP092912 have a clear insertion vs. CP037994, while pSAL22057-NDM has a deletion in this region.
RESPONSE: Comparative visualization of the insertion of IS1294 upstream of ISAba125 resulted in the truncation of the latter, retaining only a 44-bp fragment (positions 4612-4655) corresponding to the 3'-end of the tnpA gene. The terIS region of IS1294 (4586-4611 bp) is located directly adjacent to the truncated ΔISAba125. Upon integration upstream of ISAba125, IS1294 serves as a key element driving the mobilization of the "IS1294-blaNDM-13 unit" The plasmid pgs_wt5 appears to represent the ancestral state of such plasmids before the acquisition of this unit. In both pNDM13-SR33 and pHNAHS65I-1, the "IS1294-blaNDM-13 unit" is inserted into a region encoding a hypothetical protein located upstream of the proQ gene. This insertion splits the hypothetical protein into two segments, which we refer to here as hp-left and hp-right for clarity. The hp-left segment is 241 bp in length. Sequence analysis reveals that in both pNDM13-SR33 and pHNAHS65I-1, this segment (positions 4713-4953) is directly adjacent to a terIS region (4,954-5,053 bp), with no nucleotides in between. The hp-right segment is 819 bp long and shares a 25-bp repeat with the 3′ end of the trpF gene. Based on this, we speculate that the integration of the "IS1294-blaNDM-13 unit" may have involved homologous recombination with this hypothetical protein. These findings support the notion that the "IS1294-blaNDM-13 unit" exhibits a specific insertion preference in such plasmids, and that the 25-bp homologous sequence likely plays an important role in determining its integration site.
PREVIOUS COMMENT: Line 233 - it's more than IRR - it's 51 bp of the IRR end. Does this segment include the promoter?
RESPONSE: You are correct in pointing out that the region in question comprises not only the IRR sequence but also an adjacent 51 bp segment from the end of the IRR, which indeed includes part of the promoter region. We acknowledge the inaccuracy in our initial description regarding the IRR boundary. We have now corrected this inaccuracy in the revised version of the manuscript, clarifying the precise sequence composition in this region. Furthermore, we have incorporated additional data from the newly added sample psg_wt5, which has led to a refined interpretation of the results (Lines 161-163). This includes a more detailed analysis of the oriIS/terIS region flanking IS1294 and the truncated state of ISAba125 in this context. These updates and a more comprehensive discussion of the findings are provided in our response to your Question #13 within the point-by-point reply.
NEW COMMENTS (Lines 160-178). This section still has problems and is unnecessarily complex and can be simplified to the main points, with details in the figure.
Line 162 - what is "orf"? Is it inside IS1294?
Line 163 - IS1294 is inserted in ISAba125, not upstream of it - deltaIS1294 is used on Line 162 and "truncation" of ISAba125 is mentioned in the response and text, which agree with this.
Line 164 - this should state that these are the positions in NZ_CP195667 and be for the 51 bp region.
Line 164-5 - this still says "a 44 bp fragment corresponding to the 3′ end of the tnpA gene" despite the authors agreeing in their response that the ISAba125 segment is 51 bp (which includes the IR). The wording must reflect this i.e. 51 bp of the IRR (or right end) not the length of the tnpA gene (which is irrelevant) remains and state that the promoter is present, as this is important.
Lines 165-6 - try and incorporate this information when discussing the truncation to avoid repetition.
Line 167 etc - I don't think that "IS1294-blaNDM-13 unit" needs quotes and it's really IS1294-deltaISAba125-blaNDM-13. Maybe also give the (correct) length of this insert - see comments below.
Lines 167-73 - this can be simplified here and the details given in the legend of Fig. 1. Just say that IS1294-deltaISAba125-blaNDM-13 is inseerted in a gene encoding a hypothetical protein.
Line 171 - positions in what? As the two other plasmids are now being discussed, this is not clear.
Line 173 - this doesn't make sense to me. What are the positions corresponding to these 25 bp? Comparison of the IS1294-deltaISAba125-blaNDM-13 region in pNDM13SR-33 with Tn125 (HQ857107) gives a matching region of 1963 bp, none which appears to be present in psg_wt5 that lacks the insertion. I would say that hp-right is 812 bp. This needs to be looked at again and corrected as necessary.
Lines 174 - this can be simplified - "reported in this study is that" can be deleted and just state that pSAL22057-NDM has an X bp deletion compared with the other two plasmids, putting the detail in the legend of Fig. 1. Fig. 1 also shows a small difference in bleMBL (a 102 by deletion vs. Tn125 and pNDM13SR-33) in pSAL22057-NDM and pHNAHS65I-1, which should be noted in the legend.

5) Shufflon

PREVIOUS COMMENT : Lines 112-3 - IncI plasmids have a shufflon that may not be correctly assembled, even from long-read data (see PMID: 28424528). This needs to be looked at and may explain some differences from other IncI1 plasmids.
RESPONSE: To specifically address your concern, we performed the following additional analysis on the pSAL22057-NDM (IncI1α) plasmid: We manually inspected the assembly graph and raw long-read alignments across the putative shufflon region. We confirmed that the assembly in this region is supported by continuous, high-quality long reads with consistent alignment and no breakpoints indicative of a collapse or misassembly. While the repetitive and recombinogenic nature of shufflon loci is well-documented and can pose challenges, our analysis based on the current data suggests a contiguous and consistent assembly for the specific allele present in our strain. We have now added a brief statement in the revised Results (Section 2.2) and Methods (Section 5.4) to acknowledge this known complexity and to state that our manual validation did not detect evidence of misassembly in this region. The assembly of this plasmid, including its shufflon configuration, is therefore presented as our best representation based on the available sequencing data.
NEW COMMENT: Please add a statement that only a single shufflon arrangement was also confirmed, if this is the case. If not, this should be looked at.

PREVIOUS COMMENT: Line 162 - "pgs_wt5" does not appear to be a complete plasmid.
RESPONSE: We confirm that 'pgs_wt5' corresponds to the complete plasmid pSG_wt5, as designated in the official GenBank record (accession NZ_CP037994). This sequence was resolved using PacBio RSII long-read technology, which robustly generates closed, circular plasmid genomes.
NEW COMMENT: NZ_CP037994 states that it is a linear sequence and the sequence breaks at the shufflon. Shufflons can cause problems even with PacBio data. This needs to be looked at and some justification provided if it is to be described as a complete plasmid.

6) pSAL22057-OXA with blaOXA-10 and other ARG.
PREVIOUS COMMENTS: A much clearer/more accurate description of the resistance regions of pSAL22057-OXA is still needed e.g., Line 176 still refers to "the region", which does not make sense, and "integron 3" (Line 181) is unclear without further explanation. The AMR genes currently listed on Lines 133-5 could be stated here instead, indicating which are in the same cassette array and which others are nearby. Could any IS/Tn explain insertions, are there any DR etc. Again, are there any papers associated with the related plasmids listed in this section that might already have information on this that could be consulted and cited. Information on Lines 245-8 need to be in Results not Discussion.
RESPONSE: The description of the pSAL22057-OXA resistance regions has been amended for greater accuracy and clarity (Lines 195-202). The revisions include a precise explanation of "Integron 3" and a delineation of the resistance gene distribution, specifying their arrangement either within the integron's cassette array or in flanking sequences (Lines 198-200). Furthermore, it is confirmed that no direct repeats (DRs) are associated with this structure.
NEW COMMENTS:
Lines 180-1 - see previous comments and response on average %GC and consider deleting.
Line 190 - correct "cotains".
Line 191 - remove reference to colors here - they are only relevant in the figure legend.
Line 192 - this detail is not needed in the text - just show all IS on Fig. 2.
Line 196 - the name is "lnuF" (starts with lowercase L) not InuF and "(F)" should not be in italics.
Lines 197-7 - spaces in aac and aph gene names need to be removed.
Line 198 - "intergenic" not needed.
Line 199-201 - what is meant by a "core cassette array? "core" can be deleted "(A)" of tet(A) should not be in italics and tnpA of what - this must be stated. Should be "which contains blaOXA-10, is flanked". "b" of blaOXA-1 seems to be underlined and "gene" can be deleted.
Line 202 - there is an extra "t" that needs to be deleted.
Line 203 - "transposition unit' is normally used for segments moved by a single copy of ISEcp1 and related elements. What is meant here?blaNDM-5 shouldnot be described as being in a cassette due to possible confusion with gene cassettes.

7) Discussion and Conclusion
PREVIOUS COMMENT: Parts of the Discussion still repeat Results and it would need to be rewritten after changes to the rest of the manuscript.
RESPONSE: We agree that an effective Discussion should interpret and contextualize the findings rather than reiterate the Results. In revising the manuscript based on all previous comments, we have thoroughly rewritten the Discussion section (Lines 232-7, 241-3, 253-9, 264-6, 275-81，284-317) to address this specific point.
NEW COMMENT: There is still repetition of Results in Discussion and problems in this section and Conclusions also repeat Discussion.
Lines 282-300 (IS1294) - much of this repeats Results and parts are incorrect - see comments above relating to IS1294 and the 25 bp region.
Lines 301-12 - this also still repeats Results. Horizontal gene transfer is between cells. Is there real evidence about acquisition of these modules?
Minor points
Line 233- " context" is not needed here.
Lines 237, 241, 252-61 - again, if the focus is now blaNDM-13, why single out blaOXA-10 for special mention?
Lines 262- might be better at the start of the Discussion.

Line 266 - not clear what "its" is here Specify blaNDM-13.
Lines 268-272 - "The" is not needed before plasmid names or "transfer". "the complete plasmid sequences of" is not needed.
Line 271 - plasmids
Lines 278-80 - odd "1" at start of Line 278, "represent", not represents.
Line 301 - "characterized" is the wrong word here.
Line 308 - "in is" needs fixing.
Line 315, 320-1 - again, why mention blaOXA-10?
Lines 316-7 don't really say anything meaningful.
Line 321 - "This implicating" doesn't make sense, IS26 directly mediates only intracellular transfer, not horizontal gene transfer, and this role was well known before this study.

8) MINOR POINTS RELATING TO PREVIOUS COMMENTS
PREVIOUS COMMENT: Line 40 - this should be IncI1alpha, not IncI1Ialpha
RESPONSE: We have rephased "IncI1Ialpha" as "IncI1alpha".
NEW COMMENT: This is still wrong on Line 60.

PREVIOUS COMMENT: Lines 78-9 - the wording here needs to be made more accurate and more grammatically correct. It should be "the blaKPC, ... or blaOXA-48-like genes", but "and/or" should be used instead of "or" if any have examples have >1 of these genes, and "like" should not be subscript.
RESPONSE: We have revised the sentence to accurately reflect the performed. The text now reads: "the blaKPC, blaIMP, blaNDM, blaVIM, and/or blaOXA-48-like" (Line 79).
NEW COMMENT: This should be "blaOXA-48-like genes", as noted in the previous comment. "blaOXA-48-like without "genes" after it is not good English.

PREVIOUS COMMENT: Line 82 - words missing.
RESPONSE: The corrected now reads: the ability of these enzyme's hydroze β-lactams is unaffected by available β-lactamase inhibitors (e.g., avibactam, relebactam, and vaborbactam) (Lines 82-3).
NEW COMMENT: "enzyme's" should be "enzymes" and "to" is needed before hydolyze and there is no space between "lactams" and "is" in the manuscript.

PREVIOUS COMMENT: Line 136 - "the plasmid" is not needed here - the "p" in the name indicates this. Also check and remove/reword throughout e.g. Lines 160, 161, figure legends.
RESPONSE: All mentions of "the plasmid" have been removed. We have also checked the entire manuscript and applied these changes to Lines 160-161 and the figure legends.
Lines 174, 285-6 - still say "The plasmid p..." - please check again.

PREVIOUS COMMENT: Line 219 - "it is worth noting that" "are classified", "is carried", as all of these things are still true.
RESPONSE: We have revised it in the manuscript as suggested ... and use the present tense for stating current scientific facts. The sentence now reads: The pHNAHS65I-1 and pNDM13-SR33 are classified as IncI1 plasmids originating from China.
NEW COMMENT: There are still problems throughout with tenses.

PREVIOUS COMMENT: Lines 314-5 - wording does not make sense.
RESPONSE: We have rephrased the sentence to improve clarity, as suggested:
"A search for transposon and IS elements was performed using the IS Finder database (https://www-is.biotoul.fr/)."(Lines 385-6)
NEW COMMENT: Change to "transposons and IS" (but note that the ISFinder database only includes a few selected transposons).

PREVIOUS COMMENT: Lines 330-331 - in addition to plasmids, the term "mobile genetic elements" encompasses IS and transposons, which would not be detected by oriTfinder, so is incorrect here.
RESPONSE: We have rephased as "The conjugation probability of pSAL22057-NDM and pSAL22057-OXA were identified by the oriTfinder (https://bioinfo-mml. sjtu.edu.cn/oriTfinder/) (402-4).
NEW COMMENT: "conjugation probability" is still not ideal. Maybe state what can be detected by oriFinder here?

PREVIOUS COMMENT (bla gene PCR Lines 351-2): The text needs to state which genes would be detected by the primers used.
RESPONSE: The carbapenemase genes (blaNDM, blaKPC, blaVIM-1, blaIMP-4, and blaOXA-48-like) were detected by the primers used. We have stated this in revised manuscript (352-3).
NEW COMMENT: should this actually be "blaIMP-4-like" and "blaVIM-1-like" genes? i.e. minor variants in these subfamilies would also be detected? Note that "-like" should not be subscript and needs to be followed by "genes" - see comment above.

PREVIOUS COMMENT: The versions of the plasmid sequences currently in GenBank are still not annotated. Maybe they are waiting to be updated? If not, they should be annotated.
RESPONSE: The plasmid sequences have been annotated. Please refer to the following link:
1.https://www.ncbi.nlm.nih.gov/nuccore/NZ_CP162099.2/

2.https://www.ncbi.nlm.nih.gov/nuccore/NZ_CP162098.2/
3.https://www.ncbi.nlm.nih.gov/nuccore/NZ_CP195668.1/
4.https://www.ncbi.nlm.nih.gov/nuccore/NZ_CP195669.1/
5.https://www.ncbi.nlm.nih.gov/nuccore/NZ_CP195667.1/
6.https://www.ncbi.nlm.nih.gov/nuccore/NZ_CP162097.2/
NEW COMMENT: Lines 436-441 - it seems that e.g. CP162097 is not annotated but NZ_CP162097 is, so maybe the NZ_ accessions should be listed here?

PREVIOUS COMMENT: References: there are still problems with missing details, journal names not correctly abbreviated etc and some citations seem to have been mixed up (see comments above).
RESPONSE: We have now carefully checked and revised the entire reference list accordingly. The missing details have been added, all journal names have been corrected to their standard abbreviations, and any mixed-up citations have been identified and properly placed. The reference section has been thoroughly updated to ensure accuracy and consistency.
NEW COMMENTS:
Ref 3 - should be "lactamase" with lower case "l".
Ref 4 - should be "producing with lowercase "p".
Refs 7, 14 - change format of title from Title Case to Sentence case
Ref 8 - blaNDM-13 is not correctly formatted.

PREVIOUS COMMENT - there are inconsistencies in the MICs of IPM and MEM between Table 1 in the original manuscript (SAL22057, IPM 16, MEM 32; transconjugant, IPM 16, MEM 16) and Table 1 in the revised manuscript (SAL22057, IPM >16, MEM >16; transconjugant, IPM >16, MEM >16) and between Table 1 and the text in the revised manuscript (Lines 37, 57 SAL22057 IPM 16, MEM 32, Lines 121-2 transconjugant IPM 16, MEM 16).
RESPONSE: We have now conducted a full audit of our raw data and the manuscript to resolve this issue. We have corrected the errors in Table 1. The correct MIC values, as confirmed by our original laboratory records, are as follows: 1. S. Stanley SAL22057: IPM 16 μg/ml; MEM 32 μg/ml. 2. SAL22057-E. coli 600: IPM 16 μg/ml; MEM 16 μg/ml. The values showing ">16" in a previous version of the revised Table 1 were incorrect and resulted from a clerical error during table formatting. The values in the original submission's table and the text (Lines 37-8, 58, 122-123) were, in fact, correct. Table 1 has been updated to reflect the correct MICs as stated above. We have performed a word-by-word verification of the entire Results section to ensure that all MIC values mentioned in the text exactly match the final, corrected Table 1.
NEW COMMENT: Table 1 still has ">16" for IPM, not 16 as stated above, and still has "IMP" instead of IPM.

PREVIOUS COMMENT: Fig. 1 legend - "Inserion sequences" needs correcting, or just use "IS".
RESPONSE: "Inserion sequences" has been adjusted to "IS".
NEW COMMENT: now says "Is" not "IS and this needs correcting. Also suggest "comparing part of" as these are not the complete plasmids.

9) NEW MINOR COMMENTS
Line 64 - this should probably be be "IS26-mediated transposition"? IS26 TU insert most efficiently by transposition.
Lines 105-6 state that the infection was controlled after using MPM. As the SAL22057 was resistant to MPM maybe a short statement about this should be included?
Line 123 - better to say here that the transconjugants had IPM and MEM MICS >32 higher than C600 and "respectively" here means nothing and should be deleted.
Lines 210, 220, 223, 283, 413 - homologous has a specific meaning and can be problematic. Suggest "related plasmids".
Line 213 - again, what is the particular relevance of blaOXA-10?
Line 226 - "IncHI2 or InI1aplha plasmids".
Lines 397, 400 - "confirmed" and "verified" are the wrong words here, as transfer of blaOXA-10 or IncHI2 was not detected.
Line 406 - what is meant by "source relevance"?
Line 414 - is NCBI meant here? If not, what is PCBI?
Line 520 - "sequences of" is not needed.

Reviewer #2 (Comments for the Author):

No further comments

Reviewer #1 (Comments for the Author):

This revised manuscript describes a Salmonella Stanley isolate that has an IncI1 plasmid with blaNDM-13 and an HI2 plasmid that carries many antibiotic resistance genes (ARG), including blaOXA-10. The original version (Spectrum0307-24) stated that these two bla genes were on the same plasmid, but the revised 03207R1 reported that they are actually on separate plasmids. In the response for R2, the authors now agree that blaOXA-10 is not normally considered a carbapenemase in Enterobacterales such as S. Stanley and now state: "the primary focus of our manuscript is the characterization of NDM-13 as the key carbapenemase in Salmonella strain SAL22057". However, blaOXA-10 is still singled out from other ARG on the HI2 plasmid for special mention, and inappropriate descriptions relating to this gene are still used in places. There are also still other problems, including with the selection used in new conjugation experiments for the HI2 plasmid, with descriptions around IS1294 and many other points.

Authors' response: We sincerely appreciate your meticulous review and constructive comments on our revised manuscript during the third round of review. We fully agree with all your suggestions and have carefully made corresponding revisions to address each point. Specifically, we have corrected the misstatement about the location of *bla*$_{NDM-13}$ and *bla*$_{OXA-10}$; we have adjusted the description of *bla*$_{OXA-10}$ to downplay its status, removing any inappropriate emphasis and treating it consistently with other antibiotic resistance genes on the HI2 plasmid. We have also modified other issues

including the selection conditions for HI2 plasmid conjugation experiments and descriptions of IS*1294*, ensuring the manuscript's accuracy and rigor.

MAJOR POINTS

1) blaOXA-10

1.PREVIOUS COMMENT: I still believe that the focus on OXA-10 as a carbapenemase and apparently suggesting that it is equivalent to NDM and other "classic" carbapenemases is not appropriate.

RESPONSE: All references to OXA-10 in the manuscript have been revised to align with the reviewer's accurate assessment. We now state that OXA-10 is primarily a narrow-spectrum β-lactamase and, crucially, that it is not universally classified as a carbapenemase in the same category as NDM, KPC, or OXA-48-like enzymes, especially in Enterobacterales like Salmonella. We acknowledge that its reported carbapenemase activity is weak, host-dependent (as in A. baumannii), and not representative of most alleles. We have strengthened the statement that NDM-13 is the unequivocal and primary determinant of the carbapenem-resistant phenotype in SAL22057, as correctly noted by the reviewer. Instead of framing OXA-10 as a carbapenemase, we now present its presence as part of the broader multidrug-resistant (MDR) arsenal of this isolate (Lines 87-90). We have de-emphasized OXA-10's role regarding carbapenems and reframed its significance to be accurate and consistent with the literature and the reviewer's expert opinion.

NEW COMMENT: If this is now acknowledged, why is there still specific mention of blaOXA-1 above all of the other ARG?

Authors' response: We thank the reviewer for this insightful comment. As we have acknowledged and fully agreed that *bla*$_{OXA-10}$ should not be highlighted or emphasized as a key carbapenem resistance determinant, we have now implemented extensive revisions to remove all specific, prominent mentions of *bla*$_{OXA-10}$ throughout the manuscript. Specifically, we have deleted all descriptions of *bla*$_{OXA-10}$ at the following locations: Line 33 (Abstract), Line 44 (Abstract), Lines 54–55 (Importance), Line 59 (Importance), Line 213 (Results), Lines 237, 241, 252–261 (Discussion), Lines 301–312 (Discussion) and Lines 315, 320–321 (Conclusions). In addition, to ensure that *bla*$_{OXA-10}$ is no longer referred to as one of the "critical resistance genes," we have revised Lines 323–324 (Conclusions) from "The presence of these critical resistance genes in a virulent, multidrug-resistant S. Stanley ST29…"to the following more focused and appropriate statement: "The presence of *bla*$_{NDM-13}$, along with other multidrug resistance determinants, in a virulent, multidrug-resistant S. Stanley ST29 …" With these revisions, *bla*$_{OXA-10}$ is no longer specifically highlighted above other antimicrobial resistance genes, and the manuscript now focuses appropriately on *bla*$_{NDM-13}$ as the primary carbapenem resistance determinant, fully in accordance with the reviewer's expert guidance.

2.PREVIOUS COMMENT: Title - should be updated after changes have been made.

RESPONSE: The updated title is: "Carbapenem Resistance Mediated by blaNDM-13 and Co-occurrence of blaOXA-10 in a Salmonella Stanley ST29 Strain in China". This modification explicitly states that NDM-13 is the primary mediator of the carbapenem-resistant phenotype, while the presence of blaOXA-10 is reported as a

significant co-occurring genetic feature. We have also ensured the manuscript text consistently supports this clarification, particularly in the abstract (Lines 33-4) and discussion (Lines 257-262) sections. We believe this revised title addresses your concern by eliminating potential misunderstanding and more precisely representing our results

NEW COMMENT: Given that the authors now agree that there is no evidence that OXA-10 contributes to carbapenem resistance and their statement that the focus of the manuscript is on blaNDM-13, it's not clear why blaOXA-10 is still mentioned in the title. What makes blaOXA-10 more of a "significant co-occurring genetic feature" than the other ARG on the HI2 plasmid? The title could instead reflect that this is a highly resistant isolate.

Authors' response: We appreciate your comment. Given that *bla*$_{\text{OXA-10}}$ does not contribute to carbapenem resistance and is not more significant than other ARGs, we have removed it from the title. The revised title is: "Carbapenem Resistance Mediated by *bla*$_{\text{NDM-13}}$ in a Highly Drug-Resistant *Salmonella* Stanley ST29 Strain in China"

Also, there are still problems in places around descriptions of blaOXA-10:

3. Line 33 (Abstract) - why is the co-occurrence relevant?

Authors' response: We agree with you that OXA-10 should not be presented as equivalent to NDM in the context of carbapenem resistance. We have revised the sentence to focus on *bla*$_{\text{NDM-13}}$ as the primary determinant of carbapenem resistance, and moved the mention of *bla*$_{\text{OXA-10}}$ to the Results section where it is presented as part of the strain's overall resistance gene profile.

4. Line 40 (Abstract) - why single out blaOXA-10 here? This could state that pSAL22057-OXA has # ARG (and say "including blaOXA-10, if you want to justify why it's called pSAL22057-OXA).

Authors' response: Thank you for your valuable suggestion regarding the description of *bla*$_{OXA-10}$. We agree that singling out *bla*$_{OXA-10}$ in the original sentence could be misleading, as it might imply an undue emphasis on this gene over other resistance determinants present on the same plasmid. In response to your comment, we have revised the sentence to provide a more balanced and accurate description (Lines 39-42). The text now reads: "Whole-genome sequencing and plasmid analysis revealed that *bla*$_{NDM-13}$ was located on plasmid pSAL22057-NDM (IncI1α), while a distinct plasmid, pSAL22057-OXA (IncHI2), harbored multiple antimicrobial resistance genes (ARGs), including *bla*$_{OXA-10}$." The updated sentence now states that pSAL22057-OXA carries multiple ARGs, including *bla*$_{OXA-10}$, which aligns with your recommendation to present the plasmid's resistance gene content more comprehensively. This modification also helps justify the plasmid name "pSAL22057-OXA," as it reflects the presence of *bla*$_{OXA-10}$ among other resistance genes.

5. Line 44 (Abstract) - again, why specifically mention blaOXA-10 here?

Authors' response: Thank you for your continued attention to the presentation of our data. We appreciate your question regarding the specific mention of *bla*$_{OXA-10}$ in the sentence about IS*26* flanking. We agree that singling out *bla*$_{OXA-10}$ in this context could again create an unintended emphasis on a single gene, especially given that

pSAL22057-OXA carries multiple antimicrobial resistance genes (ARGs). In response, we have revised the sentence to reflect the broader role of IS*26* elements in mobilizing multiple resistance genes on this plasmid (Lines 44-47). The text now reads: "Furthermore, the consistent flanking of several resistance genes by IS*26* elements indicates that IS*26*-mediated horizontal gene transfer is a key mechanism driving the dissemination of these determinants and genomic rearrangements within *Enterobacteriaceae*."

6. Line 54-5 (Importance) - blaOXA-10 is only one of several other ARG.

Authors' response: Thank you for your continued careful review and for pointing out that bla_{OXA-10} is only one of several ARGs present in the strain. We agree with you that bla_{OXA-10} should not be highlighted or emphasized in the description of this strain. In response to your comment, we have removed specific mention of bla_{OXA-10} from the relevant sentence to avoid overstating its importance. (Lines 53-4). The revised sentence now reads: "This study identified a carbapenem-resistant *Salmonella Stanley* strain SAL22057 from a pediatric patient, which carried the carbapenemase gene bla_{NDM-13}."

7. Line 59 (Importance) still says "two high-risk carbapenemase genes, blaNDM-13 and blaOXA-10".

Authors' response: Thank you for your continued patience and for pointing out that the sentence still incorrectly classified bla_{OXA-10} as a carbapenemase gene. We agree with your assessment. OXA-10 is not a carbapenemase, and the original phrasing was scientifically inaccurate. To correct this error and avoid any further confusion, we

have decided to delete the sentence entirely from the manuscript.

8. Line 65 (Importance) still uses "OXA-10 type carbapenemases"

Authors' response: Thank you for your continued diligence in reviewing our manuscript. You are absolutely correct: OXA-10 is not a carbapenemase, and referring to it as "OXA-10-type carbapenemases" in the previous version was scientifically incorrect. The updated version simply states that IS*26*-mediated recombination accelerates the spread of resistance genes (Lines 61-3).

9. Lines 323-4 (Conclusions) still state "The presence of these critical resistance genes in a virulent, multidrug-resistant S. Stanley ST29" after discussing blaNDM-13 and blaOXA-10.

Authors' response: Thank you for this careful observation. Consistent with the guidance to de-emphasize *bla*$_{OXA-10}$ and avoid describing it as a critical resistance determinant, the conclusion has been revised to focus primarily on *bla*$_{NDM-13}$ as the key carbapenem resistance gene, with *bla*$_{OXA-10}$ mentioned only as one of several general multidrug resistance determinants rather than a critical gene (Lines 277-80). The revised sentence reads: "The presence of *bla*$_{NDM-13}$, along with other multidrug resistance determinants, in a virulent, multidrug-resistant S. Stanley ST29 strain underscores its pathogenic potential and the urgent need for enhanced surveillance in human and animal populations."

10. PREVIOUS COMMENT Line 54 - I don't think that it can be argued that blaOXA-10 is "critical" in SAL22057.

RESPONSE: The text now reads: "This study identified a carbapenem-resistant

Salmonella Stanley strain, SAL22057, from a pediatric patient, which co-harbored the carbapenemase gene blaNDM-13 and blaOXA-10 β-lactamase gene."

NEW COMMENT: Again, I don't see why blaOXA-10 is still singled out for special attention.

Authors' response: Thank you for this careful and constructive comment. Accordingly, we have removed specific mention of *bla*$_{OXA-10}$ from the relevant sentence to avoid overstating its importance, and only focus on the clinically relevant carbapenemase gene *bla*$_{NDM-13}$ (Lines 53-4). The revised sentence now reads: "This study identified a carbapenem-resistant *Salmonella Stanley* strain SAL22057 from a pediatric patient, which carried the carbapenemase gene *bla*$_{NDM-13}$."

2) New conjugation experiment

11. PREVIOUS COMMENT: It might also be good to look for conjugation of pSAL22057-NDM but different selection conditions would be needed (not necessarily for blaOXA-10, as one of the other resistance genes on the plasmid could be selected for) and noting that HI2 plasmid transfer better at temperatures around 25-30 degrees, rather than 37.

AUTHORS' RESPONSE: We have performed an additional, optimized conjugation experiment as follows: Temperature Adjustment: Based on the well-documented temperature sensitivity of many conjugative systems (including HI2-type plasmids), we repeated the conjugation assay at 25{degree sign}C, 30{degree sign}C and 37{degree sign}C, for 18 hours each, with three independent replicates per condition. To specifically detect the transfer of the plasmid carrying blaOXA-10

(pSAL22057-OXA), we employed a targeted selection strategy. Transconjugants were selected on Mueller-Hinton agar supplemented with rifampicin (200 mg/L, to counter select the donor) and CTX (2 mg/L, to select for the OXA-10 β-lactamase). The Methods 5.5 section (Lines 393-395) now described the optimized conjugation protocol. The relevant Results (Lines 116-26) and Discussion (Lines 274-78) section has been updated accordingly.

NEW COMMENT: I think there is a problem here, as blaOXA-10 does not encode an ESBL (and neither does the only other bla gene on pSAL22057-NDM, blaTEM-1) using CTX would likely fail to select transconjugants that carry it. As mentioned in the comment above it should be possible to select for one of the other ARG on pSAL22057-OXA.

Authors' response: Thank you for this critical correction. We fully agree that $bla_{\text{OXA-10}}$ does not confer resistance to CTX, and selection with CTX is inappropriate for identifying transconjugants carrying pSAL22057-OXA. We have therefore revised the selection strategy to use chloramphenicol (CHL) (32 mg/L) instead, targeting the *floR*, *cmlA1*, and *cmlA5* genes present on the IncHI2-type plasmid pSAL22057-OXA, as recommended. This approach is consistent with established protocols for selecting conjugative IncHI2 plasmids in *Salmonella* and *E. coli*, where CHL is widely used due to its stringent selection and low background. The optimized conjugation protocol (performed at 25°C, 30°C, and 37°C, as advised for HI2 plasmids) has been updated in Methods (Lines 353), the corresponding Results (Lines 119–122), Table 1 and Discussion (Lines 249) sections have been revised to reflect the corrected selection

agent.

3) Lines 125-40 -

12. PREVIOUS COMMENT: this could be condensed by stating the plasmid sizes and the resistance genes that they carry.

RESPONSE: We have condensed the text as suggested by integrating the plasmid sizes with the resistance genes they carry (Lines 140-8).

PREVIOUS COMMENT: - there should not be a space after aac or aph in these gene names.

RESPONSE: We have removed the space after aac or aph in these gene names.

NEW COMMENT: this can still be condensed further by avoiding repetition and combining with Lines 153-4, e.g. "...ARG were identified: an aac(6′)-Iaa gene (intrinsic in Salmonella) on the chromosome, blaNDM-13 and blaMBL on pSAL22057-NDM (IncI1 87,928 bp) and 15 other ARG on pSAL22057-OXA (IncHI2, 237376 bp). The remaining plasmids".

Authors' response: Thank you the detailed and helpful suggestion. We have further condensed the description as recommended, removed repetitive content, and combined the relevant information with Lines 153–154 to improve conciseness and readability (Lines 141-7). The revised text now reads: "A total of 18 ARG were identified: an *aac (6')-Iaa* gene (intrinsic in *Salmonella*) on the chromosome, *bla*$_{\text{NDM-13}}$ and *bla*$_{\text{MBL}}$ on pSAL22057-NDM (IncI1α, 87,928 bp) and 15 other ARG on pSAL22057-OXA (IncHI2, 237,376 bp). The remaining plasmids [⋯]"

13. Lines 149-50 - this is not a complete sentence and is unnecessary. Lines 150-2 are

also unnecessary.

Authors' response: Thank you for pointing this out. The original sentence (Lines 149-50) was indeed incomplete and has now been revised to: The complete sequences of plasmids pSAL22057-NDM (NZ_CP195667) and pSAL22057-OXA (NZ_CP195668) were analyzed (Lines 149-50). We agree with you that this sentence (Lines 150-2) is unnecessary and has been removed to improve the conciseness of the manuscript.

14. Lines 140-2 - the spaces in aac and aph gene names have not been removed. Also "(A)" of tet(A)" should not be in italics.

Authors' response: Thank you for you the careful check. The spaces in all *aac* gene names have been removed as suggested. The aph and tet(A) gene names have been from the manuscript during the revision addressing Comment 12, so these formatting issues are no longer present in the text.

4) pSAL22057-NDM, IS1294

15. PREVIOUS COMMENT: IS1294 is a member of the IS91 family, rolling circle elements that are known to be able to move adjacent regions (see PMID: 10873528). It should be possible to tell fairly confidently if IS1294 was responsible for inserting part of Tn125 into the pSAL22057-NDM, so this needs to be looked at properly. On a quick look, it seems like MN219406 and CP092912 have a clear insertion vs. CP037994, while pSAL22057-NDM has a deletion in this region.

RESPONSE: Comparative visualization of the insertion of IS1294 upstream of ISAba125 resulted in the truncation of the latter, retaining only a 44-bp fragment

(positions 4612-4655) corresponding to the 3'-end of the tnpA gene. The terIS region of IS1294 (4586-4611 bp) is located directly adjacent to the truncated ΔISAba125. Upon integration upstream of ISAba125, IS1294 serves as a key element driving the mobilization of the "IS1294-blaNDM-13 unit" The plasmid pgs_wt5 appears to represent the ancestral state of such plasmids before the acquisition of this unit. In both pNDM13-SR33 and pHNAHS65I-1, the "IS1294-blaNDM-13 unit" is inserted into a region encoding a hypothetical protein located upstream of the proQ gene. This insertion splits the hypothetical protein into two segments, which we refer to here as hp-left and hp-right for clarity. The hp-left segment is 241 bp in length. Sequence analysis reveals that in both pNDM13-SR33 and pHNAHS65I-1, this segment (positions 4713-4953) is directly adjacent to a terIS region (4,954-5,053 bp), with no nucleotides in between. The hp-right segment is 819 bp long and shares a 25-bp repeat with the 3′ end of the trpF gene. Based on this, we speculate that the integration of the "IS1294-blaNDM-13 unit" may have involved homologous recombination with this hypothetical protein. These findings support the notion that the "IS1294-blaNDM-13 unit" exhibits a specific insertion preference in such plasmids, and that the 25-bp homologous sequence likely plays an important role in determining its integration site.

PREVIOUS COMMENT: Line 233 - it's more than IRR - it's 51 bp of the IRR end. Does this segment include the promoter?

RESPONSE: You are correct in pointing out that the region in question comprises not only the IRR sequence but also an adjacent 51 bp segment from the end of the IRR,

which indeed includes part of the promoter region. We acknowledge the inaccuracy in our initial description regarding the IRR boundary. We have now corrected this inaccuracy in the revised version of the manuscript, clarifying the precise sequence composition in this region. Furthermore, we have incorporated additional data from the newly added sample psg_wt5, which has led to a refined interpretation of the results (Lines 161-163). This includes a more detailed analysis of the oriIS/terIS region flanking IS1294 and the truncated state of ISAba125 in this context. These updates and a more comprehensive discussion of the findings are provided in our response to your Question #13 within the point-by-point reply.

NEW COMMENTS (Lines 160-178). This section still has problems and is unnecessarily complex and can be simplified to the main points, with details in the figure.

Authors' response: Thank you for this constructive comment. We have simplified and streamlined this section to focus only on the key findings, removing redundant details and making the description more concise and readable (Lines 157-67). All the specific structural information and nucleotide positions have been retained in Figure 1 and its legend for clarity (Lines 467-76). The revised text is as follows: The primary distinction was that psg_wt5 (lacked a conserved IS*1294*–ΔIS*Aba125*-*bla*$_{NDM-13}$ present in the other homologous plasmids (Figure 1). In pSAL22057-NDM, insertion of IS*1294* into IS*Aba125* generated a 51-bp right end inverted repeat (IRR) of IS*Aba125* that containing the promoter region, with the *terIS* region of IS*1294* located immediately adjacent to this ΔIS*Aba125*. The IS*1294*–ΔIS*Aba125*-*bla*$_{NDM-13}$ module

was inserted into an encoding region of a hypothetical protein gene upstream of *proQ* in pNDM13-SR33 and pHNAHS65I-1, disrupting the coding region. The insertion resulted in the hypothetical protein being split into two segments, designated hp-left and hp-right (details in Figure 1 legend). Notably, in pSAL22057-NDM, insertion of the IS*1294*–ΔIS*Aba125*-*bla*$_{NDM\text{-}13}$ module led to a large deletion compared with pNDM13-SR33 and pHNAHS65I-1.

16. Line 162 - what is "orf"? Is it inside IS1294?

Authors' response: Thank you for this clarification. We confirm that "orf" is inside IS1294. To eliminate ambiguity and streamline the text, we have removed the reference to this "orf" entirely from the main manuscript text. The structure is now described simply as the conserved IS*1294*–ΔIS*Aba125*–*bla*$_{NDM\text{-}13}$ unit, with any additional details about internal ORFs retained in the Figure 1 legend for reference, as requested (Lines 468-9).

17.Line 163 - IS1294 is inserted in ISAba125, not upstream of it - deltaIS1294 is used on

Authors' response: Thank you for this critical correction. We agree that IS*1294* was inserted into IS*Aba125* rather than upstream, which caused the truncation of IS*Aba125*. We have revised the description from "upstream of IS*Aba125*" to "insertion of IS*1294* into IS*Aba125*", aligning with the truncation of IS*Aba125*. (Lines 158-61).

18.Line 162 and "truncation" of ISAba125 is mentioned in the response and text, which agree with this.

Authors' response: We agree with you and have clarified the truncation of IS*Aba125* in the text accordingly (Lines 158-61).

19.Line 164 - this should state that these are the positions in NZ_CP195667 and be for the 51 bp region.

Authors' response: Thank you for this careful correction. We have updated the fragment length to 51 bp and specified the reference genome (NZ_CP195667) for the coordinates (4,612–4,662) (Lines 469-70).

20.Line 164-5 - this still says "a 44 bp fragment corresponding to the 3′ end of the tnpA gene" despite the authors agreeing in their response that the ISAba125 segment is 51 bp (which includes the IR). The wording must reflect this i.e. 51 bp of the IRR (or right end) not the length of the tnpA gene (which is irrelevant) remains and state that the promoter is present, as this is important.

Authors' response: Thank you for the detailed and important clarification. We have revised the description to accurately state that IS*Aba125* was truncated to a 51-bp fragment corresponding to the right-end inverted repeat (IRR) of IS*Aba125*, which contains the promoter. The irrelevant description referring to the 3′ -end of the tnpA gene has been removed (Lines 158-61). The revised sentence now reads: "In pSAL22057-NDM, insertion of IS*1294* into IS*Aba125* generated a 51-bp right end inverted repeat (IRR) of IS*Aba125* that containing the promoter region, with the t*erIS* region of IS*1294* located immediately adjacent to this ΔIS*Aba125*."

21.Lines 165-6 - try and incorporate this information when discussing the truncation to avoid repetition.

Authors' response: Thank you the helpful suggestion. The information about the truncation of IS*Aba125* and the location of the *terIS* region of IS*1294* has been combined into a single sentence to avoid repetition and improve readability (Lines 158-61).

22.Line 167 etc - I don't think that "IS1294-blaNDM-13 unit" needs quotes and it's really IS1294-deltaISAba125-blaNDM-13. Maybe also give the (correct) length of this insert - see comments below.

Authors' response: Thank you for the constructive suggestions. We have removed the quotation marks, corrected the module to IS*1294*–ΔIS*Aba125*–*bla*$_{\text{NDM-13}}$, and simplified the description in the text as recommended (Lines 157-67). Detailed information about this insertion has been moved to the legend of Fig. 1 to avoid redundancy (Lines 468-76).

23.Lines 167-73 - this can be simplified here and the details given in the legend of Fig. 1. Just say that IS1294-deltaISAba125-blaNDM-13 is inseerted in a gene encoding a hypothetical protein.

Authors' response: Thank you for the constructive suggestions. We have minimized the text to state the insertion into a hypothetical protein gene, with all hp-left/hp-right details moved to the figure legend. (Lines 471-3).

24.Line 171 - positions in what? As the two other plasmids are now being discussed, this is not clear.

Authors' response: Thank you for pointing out this ambiguity. The original wording failed to specify the plasmid context of the nucleotide positions, leading to potential

confusion. We have revised text as: The 241-bp hp-left is located at positions 4, 713-4, 953 bp in both pNDM13-SR33 and pHNAHS65I-1, directly adjacent to the oriIS region (4, 954-4, 974 bp). (Lines 471-3).

To further clarify: While pNDM13-SR33 and pHNAHS65I-1 are distinct plasmids, they share an identical starting site and orientation (with *repA* as the first gene in both). BLASTn analysis confirmed that the segment spanning from *copG* to $bla_{\text{NDM-13}}$ (positions 1981–7598 bp in both plasmids) is 100% identical, with no SNPs or indels. As the hp-left element lies within this fully conserved region, its consistent positional annotation across both plasmids is scientifically valid. The full sequence alignment results are provided at the end of this "Response to Reviewers" document for your reference.

25.Line 173 - this doesn't make sense to me. What are the positions corresponding to these 25 bp? Comparison of the IS1294-deltaISAba125-blaNDM-13 region in pNDM13SR-33 with Tn125 (HQ857107) gives a matching region of 1963 bp, none which appears to be present in psg_wt5 that lacks the insertion. I would say that hp-right is 812 bp. This needs to be looked at again and corrected as necessary.

Authors' response: Thank you for the careful comparison and valuable correction. Following your suggestion, we have re-analyzed the sequence alignment and confirmed that the previously described 25-bp repeat is not supported by sequence evidence. Accordingly, we have removed the entire unsupported statement regarding the 25-bp repeat and the incorrect length of hp-right from the manuscript. These changes improve the accuracy and clarity of the text.

26.Lines 174 - this can be simplified - "reported in this study is that" can be deleted and just state that pSAL22057-NDM has an X bp deletion compared with the other two plasmids, putting the detail in the legend of Fig. 1. Fig. 1 also shows a small difference in bleMBL (a 102 by deletion vs. Tn125 and pNDM13SR-33) in pSAL22057-NDM and pHNAHS65I-1, which should be noted in the legend.

Authors' response: Thank you for these helpful suggestions. The description in the main text has been simplified as recommended, with redundant phrases removed. Detailed information regarding the large deletion event in pSAL22057-NDM has been incorporated into the legend of Fig. 1. In addition, the 102-bp deletion within the ble_{MBL} gene, as observed in the figure, has been clearly noted in the legend. All relevant content has been revised and updated accordingly.

5) Shufflon

28.PREVIOUS COMMENT : Lines 112-3 - IncI plasmids have a shufflon that may not be correctly assembled, even from long-read data (see PMID: 28424528). This needs to be looked at and may explain some differences from other IncI1 plasmids.

RESPONSE: To specifically address your concern, we performed the following additional analysis on the pSAL22057-NDM (IncI1α) plasmid: We manually inspected the assembly graph and raw long-read alignments across the putative shufflon region. We confirmed that the assembly in this region is supported by continuous, high-quality long reads with consistent alignment and no breakpoints indicative of a collapse or misassembly. While the repetitive and recombinogenic nature of shufflon loci is well-documented and can pose challenges, our analysis

based on the current data suggests a contiguous and consistent assembly for the specific allele present in our strain. We have now added a brief statement in the revised Results (Section 2.2) and Methods (Section 5.4) to acknowledge this known complexity and to state that our manual validation did not detect evidence of misassembly in this region. The assembly of this plasmid, including its shufflon configuration, is therefore presented as our best representation based on the available sequencing data.

NEW COMMENT: Please add a statement that only a single shufflon arrangement was also confirmed, if this is the case. If not, this should be looked at.

Authors' response: Thank you for this valuable suggestion. We confirmed that only a single shufflon arrangement was present in pSAL22057-NDM.

29.PREVIOUS COMMENT: Line 162 - "pgs_wt5" does not appear to be a complete plasmid.

RESPONSE: We confirm that 'pgs_wt5' corresponds to the complete plasmid pSG_wt5, as designated in the official GenBank record (accession NZ_CP037994). This sequence was resolved using PacBio RSII long-read technology, which robustly generates closed, circular plasmid genomes.

NEW COMMENT: NZ_CP037994 states that it is a linear sequence and the sequence breaks at the shufflon. Shufflons can cause problems even with PacBio data. This needs to be looked at and some justification provided if it is to be described as a complete plasmid.

Authors' response: Thank you for this careful check and important clarification. We

have re-examined the GenBank record for NZ_CP037994 (pSG_wt5) and confirm that the sequence is annotated as a linear contig with a break at the shufflon region. We address this concern in detail below:

1. Analytical focus and role of pSG_wt5

In this study, pSG_wt5 was used exclusively as a reference sequence for comparative genomic analysis (i.e., as an outer ring in our circular genome plots), rather than as a primary subject of investigation for plasmid completeness. Our core focus was on characterizing our newly sequenced plasmid pSAL22057-NDM, and pSG_wt5 was retrieved from GenBank solely to visualize shared synteny and structural differences with pSAL22057-NDM across most of the plasmid backbone.

2. Nature of the sequence break at the shufflon region

We confirm that the GenBank record for NZ_CP037994 indicates a linear assembly with a break at the shufflon region. Re-examination of this sequence shows that the break occurs at the 3' end of the pilV gene, which is part of the well-documented shufflon system[1]. This system spans from the 3' end of pilV to the rci gene and undergoes site-specific DNA rearrangements, a known biological feature that inherently complicates circular assembly—even with long-read technologies such as PacBio. Thus, the linearity of NZ_CP037994 reflects the intrinsic recombinogenic nature of the shufflon, rather than a technical error in sequencing or assembly.

3. Impact on our study conclusions

Critically, the shufflon region is physically separated from the *bla*$_{NDM-13}$ cluster analyzed in this work. The genetic context surrounding *bla*$_{NDM-13}$ is fully intact and

well-aligned between pSAL22057-NDM and pSG_wt5, with no gaps or rearrangements in this region. Outside the shufflon, pSAL22057-NDM and pSG_wt5 share extensive sequence similarity, validating the use of pSG_wt5 as a reliable reference for our comparative analysis.

Therefore, the linearity of NZ_CP037994 at the shufflon does not compromise our structural comparisons or conclusions regarding the $bla_{\text{NDM-13}}$ mobile module. We have clarified the role of pSG_wt5 as a reference sequence in the revised manuscript to avoid any ambiguity about its assembly status.

1. Brouwer, M.S. et al. Shufflons: IncI shufflons: Assembly issues in the next-generation sequencing era. *Plasmid*. **80,** 111–7 (2015)

6) pSAL22057-OXA with blaOXA-10 and other ARG.

30. PREVIOUS COMMENTS: A much clearer/more accurate description of the resistance regions of pSAL22057-OXA is still needed e.g., Line 176 still refers to "the region", which does not make sense, and "integron 3" (Line 181) is unclear without further explanation. The AMR genes currently listed on Lines 133-5 could be stated here instead, indicating which are in the same cassette array and which others are nearby. Could any IS/Tn explain insertions, are there any DR etc. Again, are there any papers associated with the related plasmids listed in this section that might already have information on this that could be consulted and cited. Information on Lines 245-8 need to be in Results not Discussion.

RESPONSE: The description of the pSAL22057-OXA resistance regions has been amended for greater accuracy and clarity (Lines 195-202). The revisions include a

precise explanation of "Integron 3" and a delineation of the resistance gene distribution, specifying their arrangement either within the integron's cassette array or in flanking sequences (Lines 198-200). Furthermore, it is confirmed that no direct repeats (DRs) are associated with this structure.

NEW COMMENTS:

Lines 180-1 - see previous comments and response on average %GC and consider deleting.

Authors' response: Thank you the reminder. As suggested, the average GC content value has been removed from the description of plasmid pSAL22057-OXA (NZ_CP162097) in the iext.

31.Line 190 - correct "cotains".

Authors' response: Thank you for catching this typo. The spelling error “cotains” has been corrected to contains.

32.Line 191 - remove reference to colors here - they are only relevant in the figure legend.

Authors' response: Thank you for the suggestion. The references to colors have been removed from the main text, as these are only appropriate in the figure legend.

33.Line 192 - this detail is not needed in the text - just show all IS on Fig. 2.

Authors' response: Thank you for the suggestion. The detailed list of mobile genetic elements has been removed from the main text, and all IS elements had already been presented in Fig.

34.Line 196 - the name is "lnuF" (starts with lowercase L) not InuF and "(F)" should

not be in italics.

Authors' response: Thank you for the suggestion. The gene name has been corrected from "Inu(F)" to the correct lnuF, and the italicization of the suffix "(F)" has been removed as recommended (Line 180).

35.Lines 197-7 - spaces in aac and aph gene names need to be removed.

Authors' response: Thank you for the correction. All unnecessary spaces in the aac and aph gene names have been removed to follow standard nomenclature (Lines 181, 183).

36.Line 198 - "intergenic" not needed.

Authors' response: Thank you for the suggestion. The word "intergenic" has been removed from the text as recommended.

37.Line 199-201 - what is meant by a "core cassette array? "core" can be deleted "(A)" of tet(A) should not be in italics and tnpA of what - this must be stated. Should be "which contains blaOXA-10, is flanked". "b" of blaOXA-1 seems to be underlined and "gene" can be deleted.

Authors' response: Thank you for these detailed and helpful suggestions. The word "core" has been removed from "core cassette array"; the italicization of "(A)" in tet(A) has been corrected; the gene "tnpA" has been clarified to "tnpA of Tn*2*"; the phrase "which contains *bla*$_{OXA-10}$, is flanked" has been revised for grammar; the unnecessary word "gene" has been deleted; and the formatting of *bla*$_{OXA-10}$ has been corrected (Line 183).

38.Line 202 - there is an extra "t" that needs to be deleted.

Authors' response: Thank you for the suggestion. The extra "t" has been deleted .

39.Line 203 - "transposition unit' is normally used for segments moved by a single copy of ISEcp1 and related elements. What is meant here?blaNDM-5 shouldnot be described as being in a cassette due to possible confusion with gene cassettes.

Authors' response: Thank you for this important clarification. The term "transposition unit" has been replaced with "IS*3000*-associated transposable region" to avoid confusion, and "*bla*$_{NDM-5}$ cassette" has been revised to "*bla*$_{NDM-5}$ gene" to prevent misunderstanding with integron gene cassettes (Lines 187-8).

7) Discussion and Conclusion

40.PREVIOUS COMMENT: Parts of the Discussion still repeat Results and it would need to be rewritten after changes to the rest of the manuscript.

RESPONSE: We agree that an effective Discussion should interpret and contextualize the findings rather than reiterate the Results. In revising the manuscript based on all previous comments, we have thoroughly rewritten the Discussion section (Lines 232-7, 241-3, 253-9, 264-6, 275-81，284-317) to address this specific point.

NEW COMMENT: There is still repetition of Results in Discussion and problems in this section and Conclusions also repeat Discussion.

Authors' response: Thank you for the continued feedback. which has been instrumental in refining the clarity and rigor of our manuscript. We have thoroughly revised the Discussion and Conclusions sections to eliminate repetition of Results, correct factual errors, and strengthen interpretive content, as detailed below:

1. We have removed all direct restatements of Results (e.g., "pSG_WT5 lacks the

IS*1294* unit" "pSAL22057-NDM lost four ORFs") from the Discussion.

2. The Discussion now focuses exclusively on interpreting structural differences, contextualizing findings with existing literature, and discussing mechanistic implications (e.g., IS*1294*-mediated promoter capture, IS*26*-mediated HGT).

3. The Conclusions section has been completely rewritten to distill only the core novel findings, avoiding any repetition of Discussion details and emphasizing the broader clinical and evolutionary significance of our work.

41.Lines 282-300 (IS1294) - much of this repeats Results and parts are incorrect - see comments above relating to IS1294 and the 25 bp region.

Authors' response: Thank you for this critical feedback. We have thoroughly revised this section to eliminate repetition of Results, correct factual errors, and strengthen interpretive content, as detailed below:

1 **Elimination of Results repetition:** We have removed all direct restatements of Results (e.g., "the conserved genetic structure 'IS*1294-bla*$_{\mathrm{NDM-13}}$ unit' was absent in psg_wt5" "the integration in pSAL22057-NDM was associated with the loss of four ORFs") and refocused the text on interpreting the functional and evolutionary implications of our findings.

2 **Correction of factual errors**: We have revised the inaccurate term "IS*1294-bla*$_{\mathrm{NDM-13}}$ unit" to the precise "IS*1294*–ΔIS*Aba125*–*bla*$_{\mathrm{NDM-13}}$ mobile module" and removed unnecessary quotation marks. We have corrected the insertion site description from "upstream of IS*Aba125*" to reflect its integration within IS*Aba125*, consistent with the truncation of IS*Aba125* to a 51-bp IRR fragment

(including the promoter region). We have removed the unsubstantiated hypothesis of a 25-bp homologous sequence mediating recombination, replacing it with a discussion of IS*1294*'s inherent target site preference, which is supported by comparative sequence analysis.

Strengthened interpretation: The revised text now emphasizes the role of IS*1294* as a driver of *bla*$_{NDM-13}$ mobilization, the genomic plasticity evident in the pSAL22057-NDM deletion event, and the target site specificity of the mobile module, aligning with the purpose of the Discussion section to contextualize findings rather than repeat data.

The updated section now accurately reflects our data and adheres to the academic standard of interpreting results rather than restating them.

42.Lines 301-12 - this also still repeats Results. Horizontal gene transfer is between cells. Is there real evidence about acquisition of these modules?

Authors' response: Thank you for this critical feedback. As suggested, we have removed the entire paragraph (Lines 301–312) to avoid repetition of Results and to address the concern regarding the lack of direct evidence for the acquisition of these modules via horizontal gene transfer.

Minor points

43.Line 233- " context" is not needed here.

Authors' response: Thank you for your suggestion. The "context" is removed in the revised manuscript.

44.Lines 237, 241, 252-61 - again, if the focus is now blaNDM-13, why single out

blaOXA-10 for special mention?

Authors' response: Thank you for this observation. We agree that the original text overemphasized *bla*$_{OXA-10}$ relative to the core focus on *bla*$_{NDM-13}$. Removed all paragraphs related to *bla*$_{OXA-10}$ entirely, as they are not central to the study's focus on the mobilization and characterization of the *bla*$_{NDM-13}$-containing plasmid.

45.Lines 262- might be better at the start of the Discussion.

Authors' response: Thank you for the helpful suggestion. The sentence regarding the first identification of the *bla*$_{NDM-13}$ gene has been placed at the beginning of the Discussion section, and the surrounding text has been slightly adjusted to ensure logical coherence and smooth transition (Lines 237-8). All revisions are highlighted in the revised manuscript.

46.Line 266 - not clear what "its" is here Specify blaNDM-13.

Authors' response: We appreciate the suggestion. The pronoun "its" has been replaced with "*bla*$_{NDM-13}$" to clarify the reference, and the revision is marked in the revised manuscript (Lines 240).

47.Lines 268-272 - "The" is not needed before plasmid names or "transfer". "the complete plasmid sequences of" is not needed.

Authors' response: Thank you for the careful correction. The unnecessary definite article "The" preceding plasmid names has been removed, and the redundant phrase "the complete plasmid sequences of" has been deleted to streamline the expression.

48.Line 271 – plasmids

Authors' response: Thank you for the careful correction. The singular noun "plasmid"

has been revised to the plural "plasmids" to match the subject.

49.Lines 278-80 - odd "1" at start of Line 278, "represent", not represents.

Authors' response: Thank you for the careful correction. The erroneous character "1" has been removed, and the verb "represents" has been revised to "represent" to follow the grammatical rule with the modal verb "may".

50.Line 301 - "characterized" is the wrong word here.

Authors' response: Thank you for the careful correction. In response to the concern about repetition of Results and the lack of direct evidence for the speculative acquisition of modules via horizontal gene transfer, we have removed the entire paragraph (Lines 301–312). The inappropriate word "characterized" has been deleted.

51.Line 308 - "in is" needs fixing.

Authors' response: Thank you for the careful correction. The typo "in is" has been deleted.

52.Line 315, 320-1 - again, why mention blaOXA-10?

Authors' response: Thank you for your comment. Completely removed all mentions of *bla*$_{OXA-10}$ from the Conclusion and manuscript, to focus solely on our core finding of *bla*$_{NDM-13}$.

53.Lines 316-7 don't really say anything meaningful.

Authors' response: Thank you for your feedback. Deleted the empty, non-specific statements in Lines 316–317, replacing them with a focused summary of our mechanistic insights into blaNDM-13 mobilization.

54.Line 321 - "This implicating" doesn't make sense, IS26 directly mediates only

intracellular transfer, not horizontal gene transfer, and this role was well known before this study.

Authors' response: Thank you for your comments. The entire sentence has been removed, which resolves the grammatical error and the inaccurate description of IS26 function, while further streamlining our conclusion.

8) MINOR POINTS RELATING TO PREVIOUS COMMENTS

55.PREVIOUS COMMENT: Line 40 - this should be IncI1alpha, not IncI1Ialpha

RESPONSE: We have rephased "IncI1Ialpha" as "IncI1alpha".

NEW COMMENT: This is still wrong on Line 60.

Authors' response: The relevant text has already been removed in response to a previous comment (Comment 7), so this nomenclature issue is no longer present in the manuscript.

56.PREVIOUS COMMENT: Lines 78-9 - the wording here needs to be made more accurate and more grammatically correct. It should be "the blaKPC, ... or blaOXA-48-like genes", but "and/or" should be used instead of "or" if any have examples have >1 of these genes, and "like" should not be subscript.

RESPONSE: We have revised the sentence to accurately reflect the performed. The text now reads: "the blaKPC, blaIMP, blaNDM, blaVIM, and/or blaOXA-48-like" (Line 79).

NEW COMMENT: This should be "blaOXA-48-like genes", as noted in the previous comment. "blaOXA-48-like without "genes" after it is not good English.

Authors' response: Thank you for your reminder and correction. The phrase has been

revised to “*bla*$_{OXA-48}$-like genes” to improve grammatical accuracy, as suggested.

57.PREVIOUS COMMENT: Line 82 - words missing.

RESPONSE: The corrected now reads: the ability of these enzyme's hydroze β-lactams is unaffected by available β-lactamase inhibitors (e.g., avibactam, relebactam, and vaborbactam) (Lines 82-3).

NEW COMMENT: "enzyme's" should be "enzymes" and "to" is needed before hydolyze and there is no space between "lactams" and "is" in the manuscript.

Authors’ response: Thank you for identifying this grammatical error. The missing structure has been corrected, and the sentence now reads: “The ability of these enzymes to hydrolyze *β*-lactams is unaffected by available *β*-lactamase inhibitors (e.g., avibactam, relebactam, and vaborbactam).

58.PREVIOUS COMMENT: Line 136 - "the plasmid" is not needed here - the "p" in the name indicates this. Also check and remove/reword throughout e.g. Lines 160, 161, figure legends.

RESPONSE: All mentions of "the plasmid" have been removed. We have also checked the entire manuscript and applied these changes to Lines 160-161 and the figure legends.

Lines 174, 285-6 - still say "The plasmid p..." - please check again.

Authors’ response: The relevant text has already been removed in response to a previous comment (Comment 26,41), so this nomenclature issue is no longer present in the manuscript.

59.PREVIOUS COMMENT: Line 219 - "it is worth noting that" "are classified", "is

carried", as all of these things are still true.

RESPONSE: We have revised it in the manuscript as suggested ... and use the present tense for stating current scientific facts. The sentence now reads: The pHNAHS65I-1 and pNDM13-SR33 are classified as IncI1 plasmids originating from China.

NEW COMMENT: There are still problems throughout with tenses.

Authors' response: Thank you for noting the inconsistent tense usage. The sentence has been revised to use present tense for consistent and appropriate description of objective findings: "PHNAHS65I-1 and pNDM13-SR33 are classified as IncI1-type plasmids originating from China."(Liens 244-5) We have also checked the entire manuscript for consistent tense usage and revised accordingly.

60.PREVIOUS COMMENT: Lines 314-5 - wording does not make sense.

RESPONSE: We have rephrased the sentence to improve clarity, as suggested:

"A search for transposon and IS elements was performed using the IS Finder database (https://www-is.biotoul.fr/)."(Lines 385-6)

NEW COMMENT: Change to "transposons and IS" (but note that the ISFinder database only includes a few selected transposons).

Authors' response: Thank you for the helpful suggestion. The phrase has been revised to "transposons and IS elements" as recommended. We have also added a note indicating that the ISFinder database includes only a limited set of selected transposons, in keeping with the reviewer's comment. The updated sentence reads: "A search for transposons and IS elements was performed using the ISFinder database (https://www-is.biotoul.fr/), which contains only a small number of selected

transposons.”

61.PREVIOUS COMMENT: Lines 330-331 - in addition to plasmids, the term "mobile genetic elements" encompasses IS and transposons, which would not be detected by oriTfinder, so is incorrect here.

RESPONSE: We have rephased as "The conjugation probability of pSAL22057-NDM and pSAL22057-OXA were identified by the oriTfinder (https://bioinfo-mml.sjtu.edu.cn/oriTfinder/) (402-4).

NEW COMMENT: "conjugation probability" is still not ideal. Maybe state what can be detected by oriFinder here?

Authors’ response: We appreciate your comment. The non-ideal term “conjugation probability” has been replaced, and the sentence now correctly describes the function of oriTfinder for predicting oriT regions (Lines 375-6).

62.PREVIOUS COMMENT (bla gene PCR Lines 351-2): The text needs to state which genes would be detected by the primers used.

RESPONSE: The carbapenemase genes (blaNDM, blaKPC, blaVIM-1, blaIMP-4, and blaOXA-48-like) were detected by the primers used. We have stated this in revised manuscript (352-3).

NEW COMMENT: should this actually be "blaIMP-4-like" and "blaVIM-1-like" genes? i.e. minor variants in these subfamilies would also be detected? Note that "-like" should not be subscript and needs to be followed by "genes" - see comment above.

Authors’ response: Thank you for these important clarifications and corrections.

The gene nomenclature has been revised to *bla*$_{VIM-1}$-like, *bla*$_{IMP-4}$-like to reflect that the PCR assay detects members of these subfamilies rather than only specific variants. The formatting of "-like" has been corrected to avoid subscripting, and the word "genes" has been added for grammatical accuracy. The revised sentence reads: "The carbapenemase genes (*bla*$_{NDM}$, *bla*$_{KPC}$, *bla*$_{VIM-1}$-like, *bla*$_{IMP-4}$-like, and *bla*$_{OXA-48}$-like genes) were detected by PCR-based sequencing, as previously described."

63.PREVIOUS COMMENT: The versions of the plasmid sequences currently in GenBank are still not annotated. Maybe they are waiting to be updated? If not, they should be annotated.

RESPONSE: The plasmid sequences have been annotated. Please refer to the following link:

1.https://www.ncbi.nlm.nih.gov/nuccore/NZ_CP162099.2/

2.https://www.ncbi.nlm.nih.gov/nuccore/NZ_CP162098.2/

3.https://www.ncbi.nlm.nih.gov/nuccore/NZ_CP195668.1/

4.https://www.ncbi.nlm.nih.gov/nuccore/NZ_CP195669.1/

5.https://www.ncbi.nlm.nih.gov/nuccore/NZ_CP195667.1/

6.https://www.ncbi.nlm.nih.gov/nuccore/NZ_CP162097.2/

NEW COMMENT: Lines 436-441 - it seems that e.g. CP162097 is not annotated but NZ_CP162097 is, so maybe the NZ_ accessions should be listed here?

Authors' response: Thank you for this careful observation and correction. We confirm that the sequences are available under the NZ_ prefixed RefSeq accession numbers in the NCBI database. We have revised the text to list the complete, standardized RefSeq

accessions (e.g., NZ_CP162097 instead of CP162097) to ensure accurate and direct traceability of the sequence data (Lines 394-7). The updated accession list is as follows: Chromosome: NZ_CP162096; pSAL22057-OXA: NZ_CP162097; pSAL22057-4: NZ_CP162098; pSAL22057-3: NZ_CP162099; pSAL22057-NDM: NZ_CP195667; pSAL22057-5: NZ_CP195668; pSAL22057-6: NZ_CP195669.

64.PREVIOUS COMMENT: References: there are still problems with missing details, journal names not correctly abbreviated etc and some citations seem to have been mixed up (see comments above).

RESPONSE: We have now carefully checked and revised the entire reference list accordingly. The missing details have been added, all journal names have been corrected to their standard abbreviations, and any mixed-up citations have been identified and properly placed. The reference section has been thoroughly updated to ensure accuracy and consistency.

NEW COMMENTS:

Ref 3 - should be "lactamase" with lower case "l".

Ref 4 - should be "producing with lowercase "p".

Refs 7, 14 - change format of title from Title Case to Sentence case

Ref 8 - blaNDM-13 is not correctly formatted.

Authors' response: Thank you for carefully reviewing the reference formatting. We have revised all references to correct capitalization, title case, and gene nomenclature issues as follows:

1.Ref 3: Changed "β-Lactamases" to "β-lactamases".

2.Ref 4: Adjusted title to sentence case, changing "Resistant", "Producing", and "Resistance" to lowercase.

3.Ref 7: Converted titles from Title Case to Sentence Case.

4.Ref 8: Corrected "bla NDM-13" to "bla_{NDM-13}" to follow standard gene formatting.

5.Ref 14 has been removed entirely from the reference list in line with our response to Comment 44, so the title formatting issue for this reference is no longer present.

All revisions have been applied to the reference list in the manuscript.

65.PREVIOUS COMMENT - there are inconsistencies in the MICs of IPM and MEM between Table 1 in the original manuscript (SAL22057, IPM 16, MEM 32; transconjugant, IPM 16, MEM 16) and Table 1 in the revised manuscript (SAL22057, IPM >16, MEM >16; transconjugant, IPM >16, MEM >16) and between Table 1 and the text in the revised manuscript (Lines 37, 57 SAL22057 IPM 16, MEM 32, Lines 121-2 transconjugant IPM 16, MEM 16).

RESPONSE: We have now conducted a full audit of our raw data and the manuscript to resolve this issue. We have corrected the errors in Table 1. The correct MIC values, as confirmed by our original laboratory records, are as follows: 1. S. Stanley SAL22057: IPM 16 μg/ml; MEM 32 μg/ml. 2. SAL22057-E. coli 600: IPM 16 μg/ml; MEM 16 μg/ml. The values showing ">16" in a previous version of the revised Table 1 were incorrect and resulted from a clerical error during table formatting. The values in the original submission's table and the text (Lines 37-8, 58, 122-123) were, in fact, correct. Table 1 has been updated to reflect the correct MICs as stated above. We have performed a word-by-word verification of the entire Results section to ensure that all

MIC values mentioned in the text exactly match the final, corrected Table 1.

NEW COMMENT: Table 1 still has ">16" for IPM, not 16 as stated above, and still has "IMP" instead of IPM.

Authors' response: We appreciate your careful observation. The discrepancies in Table 1 have been fixed: the IPM MIC value has been updated from ">16" to "16", and the abbreviation "IMP" has been corrected to "IPM". The revised table is included in the manuscript.

66.PREVIOUS COMMENT: Fig. 1 legend - "Inserion sequences" needs correcting, or just use "IS".

RESPONSE: "Inserion sequences" has been adjusted to "IS".

NEW COMMENT: now says "Is" not "IS and this needs correcting. Also suggest "comparing part of" as these are not the complete plasmids.

Authors' response: We appreciate your careful check. The lowercase "Is" has been corrected to "IS", and the legend has been updated to "comparing part of" the plasmids to note that only partial sequences are displayed. The revised Fig. 1 legend is included in the manuscript.

9) NEW MINOR COMMENTS

67.Line 64 - this should probably be be "IS26-mediated transposition"? IS26 TU insert most efficiently by transposition.

Authors' response: Thank you for this precise suggestion. The term "recombination" has been revised to "transposition" to accurately reflect the primary mechanism of IS*26* movement, as recommended.

68.Lines 105-6 state that the infection was controlled after using MPM. As the SAL22057 was resistant to MPM maybe a short statement about this should be included?

Authors' response: Thank you for this important comment. A brief explanation has been added to address the apparent discrepancy between the in vitro MEM resistance of SAL22057 and the favorable clinical outcome. As suggested, we note that the successful control of infection may have been achieved through a prolonged treatment course (Lines 104-7). The revised sentence reads: "After 8 days of MEM treatment, body temperature normalized and the infection was controlled. Given that the isolate SAL22057 was resistant to MEM in vitro, the favorable clinical outcome may have been achieved by a prolonged treatment course."

69.Line 123 - better to say here that the transconjugants had IPM and MEM MICS >32 higher than C600 and "respectively" here means nothing and should be deleted.

Authors' response: Thank you for this suggestion. The word "respectively" has been removed as it was unnecessary in this context. The sentence has been revised to more clearly state that the IPM and MEM MICs of the transconjugants were more than 32-fold higher than those of the recipient *E. coli* C600 (Lines 124-6).The revised sentence reads: "The IPM and MEM MICs of the transconjugants were more than 32-fold higher than those of the recipient *E. coli* C600."

70.Lines 210, 220, 223, 283, 413 - homologous has a specific meaning and can be problematic. Suggest "related plasmids".

Authors' response: We thank the reviewer for this important clarification regarding precise terminology. The term "homologous" has been replaced with "related" at lines 210, 220, 223, and 413 throughout the manuscript, as suggested, to avoid potential misinterpretation. As addressed in our response to Comment 41, the term "homologous" at Line 283 has been deleted and is no longer used.

71.Line 213 - again, what is the particular relevance of blaOXA-10?

Authors' response: We thank the reviewer for this comment. In line with the previous suggestion to de-emphasize *bla*$_{OXA-10}$, the sentence highlighting co-occurrence of *bla*$_{NDM-13}$ and *bla*$_{OXA-10}$ has been removed entirely, to avoid overstating the relevance of *bla*$_{OXA-10}$.

72.Line 226 - "IncHI2 or InI1aplha plasmids".

Authors' response: We thank the reviewer for this helpful suggestion. The phrase has been revised to "IncHI2 or IncI1alpha plasmids" to improve clarity and accuracy, as recommended (Lines 209-210). The revised sentence reads: "IncHI2 or IncI1α plasmids were detected in the genomic sequences of multiple reference ST29 strains."

73.Lines 397, 400 - "confirmed" and "verified" are the wrong words here, as transfer of blaOXA-10 or IncHI2 was not detected.

Authors' response: Thank you for this important correction regarding precise wording. As transfer of *bla*$_{OXA-10}$ or IncHI2 was not detected, the terms "confirmed" and "verified" have been replaced with more appropriate wording describing the detection and assessment of plasmid presence and gene transfer potential (Lines 253-7). The revised sentence reads: "To investigate the potential horizontal transfer of *bla*$_{NDM-13}$

and bla_{OXA-10}., three transconjugants from each condition were analyzed by PCR and antimicrobial susceptibility testing. Replicon typing was performed to assess the presence of IncI1α and IncHI2 plasmids in the transconjugants."

74.Line 406 - what is meant by "source relevance"?

Authors' response: We thank the reviewer for this comment. The phrase "source relevance" has been clarified as "matching strain or plasmid background", referring to sequences from closely related strain types and plasmid backgrounds relevant to this study, to improve precision and clarity (Line 362). The revised sentence reads: "Reference sequences for each fragment were chosen based on high similarity (≥95% identity, >90% coverage), matching strain or plasmid background, and completeness, with final validation through Easyfig alignment."

75.Line 414 - is NCBI meant here? If not, what is PCBI?

Authors' response: Thank you for catching this typographical error. PCBI-Pathogen was a misspelling of NCBI Pathogen Detection; the text has been corrected to reflect the correct database name.

76.Line 520 - "sequences of" is not needed.

Authors' response: Thank you for suggestion. The "sequences of" has been removed.

BLASTN 2.13.0+

Reference: Zheng Zhang, Scott Schwartz, Lukas Wagner, and Webb Miller (2000), "A greedy algorithm for aligning DNA sequences", J Comput Biol 2000; 7(1-2):203-14.

Database: User specified sequence set (Input: .\pHNAHS65I-1_copG-blaNDM-13.fasta).
1 sequences; 5,618 total letters

Query= pNDM13-SR33_copG-blaNDM-13

Length=5618

```
                                                              Score     E
Sequences producing significant alignments:                   (Bits)  Value

pHNAHS65I-1_copG-blaNDM-13                                    10375   0.0
```

> pHNAHS65I-1_copG-blaNDM-13
Length=5618

Score = 10375 bits (5618), Expect = 0.0
Identities = 5618/5618 (100%), Gaps = 0/5618 (0%)
Strand=Plus/Plus

```
Query  1    ATGCAAATGAAAAACAATACCGCACAGGCAACAAAAGTAATTACCGCGCATGTGCCATTA  60
            ||||||||||||||||||||||||||||||||||||||||||||||||||||||||||||
Sbjct  1    ATGCAAATGAAAAACAATACCGCACAGGCAACAAAAGTAATTACCGCGCATGTGCCATTA  60

Query  61   CCTATGGCTGATAAAGTCGACCAGATGGCCGCCAGACTGGAACGCTCCCGGGGCTGGATT  120
            ||||||||||||||||||||||||||||||||||||||||||||||||||||||||||||
Sbjct  61   CCTATGGCTGATAAAGTCGACCAGATGGCCGCCAGACTGGAACGCTCCCGGGGCTGGATT  120

Query  121  ATCAAACAGGCGCTTTCTGCATGGCTTGCCCAGGAGGAGGAGCGTAATCGCCTGACGCTG  180
            ||||||||||||||||||||||||||||||||||||||||||||||||||||||||||||
Sbjct  121  ATCAAACAGGCGCTTTCTGCATGGCTTGCCCAGGAGGAGGAGCGTAATCGCCTGACGCTG  180

Query  181  GAAGCCCTGGACGATGTGACATCCGGACAGGTTATCGACCATCAGGCTGTACAGGCCTGG  240
            ||||||||||||||||||||||||||||||||||||||||||||||||||||||||||||
Sbjct  181  GAAGCCCTGGACGATGTGACATCCGGACAGGTTATCGACCATCAGGCTGTACAGGCCTGG  240

Query  241  GCGGACAGCCTCAGTACTGACAATCCGTTACCGGTGCCACGCTGATGGAACTGAAGTGGA  300
            ||||||||||||||||||||||||||||||||||||||||||||||||||||||||||||
```

Sbjct 241 GCGGACAGCCTCAGTACTGACAATCCGTTACCGGTGCCACGCTGATGGAACTGAAGTGGA 300

Query 301 CCAGTAAGGCGCTTTCTGATTTGGCGCGGTTATATGATTTTCTGGTGCTGGCCAGTAAAC 360
||||||||||||||||||||||||||||||||||||||||||||||||||||||||||||
Sbjct 301 CCAGTAAGGCGCTTTCTGATTTGGCGCGGTTATATGATTTTCTGGTGCTGGCCAGTAAAC 360

Query 361 CTGCGGCCGCCAGAACGGTGCAGTCCCTGACACAGGCTCCGGTCATTCTGTTAACTCATC 420
||||||||||||||||||||||||||||||||||||||||||||||||||||||||||||
Sbjct 361 CTGCGGCCGCCAGAACGGTGCAGTCCCTGACACAGGCTCCGGTCATTCTGTTAACTCATC 420

Query 421 CACGTATGGGGGAACAGTTATTTCAGTTTGAACCCAGGGAGGTCAGACGGATTTTTGCTG 480
||||||||||||||||||||||||||||||||||||||||||||||||||||||||||||
Sbjct 421 CACGTATGGGGGAACAGTTATTTCAGTTTGAACCCAGGGAGGTCAGACGGATTTTTGCTG 480

Query 481 GCGAGTACGAAATCCGTTACGAACTTACTGGCCAGACTATTTATGTATTGCGCCTGTGGC 540
||||||||||||||||||||||||||||||||||||||||||||||||||||||||||||
Sbjct 481 GCGAGTACGAAATCCGTTACGAACTTACTGGCCAGACTATTTATGTATTGCGCCTGTGGC 540

Query 541 ACACACGAGAAAACAGGTAGATTGTGTGGGGACAACTTAACATAGCCTGCAGATATTA 600
||||||||||||||||||||||||||||||||||||||||||||||||||||||||||||
Sbjct 541 ACACACGAGAAAACAGGTAGATTGTGTGGGGACAACTTAACATAGCCTGCAGATATTA 600

Query 601 ATTCTGGAACATTATTATCAGGCCAGATATTTTGCATTCCATTAAATAATCTTGTAGGAG 660
||||||||||||||||||||||||||||||||||||||||||||||||||||||||||||
Sbjct 601 ATTCTGGAACATTATTATCAGGCCAGATATTTTGCATTCCATTAAATAATCTTGTAGGAG 660

Query 661 ATAAAATGAATTACGATGAAATAACGAAAATTACCGCTGAACGTATTAGCGACTACATGA 720
||||||||||||||||||||||||||||||||||||||||||||||||||||||||||||
Sbjct 661 ATAAAATGAATTACGATGAAATAACGAAAATTACCGCTGAACGTATTAGCGACTACATGA 720

Query 721 CTGAAGCAGTTAATACAGACTCTATAGCCGTTGCGGAAATGTTTCATAACGCTGCATGGG 780
||||||||||||||||||||||||||||||||||||||||||||||||||||||||||||
Sbjct 721 CTGAAGCAGTTAATACAGACTCTATAGCCGTTGCGGAAATGTTTCATAACGCTGCATGGG 780

Query 781 GAGTGCGAACATTGTGGTTTGAACTCGTCACAAAAATAGATATAGATATACATAAAAAGA 840
||||||||||||||||||||||||||||||||||||||||||||||||||||||||||||
Sbjct 781 GAGTGCGAACATTGTGGTTTGAACTCGTCACAAAAATAGATATAGATATACATAAAAAGA 840

Query 841 ACAGGTATGCGAGTTATGATCTTAGAAGGAAAATCGAAATGCAGCATGAAGAGTTTCAGA 900
||||||||||||||||||||||||||||||||||||||||||||||||||||||||||||
Sbjct 841 ACAGGTATGCGAGTTATGATCTTAGAAGGAAAATCGAAATGCAGCATGAAGAGTTTCAGA 900

Query 901 AAATGACAGAGCGCGAGCAAGTGCCTTTATTGAAATCGCCAGAATAATAGAAACAGAACA 960
||||||||||||||||||||||||||||||||||||||||||||||||||||||||||||
Sbjct 901 AAATGACAGAGCGCGAGCAAGTGCCTTTATTGAAATCGCCAGAATAATAGAAACAGAACA 960

Query 961 AGTACATTGCTGTATTGTTTTTTCCTTGAATATTGAATCGCGGTGCCCATGTACTACGAT 1020
||||||||||||||||||||||||||||||||||||||||||||||||||||||||||||
Sbjct 961 AGTACATTGCTGTATTGTTTTTTCCTTGAATATTGAATCGCGGTGCCCATGTACTACGAT 1020

```
Query  1021   TCCTTTCGTGTCATTTATCATTGCACACTTTCCCTGACTGAGCGTAAAACACTGACTACT   1080
              ||||||||||||||||||||||||||||||||||||||||||||||||||||||||||||
Sbjct  1021   TCCTTTCGTGTCATTTATCATTGCACACTTTCCCTGACTGAGCGTAAAACACTGACTACT   1080

Query  1081   TTCCCCTCAAACTCCTGGACGGCGAATGCAAAATTGGTCTGTTCCAGCCTAGAATATGTT   1140
              ||||||||||||||||||||||||||||||||||||||||||||||||||||||||||||
Sbjct  1081   TTCCCCTCAAACTCCTGGACGGCGAATGCAAAATTGGTCTGTTCCAGCCTAGAATATGTT   1140

Query  1141 ACTTTTTCATGACCGAACCCGAAATTTTGCGCGTCAACAAGAGACGTTGTGTAAACTCAG   1200
              ||||||||||||||||||||||||||||||||||||||||||||||||||||||||||||
Sbjct  1141   ACTTTTTCATGACCGAACCCGAAATTTTGCGCGTCAACAAGAGACGTTGTGTAAACTCAG   1200

Query  1201   TCCTTTTATCTGGCAGGTTAACCCCTATCAATCTTAGGATGAAACCACGCCATCCTCTGT   1260
              ||||||||||||||||||||||||||||||||||||||||||||||||||||||||||||
Sbjct  1201   TCCTTTTATCTGGCAGGTTAACCCCTATCAATCTTAGGATGAAACCACGCCATCCTCTGT   1260

Query  1261   TTGCCTTGTTATTAATTTCATAAAGACGATTGTTTTCCTCTCGTAGTTTTGTCCACTCTA   1320
              ||||||||||||||||||||||||||||||||||||||||||||||||||||||||||||
Sbjct  1261   TTGCCTTGTTATTAATTTCATAAAGACGATTGTTTTCCTCTCGTAGTTTTGTCCACTCTA   1320

Query  1321   AACAAAGTGTTATATAATCCAGTTCTGTGATGTGTTTCACTTTTCCATTGACAGTTACAG   1380
              ||||||||||||||||||||||||||||||||||||||||||||||||||||||||||||
Sbjct  1321   AACAAAGTGTTATATAATCCAGTTCTGTGATGTGTTTCACTTTTCCATTGACAGTTACAG   1380

Query  1381   TGTTTATTCCATTTCCTGTGATCATCATCTTATGCCTTTTTTATCTTCATTTGGGACCGT   1440
              ||||||||||||||||||||||||||||||||||||||||||||||||||||||||||||
Sbjct  1381   TGTTTATTCCATTTCCTGTGATCATCATCTTATGCCTTTTTTATCTTCATTTGGGACCGT   1440

Query  1441   GCTTTTCTTCGGAGTATCATTATTCGTTAACTGCTGTTTATCAATAAAATTCCTGATGTT   1500
              ||||||||||||||||||||||||||||||||||||||||||||||||||||||||||||
Sbjct  1441   GCTTTTCTTCGGAGTATCATTATTCGTTAACTGCTGTTTATCAATAAAATTCCTGATGTT   1500

Query  1501   ATCAAGTGCTTCCTTTGTTTTTAGAATGCGCTTGATATCCATTTCATCCCGCCCGGTCAT   1560
              ||||||||||||||||||||||||||||||||||||||||||||||||||||||||||||
Sbjct  1501   ATCAAGTGCTTCCTTTGTTTTTAGAATGCGCTTGATATCCATTTCATCCCGCCCGGTCAT   1560

Query  1561 TCTTTTTCCGGACAAAAGACGCTCAAGAAGCTCCTGTTTCGCATCAGGAGAATCTTTGGC   1620
              ||||||||||||||||||||||||||||||||||||||||||||||||||||||||||||
Sbjct  1561   TCTTTTTCCGGACAAAAGACGCTCAAGAAGCTCCTGTTTCGCATCAGGAGAATCTTTGGC   1620

Query  1621   GAGATCCATCAGGTTAAGCAAATCTTCACGATCGAGAGGAAAGAAGTATTTTACAAAATA   1680
              ||||||||||||||||||||||||||||||||||||||||||||||||||||||||||||
Sbjct  1621   GAGATCCATCAGGTTAAGCAAATCTTCACGATCGAGAGGAAAGAAGTATTTTACAAAATA   1680

Query  1681 CTTGAAAAGCGAAAGAAAGTAGTATGCTGTCCATGCAATACAGAAACCGATTCTGAGAAA   1740
              ||||||||||||||||||||||||||||||||||||||||||||||||||||||||||||
Sbjct  1681   CTTGAAAAGCGAAAGAAAGTAGTATGCTGTCCATGCAATACAGAAACCGATTCTGAGAAA   1740
```

Query 1741 CTCATTGTCAACTATACAGAAATACAGGTATATAATCAGGAGAATAGGTAAAATCCATAA 1800
||||||||||||||||||||||||||||||||||||||||||||||||||||||||||||
Sbjct 1741 CTCATTGTCAACTATACAGAAATACAGGTATATAATCAGGAGAATAGGTAAAATCCATAA 1800

Query 1801 TACTATAGCTaaaaaaaTATATACGGGGATCAGGCGTCTGTATGCCCGTTCATGTTTATT 1860
||||||||||||||||||||||||||||||||||||||||||||||||||||||||||||
Sbjct 1801 TACTATAGCTAAAAAAATATATACGGGGATCAGGCGTCTGTATGCCCGTTCATGTTTATT 1860

Query 1861 TAGGATGAATAAAATCTTCTCCGCTTCTGAATCACACATAACATTCTCTTCCCATTTTTT 1920
||||||||||||||||||||||||||||||||||||||||||||||||||||||||||||
Sbjct 1861 TAGGATGAATAAAATCTTCTCCGCTTCTGAATCACACATAACATTCTCTTCCCATTTTTT 1920

Query 1921 CTGATACGTAATGTCCACCTGTTATCAGGAGTGTTTATTGCCCAGAAATATTCTCCTGCT 1980
||||||||||||||||||||||||||||||||||||||||||||||||||||||||||||
Sbjct 1921 CTGATACGTAATGTCCACCTGTTATCAGGAGTGTTTATTGCCCAGAAATATTCTCCTGCT 1980

Query 1981 GTGCCTGCATCCTGAGCCACTTTTGTTCTAATGTCGCCAGTCTGCGACCGGCACTCTGTG 2040
||||||||||||||||||||||||||||||||||||||||||||||||||||||||||||
Sbjct 1981 GTGCCTGCATCCTGAGCCACTTTTGTTCTAATGTCGCCAGTCTGCGACCGGCACTCTGTG 2040

Query 2041 CTTCAGAGTCTGAGACCTGCCCTTTGGGGTGACCATCCAGGCCATAACGTGGGACGCCGG 2100
||||||||||||||||||||||||||||||||||||||||||||||||||||||||||||
Sbjct 2041 CTTCAGAGTCTGAGACCTGCCCTTTGGGGTGACCATCCAGGCCATAACGTGGGACGCCGG 2100

Query 2101 CGACAGCTGTCCGCTGCAGATATACGCGGCGGGATGTGATGCAGGACACTGCACAGAGCC 2160
||||||||||||||||||||||||||||||||||||||||||||||||||||||||||||
Sbjct 2101 CGACAGCTGTCCGCTGCAGATATACGCGGCGGGATGTGATGCAGGACACTGCACAGAGCC 2160

Query 2161 ATTCGTCCATGGTCAGTTCTGTCTCCGGGTGTTCTGCCAGAAATGCCGAAATATCATTGT 2220
||||||||||||||||||||||||||||||||||||||||||||||||||||||||||||
Sbjct 2161 ATTCGTCCATGGTCAGTTCTGTCTCCGGGTGTTCTGCCAGAAATGCCGAAATATCATTGT 2220

Query 2221 TTATGCCAATTTTCAGGGGAATAAATCCACCACCTTCAGCACGAAGATGAGGAAACAGGG 2280
||||||||||||||||||||||||||||||||||||||||||||||||||||||||||||
Sbjct 2221 TTATGCCAATTTTCAGGGGAATAAATCCACCACCTTCAGCACGAAGATGAGGAAACAGGG 2280

Query 2281 CCTTTATTTTTTCCACGCACTCGTGAGTATACTCACGGGTCCAGTGGGAGGGACGTGGAA 2340
||||||||||||||||||||||||||||||||||||||||||||||||||||||||||||
Sbjct 2281 CCTTTATTTTTTCCACGCACTCGTGAGTATACTCACGGGTCCAGTGGGAGGGACGTGGAA 2340

Query 2341 AACGGTGTCTTTTCTTTTTCTTCTTCCTGGCTGGCGCTTCGGGGATATGTGTTTCGACGG 2400
||||||||||||||||||||||||||||||||||||||||||||||||||||||||||||
Sbjct 2341 AACGGTGTCTTTTCTTTTTCTTCTTCCTGGCTGGCGCTTCGGGGATATGTGTTTCGACGG 2400

Query 2401 CAGATGAATTTATTCCGGCGGGGGCTGATTCTGCCGTCTGTTCAGTAAATACAGGTGCGA 2460
||||||||||||||||||||||||||||||||||||||||||||||||||||||||||||
Sbjct 2401 CAGATGAATTTATTCCGGCGGGGGCTGATTCTGCCGTCTGTTCAGTAAATACAGGTGCGA 2460

Query 2461 TAATATCTGTTTTTTCGGATAAGGACGGTGGCGAAAAAGTACGACGTTTTTCACCACAA 2520

||||||||||||||||||||||||||||||||||||||||||||||||||||||||||||
Sbjct 2461 TAATATCTGTTTTTTCGGATAAGGACGGTGGCGAAAAAGTACGACGTTTTTTCACCACAA 2520

Query 2521 TGACGGGAGTTTTCCTTTCTTCCTGATATTTTCCCATAAATACCTCGGCTATGTTGTTCT 2580
||||||||||||||||||||||||||||||||||||||||||||||||||||||||||||
Sbjct 2521 TGACGGGAGTTTTCCTTTCTTCCTGATATTTTCCCATAAATACCTCGGCTATGTTGTTCT 2580

Query 2581 ACTTCCTTATAATATCATAGTTCAAAATTTAATAAACTTTCTGTTATGTAATATCCCTTG 2640
||||||||||||||||||||||||||||||||||||||||||||||||||||||||||||
Sbjct 2581 ACTTCCTTATAATATCATAGTTCAAAATTTAATAAACTTTCTGTTATGTAATATCCCTTG 2640

Query 2641 ATAATTTACGAAGATGACTCTGTACCCTGCTATGTGAATCAGGAACGTTTATAGCAGGAT 2700
||||||||||||||||||||||||||||||||||||||||||||||||||||||||||||
Sbjct 2641 ATAATTTACGAAGATGACTCTGTACCCTGCTATGTGAATCAGGAACGTTTATAGCAGGAT 2700

Query 2701 ACAACGGATTGTTACTTACACAGTAAGATTTACTAAGGTTTGTTTTCTTCTTTTCTTATA 2760
||||||||||||||||||||||||||||||||||||||||||||||||||||||||||||
Sbjct 2701 ACAACGGATTGTTACTTACACAGTAAGATTTACTAAGGTTTGTTTTCTTCTTTTCTTATA 2760

Query 2761 TTTATCTGGTAATCCTTATCAACAATGCCTGCTCTACTGTTTGCATTAATCATTGCTGGT 2820
||||||||||||||||||||||||||||||||||||||||||||||||||||||||||||
Sbjct 2761 TTTATCTGGTAATCCTTATCAACAATGCCTGCTCTACTGTTTGCATTAATCATTGCTGGT 2820

Query 2821 CTGATCCATATTAGTTTGTTATTTTTCCTGCGAATATGTCCTCTGCGTAAGTGCATCCTT 2880
||||||||||||||||||||||||||||||||||||||||||||||||||||||||||||
Sbjct 2821 CTGATCCATATTAGTTTGTTATTTTTCCTGCGAATATGTCCTCTGCGTAAGTGCATCCTT 2880

Query 2881 TTCGTCCCACCGGATTTTCCTGTTCCAGTATGATTACTTTGCACCTTAGTTTCAGACAGT 2940
||||||||||||||||||||||||||||||||||||||||||||||||||||||||||||
Sbjct 2881 TTCGTCCCACCGGATTTTCCTGTTCCAGTATGATTACTTTGCACCTTAGTTTCAGACAGT 2940

Query 2941 TGCAATACTTTATAAGTATATGAAGGGCGCTTGACGTATAGGAAATTGAAAAACCAGTCG 3000
||||||||||||||||||||||||||||||||||||||||||||||||||||||||||||
Sbjct 2941 TGCAATACTTTATAAGTATATGAAGGGCGCTTGACGTATAGGAAATTGAAAAACCAGTCG 3000

Query 3001 ATACCATTGTCCACGCCTTATGCCACGTGAGCCTTTCTGCTTTTCCCACCACTCCGGTGG 3060
||||||||||||||||||||||||||||||||||||||||||||||||||||||||||||
Sbjct 3001 ATACCATTGTCCACGCCTTATGCCACGTGAGCCTTTCTGCTTTTCCCACCACTCCGGTGG 3060

Query 3061 TTTCCCTCTCTGTATGGTCGATTTTTATACCGTAACAGGCTGTTTTTTATACTTTCACCG 3120
||||||||||||||||||||||||||||||||||||||||||||||||||||||||||||
Sbjct 3061 TTTCCCTCTCTGTATGGTCGATTTTTATACCGTAACAGGCTGTTTTTTATACTTTCACCG 3120

Query 3121 ATTCCCGGGCACACTTAGCCTGTGTCAGGCCGGCATATACCTCAGTAGACTGATATCCCG 3180
||||||||||||||||||||||||||||||||||||||||||||||||||||||||||||
Sbjct 3121 ATTCCCGGGCACACTTAGCCTGTGTCAGGCCGGCATATACCTCAGTAGACTGATATCCCG 3180

Query 3181 CGCGTTTTTCTTCAGCCCTGATACATTCAGCCCCGCAATGGCCCGGCGGTATACCATCCG 3240
||||||||||||||||||||||||||||||||||||||||||||||||||||||||||||

```
Sbjct  3181  CGCGTTTTTCTTCAGCCCTGATACATTCAGCCCCGCAATGGCCCGGCGGTATACCATCCG  3240

Query  3241  GCAGCCACACAGCACGCATTCGAACGGGTCACGACTCAGGAACTGTTTCACCATTTGTGC  3300
             ||||||||||||||||||||||||||||||||||||||||||||||||||||||||||||
Sbjct  3241  GCAGCCACACAGCACGCATTCGAACGGGTCACGACTCAGGAACTGTTTCACCATTTGTGC  3300

Query  3301  ATAGCACACTTTCGCCACTGGTTCCGGTTTATCCATCCCCAGTGCACGGTACACCTGCGG  3360
             ||||||||||||||||||||||||||||||||||||||||||||||||||||||||||||
Sbjct  3301  ATAGCACACTTTCGCCACTGGTTCCGGTTTATCCATCCCCAGTGCACGGTACACCTGCGG  3360

Query  3361  CAGCTTCTCTCCACACACACGGTTGGCAAGGAACCCGAAGTACCTCACCATCTTAAAAAA  3420
             ||||||||||||||||||||||||||||||||||||||||||||||||||||||||||||
Sbjct  3361  CAGCTTCTCTCCACACACACGGTTGGCAAGGAACCCGAAGTACCTCACCATCTTAAAAAA  3420

Query  3421  CTTCTCCGGGATGTGCTGTTTCAGCCTCGCGACCAGCTCACGCTGTGTCAGCGTTTCCGT  3480
             ||||||||||||||||||||||||||||||||||||||||||||||||||||||||||||
Sbjct  3421  CTTCTCCGGGATGTGCTGTTTCAGCCTCGCGACCAGCTCACGCTGTGTCAGCGTTTCCGT  3480

Query  3481  CGCCGTTTCTCCCGTTTTGTGGTCCAGGTAACGGAAGCTCAGGCTTGCCCCTCCGTTGTA  3540
             ||||||||||||||||||||||||||||||||||||||||||||||||||||||||||||
Sbjct  3481  CGCCGTTTCTCCCGTTTTGTGGTCCAGGTAACGGAAGCTCAGGCTTGCCCCTCCGTTGTA  3540

Query  3541  ATGAGCCAGTCGGGAAGCCGCTATTGGTGGCTTCTTCAGATAACGACCCAGGTAGCGTGC  3600
             ||||||||||||||||||||||||||||||||||||||||||||||||||||||||||||
Sbjct  3541  ATGAGCCAGTCGGGAAGCCGCTATTGGTGGCTTCTTCAGATAACGACCCAGGTAGCGTGC  3600

Query  3601  CGTATTCCGCCCTCCGGCTGTCTTCTTCGACATGTACACATGCCAGTATTTTCCGCCGGA  3660
             ||||||||||||||||||||||||||||||||||||||||||||||||||||||||||||
Sbjct  3601  CGTATTCCGCCCTCCGGCTGTCTTCTTCGACATGTACACATGCCAGTATTTTCCGCCGGA  3660

Query  3661  TTTCAGCACCAGGCTTCTCCACTGTGATTCCGTCGTGATATGTGACAACGACTCCGGCAT  3720
             ||||||||||||||||||||||||||||||||||||||||||||||||||||||||||||
Sbjct  3661  TTTCAGCACCAGGCTTCTCCACTGTGATTCCGTCGTGATATGTGACAACGACTCCGGCAT  3720

Query  3721  TGCCATCCCCTCTGACCACGCTTTCAGAAGCAGCTGCCGCATATTCCACATCCACCGTGA  3780
             ||||||||||||||||||||||||||||||||||||||||||||||||||||||||||||
Sbjct  3721  TGCCATCCCCTCTGACCACGCTTTCAGAAGCAGCTGCCGCATATTCCACATCCACCGTGA  3780

Query  3781  ACGCATCGCGTCTTTCAGGAAGCTCAGCTTTTTCCACTGACCATGCTTATTCAGACCTCC  3840
             ||||||||||||||||||||||||||||||||||||||||||||||||||||||||||||
Sbjct  3781  ACGCATCGCGTCTTTCAGGAAGCTCAGCTTTTTCCACTGACCATGCTTATTCAGACCTCC  3840

Query  3841  ACAGGTTACAGACACATGTACATGCGGATGCCAGTTGAGACGACGGCCATACGTGTGGAT  3900
             ||||||||||||||||||||||||||||||||||||||||||||||||||||||||||||
Sbjct  3841  ACAGGTTACAGACACATGTACATGCGGATGCCAGTTGAGACGACGGCCATACGTGTGGAT  3900

Query  3901  GGCGCAGAAGATACCGGGTTCCAGCCCCCGTTTTCGGGCGGCATACAGCAGATTCTCCAC  3960
             ||||||||||||||||||||||||||||||||||||||||||||||||||||||||||||
Sbjct  3901  GGCGCAGAAGATACCGGGTTCCAGCCCCCGTTTTCGGGCGGCATACAGCAGATTCTCCAC  3960
```

```
Query  3961 CGCCAGACGGCACACGTCATTCAGCAGCCAGCGGTTGCTTTCGAACACCGGCCACAGCGT  4020
            ||||||||||||||||||||||||||||||||||||||||||||||||||||||||||||
Sbjct  3961 CGCCAGACGGCACACGTCATTCAGCAGCCAGCGGTTGCTTTCGAACACCGGCCACAGCGT  4020

Query  4021 GTCCGGCAGGGTGAAGACCAGATGTACCCAGTCGCAGTCAGGAAGACGATTCAGCTGTGT  4080
            ||||||||||||||||||||||||||||||||||||||||||||||||||||||||||||
Sbjct  4021 GTCCGGCAGGGTGAAGACCAGATGTACCCAGTCGCAGTCAGGAAGACGATTCAGCTGTGT  4080

Query  4081 TGCTGTCCACAGGTCTGTGGCCTTCTTTCCGCAGGACGGGCAGGCACGGCTGCCGCATGA  4140
            ||||||||||||||||||||||||||||||||||||||||||||||||||||||||||||
Sbjct  4081 TGCTGTCCACAGGTCTGTGGCCTTCTTTCCGCAGGACGGGCAGGCACGGCTGCCGCATGA  4140

Query  4141 GTTGGTCAGGTACTTTACGTGCTGACAGTCCGGGTTATCACACCCGAACTCTTTTACACC  4200
            ||||||||||||||||||||||||||||||||||||||||||||||||||||||||||||
Sbjct  4141 GTTGGTCAGGTACTTTACGTGCTGACAGTCCGGGTTATCACACCCGAACTCTTTTACACC  4200

Query  4201 CAGTATCCGTGTGCCGCAGGCCAGCATTTTGGTGACGGCTTCAACCTCGATATCGCGCAG  4260
            ||||||||||||||||||||||||||||||||||||||||||||||||||||||||||||
Sbjct  4201 CAGTATCCGTGTGCCGCAGGCCAGCATTTTGGTGACGGCTTCAACCTCGATATCGCGCAG  4260

Query  4261 ACCGCCCGCATCCAGGAAGGACGTCCAGCACTGGTTGGCTGTGAACAGACGTTTCAGAGG  4320
            ||||||||||||||||||||||||||||||||||||||||||||||||||||||||||||
Sbjct  4261 ACCGCCCGCATCCAGGAAGGACGTCCAGCACTGGTTGGCTGTGAACAGACGTTTCAGAGG  4320

Query  4321 GCGGGGAGTAAAACCGGACAACATGAGGATGGATTAAACCCCGGTATCCTGTAGCAGGGT  4380
            ||||||||||||||||||||||||||||||||||||||||||||||||||||||||||||
Sbjct  4321 GCGGGGAGTAAAACCGGACAACATGAGGATGGATTAAACCCCGGTATCCTGTAGCAGGGT  4380

Query  4381 AAGGGCTGCTGTATCGCCACGGCGCTTCAGGATGGAGGTCGTCACCATCGCAGGTGGTGG  4440
            ||||||||||||||||||||||||||||||||||||||||||||||||||||||||||||
Sbjct  4381 AAGGGCTGCTGTATCGCCACGGCGCTTCAGGATGGAGGTCGTCACCATCGCAGGTGGTGG  4440

Query  4441 GTCCATGGGTTTGAGCATTTTCATCCAGTCCCATTCTCGTTGTGAGAGCTTCTCAGCACC  4500
            ||||||||||||||||||||||||||||||||||||||||||||||||||||||||||||
Sbjct  4441 GTCCATGGGTTTGAGCATTTTCATCCAGTCCCATTCTCGTTGTGAGAGCTTCTCAGCACC  4500

Query  4501 AACAGAGAGCAGAACTGTATCGATACTTTTGCGGGTCAACATAAGGGGATGAGTCCTTAA  4560
            ||||||||||||||||||||||||||||||||||||||||||||||||||||||||||||
Sbjct  4501 AACAGAGAGCAGAACTGTATCGATACTTTTGCGGGTCAACATAAGGGGATGAGTCCTTAA  4560

Query  4561 TAAAACAGGTCCTCAGAGCATACATATTCTGCGGGGCGTGCGGCAACAACAGGACGAACC  4620
            ||||||||||||||||||||||||||||||||||||||||||||||||||||||||||||
Sbjct  4561 TAAAACAGGTCCTCAGAGCATACATATTCTGCGGGGCGTGCGGCAACAACAGGACGAACC  4620

Query  4621 GGGGCGGTTTTCAATTTGCAGCCGCCAGGCTGCCGTGGTTCTTGATTATCATATGGCTTT  4680
            ||||||||||||||||||||||||||||||||||||||||||||||||||||||||||||
Sbjct  4621 GGGGCGGTTTTCAATTTGCAGCCGCCAGGCTGCCGTGGTTCTTGATTATCATATGGCTTT  4680
```

```
Query  4681  TGAAACTGTCGCACCTCATGTTTGAATTCGCCCCATATTTTTGCTACAGTGAACCAAATT  4740
             ||||||||||||||||||||||||||||||||||||||||||||||||||||||||||||
Sbjct  4681  TGAAACTGTCGCACCTCATGTTTGAATTCGCCCCATATTTTTGCTACAGTGAACCAAATT  4740

Query  4741  AAGATCATCTATTTACTAGGCCTCGCATTTGCGGGGTTTTTAATGCTGAATAAAAGGAAA  4800
             ||||||||||||||||||||||||||||||||||||||||||||||||||||||||||||
Sbjct  4741  AAGATCATCTATTTACTAGGCCTCGCATTTGCGGGGTTTTTAATGCTGAATAAAAGGAAA  4800

Query  4801  ACTTGATGGAATTGCCCAATATTATGCACCCGGTCGCGAAGCTGAGCACCGCATTAGCCG  4860
             ||||||||||||||||||||||||||||||||||||||||||||||||||||||||||||
Sbjct  4801  ACTTGATGGAATTGCCCAATATTATGCACCCGGTCGCGAAGCTGAGCACCGCATTAGCCG  4860

Query  4861 CTGCATTGATGCTGAGCGGGTGCATGCCCGGTGAAATCCGCCCGACGATTGGCCAGCAAA  4920
             ||||||||||||||||||||||||||||||||||||||||||||||||||||||||||||
Sbjct  4861  CTGCATTGATGCTGAGCGGGTGCATGCCCGGTGAAATCCGCCCGACGATTGGCCAGCAAA  4920

Query  4921 TGGAAACTGGCGACCAACGGTTTGGCGATCTGGTTTTCCGCCAGCTCGCACCGAATGTCT  4980
             ||||||||||||||||||||||||||||||||||||||||||||||||||||||||||||
Sbjct  4921  TGGAAACTGGCGACCAACGGTTTGGCGATCTGGTTTTCCGCCAGCTCGCACCGAATGTCT  4980

Query  4981  GGCAGCACACTTCCTATCTCGACATGCCGGGTTTCGGGGCAGTCGCTTCCAACGGTTTGA  5040
             ||||||||||||||||||||||||||||||||||||||||||||||||||||||||||||
Sbjct  4981  GGCAGCACACTTCCTATCTCGACATGCCGGGTTTCGGGGCAGTCGCTTCCAACGGTTTGA  5040

Query  5041 TCGTCAGGGATGGCGGCCGCGTGCTGGTGGTCGATACCGCCTGGACCAATGACCAGACCG  5100
             ||||||||||||||||||||||||||||||||||||||||||||||||||||||||||||
Sbjct  5041 TCGTCAGGGATGGCGGCCGCGTGCTGGTGGTCGATACCGCCTGGACCAATGACCAGACCG  5100

Query  5101 CCCAGATCCTCAACTGGATCAAGCAGGAGATCAACCTGCCGGTCGCGCTGGCGGTGGTGA  5160
             ||||||||||||||||||||||||||||||||||||||||||||||||||||||||||||
Sbjct  5101 CCCAGATCCTCAACTGGATCAAGCAGGAGATCAACCTGCCGGTCGCGCTGGCGGTGGTGA  5160

Query  5161 CTCACGCGCATCAGGACAAGATGGGCGGTATGGACGCGCTGCATGCGGCGGGGATTGCGA  5220
             ||||||||||||||||||||||||||||||||||||||||||||||||||||||||||||
Sbjct  5161 CTCACGCGCATCAGGACAAGATGGGCGGTATGGACGCGCTGCATGCGGCGGGGATTGCGA  5220

Query  5221 CTTATGCCAATGCGTTGTCGAACCAGCTTGCCCCGCAAGAGGGGCTGGTTGCGGCGCAAC  5280
             ||||||||||||||||||||||||||||||||||||||||||||||||||||||||||||
Sbjct  5221  CTTATGCCAATGCGTTGTCGAACCAGCTTGCCCCGCAAGAGGGGCTGGTTGCGGCGCAAC  5280

Query  5281 ACAGCCTGACTTTCGCCGCCAATGGCTGGGTCGAACCAGCAACCGCGCCCAACTTTGGCC  5340
             ||||||||||||||||||||||||||||||||||||||||||||||||||||||||||||
Sbjct  5281 ACAGCCTGACTTTCGCCGCCAATGGCTGGGTCGAACCAGCAACCGCGCCCAACTTTGGCC  5340

Query  5341  CGCTCAAGGTATTTTACCCCGGCCCCGGCCACACCAGTGACAATATCACCGTTGGGATCG  5400
             ||||||||||||||||||||||||||||||||||||||||||||||||||||||||||||
Sbjct  5341  CGCTCAAGGTATTTTACCCCGGCCCCGGCCACACCAGTGACAATATCACCGTTGGGATCG  5400

Query  5401 ACGGCACCGACATCGCTTTTGGTGGCTGCCTGATCAAGGACAGCAAGGCCAAGTCGCTCG  5460
```

```
                ||||||||||||||||||||||||||||||||||||||||||||||||||||||||||||
Sbjct  5401 ACGGCACCGACATCGCTTTTGGTGGCTGCCTGATCAAGGACAGCAAGGCCAAGTCGCTCG  5460

Query  5461 GCAATCTCGGTGATGCCGACACTGAGCACTACGCCGCGTCAGCGCGCGCGTTTGGTGCGG  5520
            ||||||||||||||||||||||||||||||||||||||||||||||||||||||||||||
Sbjct  5461 GCAATCTCGGTGATGCCGACACTGAGCACTACGCCGCGTCAGCGCGCGCGTTTGGTGCGG  5520

Query  5521 CGTTCCCCAAGGCCAGCATGATCGTGATGAGCCATTCCGCCCCCGATAGCCGCGCCGCAA  5580
            ||||||||||||||||||||||||||||||||||||||||||||||||||||||||||||
Sbjct  5521 CGTTCCCCAAGGCCAGCATGATCGTGATGAGCCATTCCGCCCCCGATAGCCGCGCCGCAA  5580

Query  5581 TCACTCATACGGCCCGCATGGCCGACAAGCTGCGCTGA  5618
            ||||||||||||||||||||||||||||||||||||||
Sbjct  5581 TCACTCATACGGCCCGCATGGCCGACAAGCTGCGCTGA  5618



Lambda      K        H
   1.33    0.621     1.12

Gapped
Lambda      K        H
   1.28    0.460    0.850

Effective search space used: 31371201


  Database: User specified sequence set (Input:
.\pHNAHS65I-1_copG-blaNDM-13.fasta).
    Posted date:  Unknown
  Number of letters in database: 5,618
  Number of sequences in database:  1



Matrix: blastn matrix 1 -2
Gap Penalties: Existence: 0, Extension: 2.5
```

Re: Spectrum03207-24R3 (**Carbapenem Resistance Mediated by *bla*$_{NDM-13}$ in a Highly Drug-Resistant *Salmonella* Stanley ST29 strain in China**)

Dear Mrs. Juanjuan Zhou:

Your manuscript has been accepted, and I am forwarding it to the ASM production staff for publication. Your paper will first be checked to make sure all elements meet the technical requirements. ASM staff will contact you if anything needs to be revised before copyediting and production can begin. Otherwise, you will be notified when your proofs are ready to be viewed.

Data Availability: ASM policy requires that data be available to the public upon online posting of the article, so please verify all links to sequence records, if present, and make sure that each number retrieves the full record of the data. If a new accession number is not linked or a link is broken, provide production staff with the correct URL for the record. If the accession numbers for new data are not publicly accessible before the expected online posting of the article, publication may be delayed; please contact ASM production staff immediately with the expected release date.

Publication Fees: For information on publication fees and which article types have charges, please visit our website. We have partnered with Copyright Clearance Center (CCC) to collect author charges. If fees apply to your paper, you will receive a message from no-reply@copyright.com with further instructions. For questions related to paying charges through RightsLink, please contact CCC at ASM_Support@copyright.com or toll free at +1-877-622-5543. CCC makes every attempt to respond to all emails within 24 hours.

ASM Membership: Corresponding authors may join or renew ASM membership to obtain discounts on publication fees. Need to upgrade your membership level? Please contact Customer Service at Service@asmusa.org.

Embargo Policy: A press release may be issued as soon as the manuscript is posted on the Spectrum Latest Articles webpage. The corresponding author will receive an email with the subject line "ASM Journals Author Services Notification" when the article is available online.

The ASM Journals program strives for constant improvement in our submission and publication process. Please tell us how we can improve your experience by taking this quick Author Survey.

Thank you for submitting your paper to Spectrum.

Sincerely,
Jian Peng
Editor
Microbiology Spectrum

Reviewer #2 (Comments for the Author):

No further comments
